# American highbush cranberry maintains strong population structure despite naturalization of Eurasian relatives in North America

**DOI:** 10.1002/ajb2.70124

**Published:** 2025-11-14

**Authors:** David G. Tork, Neil O. Anderson, Anthony Brusa, Alan G. Smith

**Affiliations:** ^1^ Department of Horticultural Science University of Minnesota 305 Alderman Hall, 1970 Folwell Avenue Saint Paul 55108 Minnesota USA; ^2^ Department of Agronomy and Plant Genetics University of Minnesota 411 Borlaug Hall, 1991 Upper Buford Circle Saint Paul 55108 Minnesota USA

**Keywords:** Adoxaceae, clonality, cryptic species, DArTseq, genetic structure, hybridization, invasive, Viburnum opulus, Viburnum sargentii, Viburnum trilobum

## Abstract

**Premise:**

The proper classification of taxa is often debated, particularly when organisms lack qualitative diagnostic traits. Highbush cranberry taxa (*Viburnum* spp.) have been the subject of such disputes since their characterization by 18th‐ and 19th‐century botanists. Despite their allopatric distributions—*V. trilobum* in North America, *V. opulus* in Europe, and *V. sargentii* in Asia—these taxa have received numerous taxonomic treatments as species, subspecies, and varieties due to their morphological similarities. Genetic evidence has shown these taxa to be distinct; however, the human‐mediated introduction of *V. opulus* and *V. sargentii* into North America may remove their geographic and genetic isolation, with implications for the conservation of *V. trilobum*.

**Methods:**

We used single‐nucleotide polymorphisms generated via reduced representation sequencing (DArTseq) to assess the genetic structure and diversity of these taxa, focusing on the impact of *V. opulus* and *V. sargentii* introduction into North America.

**Results:**

Consistent with prior studies, *V. trilobum*, *V. opulus*, and *V. sargentii* were found to be genetically distinct species. European *V. opulus*—and, to a lesser extent, *V. opulus* × *V. sargentii* hybrids—were found to be widely naturalizing in North America. However, interspecific *V. trilobum* hybrids were notably rare. All three taxa exhibited low genetic diversity and evidence of clonality. A cryptic subgroup of *V. sargentii*, originating in Japan, was identified.

**Conclusions:**

American *V. trilobum* shows evidence of continued genetic isolation despite the introduction of Eurasian taxa into North America, suggesting the presence of an unknown reproductive barrier.

There is no single definition of *species*. Numerous species concepts exist, applied by taxonomists to delineate biologically meaningful categorizations of natural populations (Zachos, 2016; Aldhebiani, 2018). Although many species are defined on the basis of morphological characteristics or diagnostic traits, the process of speciation does not always result in morphological divergence, leading to taxonomic confusion (Bickford et al., 2007; Rouhan and Gaudeul, 2021; Shin and Allmon, 2023). These “cryptic” or “sibling” species are variously defined but may be generally characterized as species that are morphologically difficult to identify (Struck and Cerca, 2022; Shin and Allmon, 2023). The advent of genomic sequencing technologies has revealed that cryptic species are far more numerous and ecologically relevant than previously thought, raising important questions about the taxonomy and conservation of these taxa (Bickford et al., 2007; Hending, 2025).

Accurate taxonomic definitions are critical for research and biodiversity conservation, especially in regard to cryptic species, which often lack formal taxonomic descriptions (Shin and Allmon, 2023). Whether a biological population is recognized as taxonomically distinct often determines its inclusion in ecological research, conservation assessments, regulations, seed banking programs, and habitat restoration efforts (May, 1990; Bortolus, 2008; Garnett and Christidis, 2017; Abrahamse et al., 2021). Taxonomic confusion hampers ex situ conservation efforts by obscuring underrepresented taxa, causing misidentifications, and reducing the overall genetic diversity represented in the collection, especially for taxa that have undergone revision (Guzzon and Ardenghi, 2018). Conservation assessments may underreport threats to cryptic species if they are not distinguished from established taxa (Bickford et al., 2007; Liu et al., 2022). Similarly, invasions of cryptic species may go undetected, and, in the worst cases, taxonomic and morphological confusion can lead to the unintended establishment of a nonnative or invasive species by a conservation program (Kittleson and Boyd, 1997; Saltonstall, 2002; Bickford et al., 2007; Morais and Reichard, 2018). These issues highlight the importance of proper taxonomic characterization of biological diversity.

The highbush cranberry group (*Viburnum* spp.; Adoxaceae) consists of three morphologically similar taxa that have been the subject of taxonomic debate and revision for several hundred years due to their cryptic nature. Taxonomists have generally recognized these as distinct taxa due to their allopatric distributions in North America, Europe, and Asia (Hultén and Fries, 1986). Their morphological similarity, however, has resulted in various taxonomic treatments, ranging from separate species to subspecies or varieties of a single taxon (Youngken, 1932; Hara, 1983; Nellessen, 2006; Qiner et al., 2011). For clarity, we will refer to these taxa as species defined by their native distributions in the cold temperate regions North America (*V. trilobum* Marsh.), Europe (*V. opulus* L.), and Asia (*V. sargentii* Koehne), although readers should be aware that taxonomic synonyms remain common in the literature (Ohashi, 1994; Kollmann and Grubb, 2002; Nellessen, 2006; Qiner et al., 2011; Chang et al., 2014; Choi et al., 2018; Landis et al., 2021).

These highbush cranberry species belong to the *Opulus* clade of *Viburnum*, within which they form a sister clade to the “lowbush cranberry” species, *V. edule* Michx. and *V. koreanum* Nakai (Clement et al., 2014; Landis et al., 2021). All members of the *Opulus* clade are characterized by lobed leaves, red drupes, connate bud scales, and small extrafloral nectaries (EFNs) where the leaf meets the petiole (Rehder, 1908; Qiner et al., 2011; Weber et al., 2012). The debate over the proper taxonomic ranking of *V. trilobum*, *V. opulus*, and *V. sargentii* stems from the fact that all three subtypes can be equally characterized as upright, multi‐stemmed shrubs (Figure 1A–C), with compound inflorescences encircled by enlarged sterile marginal flowers (Figure 1D–F), clusters of drupes ripening to bright red (Figure 1G–I), and three‐lobed leaves resembling *Acer* (Figure 1J–L). Morphological distinctions among the three taxa are based on subtle morphological traits, the most cited being the size and shape of the EFNs. These are reported as convex and occasionally stalked in *V. trilobum* (Figure 1M), while concave and sessile in *V. opulus* and *V. sargentii* (Figure 1N, O; Youngken, 1932; Rehder, 1962; Gleason and Cronquist, 1963; Hara, 1983). However, the diagnostic validity of EFN shape has been questioned due to observed variation (McAtee, 1956; Voss, 1996). The morphological differences between *V. opulus* and *V. sargentii* are even less clear. *Viburnum sargentii* is described as having corky bark and purple anthers (Rehder, 1903, 1962; Hara, 1983; Qiner et al., 2011), although the uniformity of anther color has been disputed (Dirr, 2007). These subtle phenotypic traits complicate identification and have led many taxonomists to lump these taxa as subspecies or varieties of *V. opulus* (Aiton, 1789; McAtee, 1956; Hara, 1983; Ohashi, 1994; Qiner et al., 2011).

**Figure 1:**
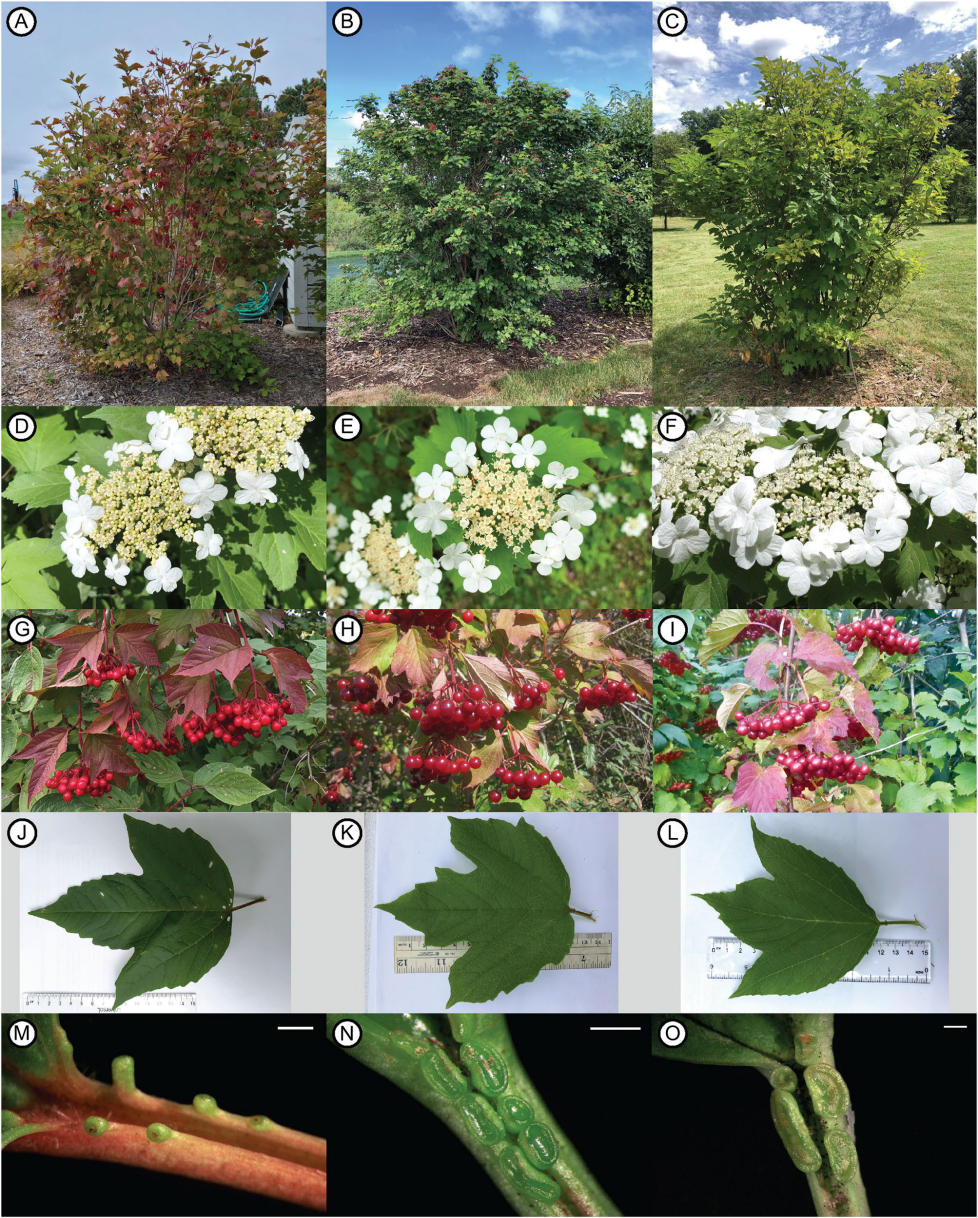
Comparative morphology of highbush cranberry species *Viburnum trilobum* (A, D, G, J, M), *V. opulus* (B, E, H, K, N), and *V. sargentii* (C, F, I, L, O). All three species exhibit similar growth habit (A–C), flower morphology (D–F), fruit morphology (G–I), and leaf morphology (J–L). The North American (*V. trilobum*) and Eurasian (*V. opulus* and *V. sargentii*) species are differentiated morphologically by small extrafloral nectaries (EFNs) located at the base of the leaf blade. In *V. trilobum* (M), these EFNs are round, convex, and occasionally stalked; whereas in *V. opulus* (N) and *V. sargentii* (O) the EFNs are reniform, concave, and usually sessile. Scale bars in M–O: 1 mm. Photo credits: (F) Jon Hetman, *Viburnum opulus* ssp. *calvescens* 719‐88*A at the Arnold Arboretum ©2018 President and Fellows of Harvard College; Creative Commons License: Attribution‐NonCommercial 4.0 International (CC BY‐NC 4.0); (H) Bill Moses, volunteer collector; (I) Bob Mayer, *Viburnum sargentii* 1922‐80*C at the Arnold Arboretum ©2021 President and Fellows of Harvard College; Creative Commons License: Attribution‐NonCommercial 4.0 International (CC BY‐NC 4.0).

Despite these morphological similarities, genetic data provide insights that strengthen the classification of highbush cranberry taxa as species. Phylogenetic analyses of *Viburnum* based on barcoding gene sequences (Clement and Donoghue, 2011), whole plastid genomes (Clement et al., 2014; Spriggs et al., 2015), and RAD‐seq data (Landis et al., 2021) have all shown that *V. trilobum*, *V. opulus*, and *V. sargentii* are genetically distinct. Importantly, both nuclear and chloroplast DNA sequence data are concordant in resolving *V. opulus* and *V. sargentii* as sister taxa, indicating that *V. trilobum* is more distantly related. Estimates of divergence times based on RAD‐seq data suggest that *V. opulus* and *V. sargentii* separated within the past 5 million years, while *V. trilobum* diverged from both ~15 million years ago (Landis et al., 2021). These studies provide strong evidence that these taxa have a long history of geographic and genetic isolation. The combination of extensive divergence times and morphological similarity meet the criteria for the classification of *V. trilobum*, *V. opulus*, and *V. sargentii* as cryptic species (Struck and Cerca, 2022).

Present taxonomic classification of highbush cranberry viburnums is further complicated by the introduction of *V. opulus* and *V. sargentii* into North America as landscape plants, and their potential for hybridization with *V. trilobum*. It has become widely recognized that *V. opulus* frequently escapes cultivation in North America through seed dispersal by birds and mammals (Witmer, 2001; Nellessen, 2006). Presently, there are 3224 reports of naturalized *V. opulus* populations, overlapping almost entirely with the native range of *V. trilobum* (Hultén and Fries, 1986; EDDMapS, 2025). Furthermore, the distribution of *V. opulus* is likely underreported in North America due to the morphological similarity with *V. trilobum* (Figure 1; Martine et al., 2008). As a result, *V. opulus* is now considered invasive in several states for its ability to compete with and replace *V. trilobum* (Nellessen, 2006; IISC, 2023; Pennsylvania DCNR, 2023; WVDNR, 2025). It is not currently known whether *V. sargentii* is also naturalizing in North America. Compared to *V. opulus*, *V. sargentii* has not been as widely planted in landscapes (Dirr, 2007), although it is possible that naturalizing populations might be misclassified as *V. opulus* due to the similarity of their EFN morphology (Figure 1N, O). To complicate matters, *V. opulus* and *V. trilobum* are capable of hybridizing (Egolf, 1956). This potential is supported by cytological evidence, as all three taxa are diploid (2n = 18) and possess similar genome sizes (Egolf, 1962; Moeglein et al., 2020). It has thus been suggested that the *V. trilobum* gene pool may be undergoing extinction through hybridization (Wolf et al., 2001; Nellessen, 2006; Todesco et al., 2016). The naturalization of these Eurasian species, and their potential to hybridize with *V. trilobum*, complicates species concepts based on the geographic and reproductive isolation among these taxa. Although the extent of these changes remains poorly characterized, their potential consequences pose an ongoing threat to the conservation of *V. trilobum*.

The need for American highbush cranberry conservation arises not only from its ecological importance (Zach et al., 1982; Petrides, 1986; Thompson et al., 1992; Nellessen, 2006), but also from its cultural and culinary significance. *Viburnum trilobum* has historically been used for food and medicine by Indigenous peoples in North America (Kuhnlein and Turner, 1991; Moerman, 1998; Turner, 2007). The fruit of American highbush cranberry (*V. trilobum*) bears resemblance in flavor to commercial cranberries (*Vaccinium* subg. *Oxycoccus*), from which it derives its common name. Today, *V. trilobum* fruit are still harvested from wild stands by foragers and small‐scale manufacturers to produce jellies, syrups, and other preserved goods (Darrow, 1923; Small, 2014; Thayer, 2023). In contrast, European *V. opulus* produces comparatively bitter fruit that is undesirable for culinary uses. Confusing morphology, inconsistent taxonomic treatments, and concerns about hybridization with *V. opulus* have created confusion about the edibility of *V. trilobum* (Small, 2014; Thayer, 2023). Without an improved understanding of these species and subsequent conservation efforts, it seems likely that the cultural knowledge of *V. trilobum* will also be eroded over time.

To date, no study has genotyped wild populations of highbush cranberry in North America to assess the presence of exotic *V. opulus* and *V. sargentii* and their rates of hybridization with *V. trilobum*. Therefore, our goal was to evaluate the genetic structure and diversity of highbush cranberry specimens throughout North America, as well as from the native ranges of *V. opulus* and *V. sargentii* in Europe and Asia, respectively. We employed reduced‐representation sequencing (DArTseq), a high‐throughput genotyping approach that generates thousands of genome‐wide markers suitable for detecting fine‐scale genetic structure and admixture. This approach has been used successfully across diverse plant taxa to estimate genetic diversity, identify hybridization, resolve closely related lineages, and correct taxonomic misidentifications (Sansaloni et al., 2011; Noyszewski et al., 2021; Wilson et al., 2022; Jordan et al., 2024; Palsson et al., 2024). Using these data, we examine whether the highbush cranberry taxa are maintaining reproductive isolation despite their human‐mediated sympatry in North America. Our findings are discussed in the context of taxonomic debates, along with implications for the conservation and evolutionary trajectories of *V. trilobum*, *V. opulus*, and *V. sargentii*.

## MATERIALS AND METHODS

### Sample collection

The species targeted for collection included members of the *Opulus* clade of *Viburnum* (Landis et al., 2021). The primary goal of the study was to examine whether American highbush cranberry (*V. trilobum*) is genetically distinct from its European (*V. opulus*) and Asian (*V. sargentii*) relatives, despite naturalization of the latter two species in North America. A closely related species, lowbush cranberry (*V. edule*), was collected as an outgroup.

Specimens were collected between June and November 2022. For all living specimens, a total of four leaves per plant were collected from a single stem and placed into bags containing 30 mL of silica‐gel desiccant. Up to 10 samples were collected per population, with ~10 m between samples to prevent collecting multiple clonal samples from the same individual. Separate populations were defined as having >1 km distance between them. At the time of collection, data were recorded using the mobile application Survey123 (ESRI, ArcGIS Survey123, version 3.16.114 or earlier). After assigning each sample a unique sample ID, collectors recorded the following data: collector name, date of collection, reported species ID, GPS coordinates, plant setting (wild, landscape, arboretum/botanical garden, nursery, herbarium), location description, phenotypic data, notes about the specimen, and photos of the plant morphology.

For specimens originating from outside sources (nurseries, arboreta, botanical gardens, herbaria), sample data were compiled manually, if available, from the parent institution's database and/or materials provided with the specimens. For accessions lacking GPS coordinates, the original location of the collection was estimated from written descriptions, if available. In the case of herbarium collections, a single leaf was removed from existing voucher specimens. Accession numbers for all specimens sourced from living collections or herbaria are listed in Appendix S1, with herbarium vouchers specifically listed under the institution codes MIN, MOR, and K.

### Laboratory procedures

For all samples, 10–20 mg of tissue was subsampled and placed into racked microtubes (1.1 mL Microtube System, Racked 15082; Thermo Scientific, Waltham, Massachusetts, USA) containing a 3 mm stainless steel bead (22.455.0011; RETSCH GmbH, Haan, Germany). A total of 1034 samples were prepared, consisting of 1023 unique specimens with 11 biological replicates (repeated samples from the same leaf). Tools used for processing tissue were cleaned in soapy water and rinsed twice in deionized water between samples. Work surfaces were wiped clean of debris using 70% ethanol between each sample. Tissue was pulverized using a homogenizer (Geno/Grinder; SPEX SamplePrep, Metuchen, New Jersey, USA) for 10 min at 1500 rpm. Pulverized samples were sent to Diversity Arrays Technology Pty Ltd (DArT; University of Canberra, Bruce, Australia) for DNA extraction and genotyping service.

DNA extraction and genotyping were performed by DArT using their DArTseq genotyping‐by‐sequencing platform (Sansaloni et al., 2011). Genomic complexity was reduced via co‐digestion with the methylation‐sensitive restriction enzyme *Pst*I and a frequent‐cutting secondary restriction enzyme to enrich for low‐copy genomic regions. Single‐nucleotide polymorphism (SNP) calling and marker filtering were performed using DArT's proprietary analytical pipeline, resulting in a low‐density set of genome‐wide SNP markers, primarily from the nuclear genome (DArTseqLD). Prior to downstream analyses, SNP data were further filtered in R version 4.4.3 or earlier (R Core Team, 2025) using the package dartR version 2.9.8 (Gruber et al., 2018; Mijangos et al., 2022). Loci with >28% missing data and a reproducibility score <98% were removed. To reduce linkage, only one SNP per sequence fragment was retained, prioritizing the SNP with the highest reproducibility. Individuals with a call rate <25% were also excluded, resulting in the removal of six low‐quality samples. Lastly, monomorphic loci produced by prior filtering steps were removed.

### Population structure analysis

Genetic structure was visualized using principal component analysis (PCA) implemented in the R package dartR version 2.9.8 (Gruber et al., 2018; Mijangos et al., 2022). Further analysis of population structure was conducted using the Bayesian clustering method implemented in structure version 2.3.4 (Pritchard et al., 2000; Falush et al., 2003). Ancestry coefficients (*q*‐values) were estimated for each sample according to the number of clusters (*K*). Values of *K* from 1–10 were tested with 10 independent runs each, using an admixture model with a burn‐in period of 50,000 and 500,000 Markov chain Monte Carlo (MCMC) replications after burn‐in. The optimal *K* value was estimated by the Δ*K* statistic (Evanno et al., 2005). structure results were visualized in R using the package pophelper version 2.3.1 (Francis, 2017).

### Genetic identification of specimens

Genetic re‐identification and hybrid assignment was based on structure results for *K* = 4 clusters, the expected value based on the number of species included (*V. trilobum*, *V. opulus*, *V. sargentii*, or *V. edule*). The four clusters (*K*) identified in structure were assigned to these species according to the majority grouping provided by the reported identifications. Individuals showing admixture (i.e., membership in two clusters) were classified as hybrids of *V. trilobum* × *V. opulus* (Vt × Vo), *V. trilobum* × *V. sargentii* (Vt × Vs), or *V. opulus* × *V. sargentii* (Vo × Vs). Individuals were identified as hybrids only if their *q*‐value for a secondary cluster (*K*) exceeded the threshold of *q* ≥ 0.0625, whereas any individual with a secondary *q*‐value <0.0625 was classified as a parental species. Based on simulation studies, this threshold should maximize detection of advanced generation hybrids (F_2_, backcross, or greater) and minimize misidentification of non‐admixed individuals as hybrids (Vähä and Primmer, 2006; Bohling et al., 2013; Van Wyk et al., 2017).

Additional validation of the genetic identifications based on *K* = 4 clusters was performed by running independent pairwise structure analyses of the three main species groups *V. trilobum*, *V. opulus*, and *V. sargentii* (Pritchard et al., 2000; Falush et al., 2003). After correcting sample identifications using *K* = 4 results, the full SNP data set was subset using dartR version 2.9.8 (Gruber et al., 2018; Mijangos et al., 2022) to produce three files: (1) *V. trilobum*, *V. opulus*, and Vt × Vo; (2) *V. opulus*, *V. sargentii*, and Vo × Vs; (3) *V. trilobum*, *V. sargentii*, and Vt × Vs. For each file, *K* values 1–10 were tested with 10 independent runs each using an admixture model with a burn‐in period of 50,000 and 500,000 MCMC replications. The optimal *K* value was estimated using Δ*K* and the results visualized using pophelper version 2.3.1 (Francis, 2017). Cluster assignments and admixed individuals from pairwise structure analyses were compared to structure results from the full SNP data set to ensure consistent results. Genetic identifications based on structure analyses were used to determine population groupings in all downstream analyses.

### Phylogenetic network analysis

A phylogenetic split network visualization was generated using SplitsTree App version 6.4.13 (Huson and Bryant, 2024) using the Neighbor Net method (Bryant, 2003; Bryant and Huson, 2023) to generate splits based on a P distance matrix (Hamming, 1950). Branch tips were colored based on genetic identifications to visualize phylogenetic relationships of specimens within and among genetic groups. Parallel edges represent splits computed from the data (Huson and Bryant, 2006).

### Population structure analysis within species

Evidence for population structure within species was examined separately for *V. trilobum*, *V. opulus*, and *V. sargentii*. The full SNP data set was subset using dartR version 2.9.8 (Gruber et al., 2018; Mijangos et al., 2022) to produce separate files for *V. trilobum*, *V. opulus*, and *V. sargentii*. For each file, *K* values 1–6 were tested with 10 independent runs each using an admixture model with a burn‐in period of 50,000 and 500,000 MCMC replications. The optimal *K* value was estimated using Δ*K* and the results visualized using pophelper version 2.3.1 (Francis, 2017).

A separate PCA analysis was run for *V. sargentii* using dartR version 2.9.8 (Gruber et al., 2018; Mijangos et al., 2022) to examine evidence for genetic structure based on the geographic origin of specimens.

### Geographic context of results

Genetically identified samples with known provenance information (Appendix S1) were mapped to examine the congruity between each species’ historic native range and the contemporary distributions of genetically identified material, emphasizing the spread of *V. opulus* and *V. sargentii* genotypes in North America. Mapping was performed in R version 4.4.3 or earlier (R Core Team, 2025) using functions from packages ggplot2 version 3.5.1 (Wickham, 2016) and ggspatial version 1.1.9 (Dunnington, 2023). All maps use the WGS84 coordinate system. Sample location data in latitude and longitude were converted to the WGS84 coordinate system using R package sf version 1.0‐19 (Pebesma, 2018; Pebesma and Bivand, 2023). The packages rnaturalearth version 1.0.1 (Massicotte and South, 2023) and maps version 3.4.2.1 (Becker et al., 2024) were used to obtain base map files.

Separate maps were created for *V. trilobum*, *V. opulus*, and *V. sargentii* to demonstrate genetic structure based on geographic origin of specimens. Cluster assignments (*q*‐values) obtained from structure were averaged for any specimens originating from the same geographic population using the R package dplyr version 1.1.4 (Wickham et al., 2023). The method used for plotting was identical to the above, except that sample points were plotted using the package scatterpie version 0.2.4 (Yu, 2024) to show average cluster assignments for *K* = 2 clusters.

### Genetic diversity estimates

Basic summary statistics of genetic diversity were estimated for each genetically identified population. The mean observed heterozygosity (*H*_O_), expected heterozygosity (*H*_E_), and inbreeding coefficient (*F*_IS_) were calculated using dartR version 2.9.8 (Gruber et al., 2018; Mijangos et al., 2022). Mean allelic richness was calculated using R package hierfstat version 0.5‐11 (Goudet and Jombart, 2022). Pairwise calculations of among population genetic fixation and allelic differentiation, respectively, were calculated as *G'*_ST_ (Nei, 1987) and *D*_EST_ (Jost, 2008) using dartR version 2.9.8 (Gruber et al., 2018; Mijangos et al., 2022).

Analysis of molecular variance (AMOVA; Excoffier et al., 1992) was performed to test among‐ and within‐population partitioning of variance using the R package poppr version 2.9.6 (Kamvar et al., 2014, 2015) using the ade4 implementation. Significance was calculated using ade4 version 1.7‐22 (Dray and Dufour, 2007) with 9999 permutations.

### Clonality

The presence of clonality was evaluated using the R package poppr version 2.9.6 (Kamvar et al., 2014, 2015). Determination of clonality was based on the grouping of 11 biological replicates (duplicate samples obtained from the same leaf) into identical multilocus lineages (MLLs). The default method used by poppr to detect multilocus genotypes (MLGs) assumes no genetic differences, including missing data, resulting in all 995 specimens (including biological replicates) being grouped into separate MLGs, presumably due to minor sequencing errors. Thus, MLGs were collapsed into MLLs based on bitwise genetic distance (Prevosti et al., 1975; Kamvar et al., 2015) until all known replicates were grouped within the same MLL (i.e., identified as clones in the analysis). Three clustering algorithms—farthest neighbor, average neighbor (UPGMA), and nearest neighbor—were evaluated, ranging from most to least conservative in their groupings of MLLs, respectively (Kamvar et al., 2015). First, a histogram of pairwise genetic distances (bitwise distance) among samples was generated using the function “filter_stats()” to assess the ability of each algorithm to collapse MLGs based on genetic distance threshold. Then various distance thresholds were tested independently for each algorithm until all known replicates were paired into identical MLLs. The number of distinct MLGs in each population was summarized for each algorithm as an estimate of clonality using the chosen genetic distance threshold.

## RESULTS

### Sample collection

Leaf samples from a total of 1023 unique genotypes were collected for the study. Roughly half of these were sourced from wild or naturalized populations (497 samples) collected in the Great Lakes region of North America, where naturalization of the Eurasian taxa is widespread. The remaining samples were sourced from arboreta or botanical gardens (300 samples), commercial nurseries (86 samples), herbaria (86 samples), and landscape installations (54 samples). Of the 1023 specimens, 776 were determined to have known provenance, meaning either (1) that, to the best of our knowledge, the specimen was established at the collection location through natural dispersal; or (2) that thorough documentation exists regarding the original collection location of an ex situ specimen (e.g., a specimen growing at an arboretum that originated from cuttings or seed collected from a known location). The remaining 247 specimens were of unknown provenance (i.e., human‐established plants of an unknown geographic origin, including cultivars or landscape plants of unknown background). A total of 83 specimens from 28 unique named cultivars were included in the study to examine the prevalence of misidentification among cultivated plants. The full list of specimens, including cultivar names, is provided in Appendix S1.

### SNP calling

Of the 1034 specimens submitted to DArT, genotyping data were obtained for 1001 of these specimens, with 33 samples failing either DNA extraction or DArT's quality control pipeline. The unfiltered SNP data set consisted of 19,129 SNPs. Additional filtering reduced the data set to 995 specimens with 10,720 total SNPs, which was the basis for all downstream analyses.

The files subset for pairwise species comparisons were as follows: (1) *V. trilobum*, *V. opulus*, and Vt × Vo hybrids containing 842 individuals and 6633 loci; (2) *V. opulus*, *V. sargentii*, and Vo × Vs hybrids containing 545 individuals and 6552 loci; (3) *V. trilobum*, *V. sargentii*, and Vt × Vs hybrids containing 517 individuals and 7330 loci.

File subsets used for intraspecific analyses included (1) *V. trilobum* containing 425 individuals and 3251 loci, (2) *V. opulus* containing 409 individuals and 3063 loci, and (3) *V. sargentii* containing 91 individuals and 3302 loci.

### Genetic re‐identification of samples and population structure

The reported species identifications assigned at the time of collection were considered in initial exploratory analyses used to connect genetic clusters with their associated taxonomic group. An initial PCA with populations assigned based on the reported ID showed four primary clusters corresponding to the four reported species in this analysis: *V. trilobum*, *V. opulus*, *V. sargentii*, and *V. edule*; however, each cluster also contained off‐types suspected to be misidentified samples. Points plotted between the primary *V. trilobum*, *V. opulus*, and *V. sargentii* clusters also suggested the presence of admixed individuals (Appendix S2).

**Figure 2:**
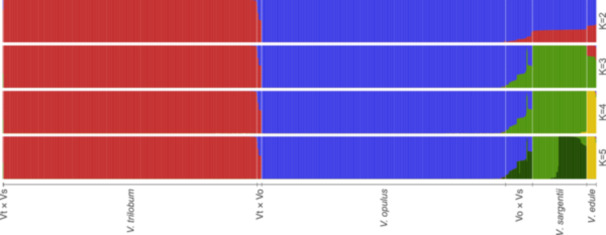
Structure bar plots showing membership proportions for all highbush cranberry (*Viburnum* spp.) individuals from *K* = 2 through *K* = 5 clusters. Admixed zones based on the *K* = 4 model are defined by vertical white lines.

Misidentified and hybrid samples were re‐identified based on the results of structure analysis. The models with the highest Δ*K* support were *K* = 2 followed by *K* = 3 clusters (Appendix S3). The *K* = 2 model differentiated *V. trilobum* and *V. opulus*, while *K* = 3 further differentiated *V. sargentii* from the previous two species. The much smaller *V. edule* group, containing only 16 samples, was distinguished from the other species only when considering *K* = 4 clusters (Figure 2). Thus, *K* = 4 clusters were used to recode all samples as either *V. trilobum*, *V. opulus*, *V. sargentii*, *V. edule*, or admixed hybrids, based on their membership proportion to each population.

Separate, pairwise structure analyses of the three main genetically identified species (*V. trilobum*, *V. opulus*, *V. sargentii*) all showed the highest Δ*K* support for *K* = 2 clusters (Appendix S4). Additionally, the same individuals were identified as admixed when applying the same hybrid calling threshold of ≥6.25% membership in a second cluster.

**Figure 3:**
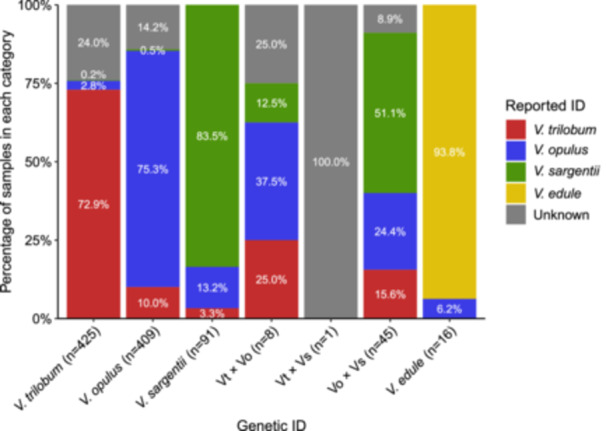
Comparison of genetic and reported identifications showing the percentage of correctly and incorrectly identified highbush cranberry (*Viburnum* spp.) specimens in each category. The total number of samples in each genetically identified taxa is denoted by (*n*) within the *x*‐axis labels.

Of the 995 samples genetically identified, a total of 710 were correctly identified by collectors, 119 were misidentified, and 166 were reported as “Unknown” (Figure 3). A total of 941 (94.6%) specimens were identified as non‐admixed species, while 54 (5.4%) were identified as hybrids. Of the three main species, *V. trilobum* had the smallest percentage of misidentified specimens, though it contained the greatest number of specimens reported as unknown. In contrast, both *V. opulus* and *V. sargentii* had a relatively high percentage of misidentified samples (10.5% and 16.5%, respectively). The hybrids Vt × Vo and Vo × Vs posed a particular challenge to field identification and were identified inconsistently as belonging to any one of the three main species groups (Figure 3).

**Figure 4:**
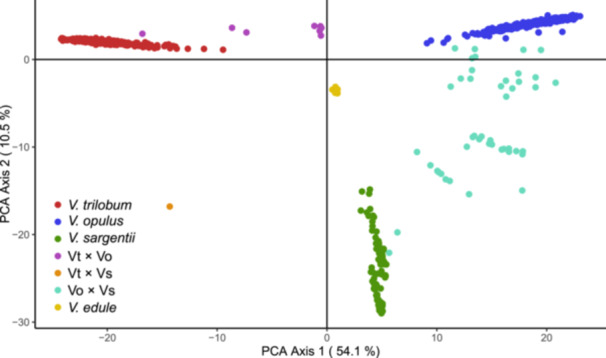
Principal component analysis (PCA) axes 1 and 2 for 995 highbush cranberry specimens, with populations assigned on the basis of genetic identifications from Structure using *K* = 4 clusters. Hybrid groups include *Viburnum trilobum* × *V. opulus* (Vt × Vo), *V. trilobum* × *V. sargentii* (Vt × Vs), and *V. opulus* × *V. sargentii* (Vo × Vs).

The sample identifications derived from structure analysis were largely congruent with clustering patterns observed in the PCA plot. Four primary clusters are apparent in the PCA corresponding to the four taxa (*V. trilobum*, *V. opulus*, *V. sargentii*, and *V. edule*). Hybrids of *V. trilobum*, *V. opulus*, and *V. sargentii* are plotted intermediate to the main species clusters. The first PCA axis explained the majority of variation (54.1%) and distinctly separated *V. trilobum* from its Eurasian relatives, *V. opulus* and *V. sargentii* (Figure 4). The second axis accounted for 10.5% of variation and differentiated *V. sargentii* from *V. opulus* (Figure 4). Finally, although the third PCA axis explained only 2.2% of variation, it provided valuable information by distinguishing *V. edule* from the other taxa and delineating two distinct subclusters within the *V. sargentii* group (Appendix S5).

### Phylogenetic network analysis

**Figure 5:**
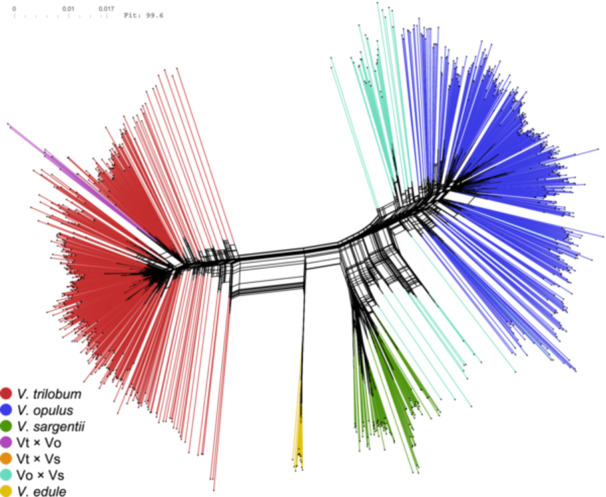
Splits network (neighbor‐net) of highbush cranberry (*Viburnum* spp.), based on complete single nucleotide polymorphism (SNP) data set. Branch tips are colored based on genetic identifications (cf. Figure 2; *K* = 4).

The splits network generated using the complete SNP data set (Figure 5) shows similar patterns to the structure and PCA analyses (Figures 2 and 4; Appendix S5). The major division of the network along the left‐right hemisphere matches patterns observed in prior analyses with the strongest statistical support: namely, the division of *V. trilobum* and *V. opulus* for *K* = 2 clusters (Figure 2), and the relative positions of the taxa from left to right along PCA axis 1 (Figure 4; Appendix S5). All four taxa are clearly separated into distinct edge bundles, or clades, though *V. opulus* and *V. sargentii* are clearly more closely related to each other than either is to *V. trilobum*. The highly networked areas connecting the Vo × Vs hybrid individuals with the *V. opulus* and *V. sargentii* clades indicate ambiguous phylogenetic relationships. The major incongruity between the splits network and previous analyses is in the placement of the Vt × Vo individuals and the single Vt × Vs hybrid individuals within the American cluster, which is not indicative of hybrid ancestry (Figure 5).

### Population structure within species

**Figure 6:**
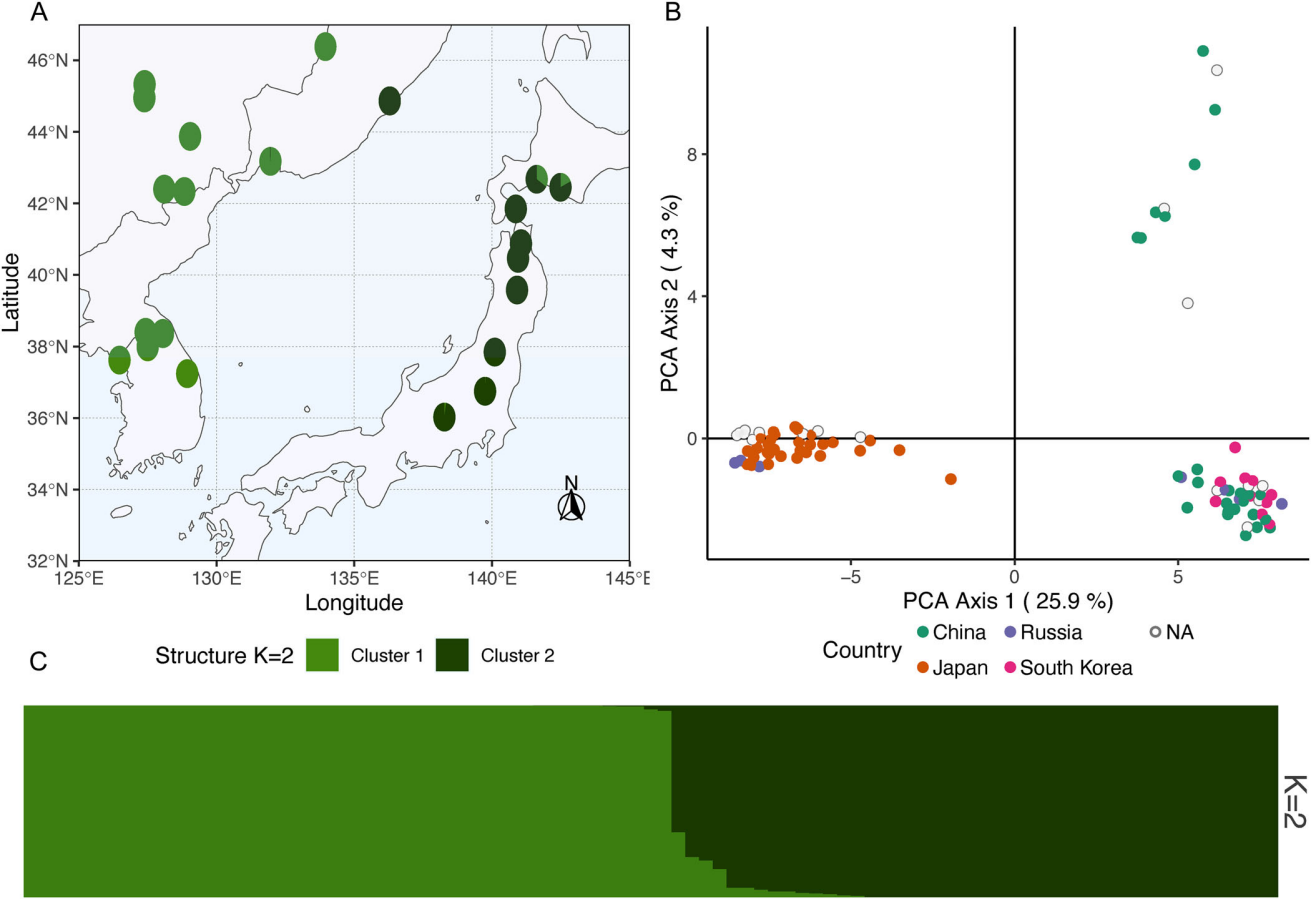
Evidence of genetic substructure within *Viburnum sargentii*. (A) Structure cluster assignments for *K* = 2 clusters plotted by location of origin. (B) Principal component analysis (PCA) of *V. sargentii* with points colored by country of origin. (C) Structure bar plots for *K* = 2 clusters, sorted by cluster membership.

Independent structure analysis of *V. sargentii* individuals revealed high Δ*K* support for *K* = 2 clusters corresponding to mainland Asia and Japan (Figure 6A; Appendix S6). Principal component analysis of *V. sargentii* with samples coded by country of origin reiterates this genetic division based on geography (Figure 6B). A nearly equal division of the data set into two clusters was observed, along with a small group of admixed individuals (Figure 6C). This division of *V. sargentii* was also apparent when examining the *K* = 5 clusters model in the complete analysis (Figure 2). The admixed individuals of known provenance originate primarily from two populations originating from the Japanese island of Hokkaido. The only notable exception to the geographic‐based clustering pattern was a population originating from Primorsky Krai, Russia (Figure 6A).

Independent structure analyses of *V. trilobum* and *V. opulus* showed the highest Δ*K* support for *K* = 2 clusters. In *V. trilobum*, cluster 1 is dominant to the western half of the range, while cluster 2 is more dominant on the eastern portion of the range. In contrast, *V. opulus* shows no clear geographic gradient observed between clusters 1 and 2. Samples with nearly complete membership in cluster 2 were exclusively from the Caucasus region (Appendix S7).

### Geographic context of results

**Figure 7:**
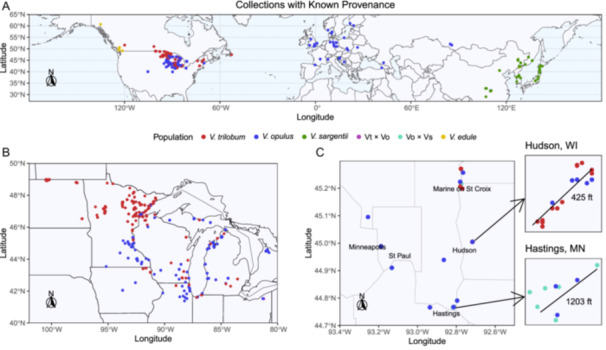
Geographic origin of highbush cranberry (*Viburnum* spp.) specimens of known provenance, with points colored by genetic identifications (cf. Figure 2; *K* = 4). Panels show sampling locations at multiple spatial scales: (A) globally, (B) across the Upper Midwest United States, and (C) in the vicinity of the Twin Cities metro area, with inset maps highlighting mixed populations located near Hudson, Wisconsin, and Hastings, Minnesota, USA.

With few exceptions, the native ranges of *V. opulus* and *V. sargentii* in Europe and Asia, respectively, remained consistent with their genetic structure. In contrast, the native range of *V. trilobum* in North America is now intermixed with naturalized populations of *V. opulus* and, to a lesser extent, Vo × Vs hybrids (Figure 7A).

Certain portions of the Upper Midwest, such as northern Minnesota and eastern North Dakota, USA, were found to still be dominated by *V. trilobum*. However, other areas, such as southeast Minnesota, northern Illinois, and Michigan, USA, and northwest Ontario, Canada, were more frequently inhabited by *V. opulus* (Figure 7B). Several areas were sampled containing sympatric populations of *V. trilobum* and *V. opulus*, yet no hybrid progeny were detected at these sites. One example located near Hudson, Wisconsin, USA, contained a mixture of mature *V. trilobum* and *V. opulus* plants, along with numerous seedlings (Figure 7C). Only six of the eight Vt × Vo hybrids identified in the study were from North America: one from northeast Minnesota, one from Michigan's Upper Peninsula, and four from northeast Ohio, USA. The remaining two Vt × Vo hybrids were collected from botanical gardens in Finland. The only Vt × Vs hybrid identified in the study was a nursery stock plant originally sourced from an unknown location in Wisconsin.

Additionally, the study identified several locations where Vo × Vs hybrids were present, even though no pure *V. sargentii* specimens were found naturalizing in North America. In nearly every instance, European specimens were found in proximity to these Vo × Vs specimens, as illustrated by a population located near Hastings, Minnesota (Figure 7C).

### Genetic diversity and *F*‐statistics

Observed heterozygosity (H_O_) was extremely low, ranging from 0.004 in *V. opulus* to 0.154 in the single Vt × Vs individual. Unsurprisingly, H_O_ was higher in the hybrid populations compared to non‐admixed species. Expected heterozygosity (H_E_) was similarly very low, with values ranging from 0.019 to 0.113. High positive values of the inbreeding coefficient (F_IS_) were observed for all populations, ranging from 0.345 to 0.650, suggesting that all populations are highly inbred. Lastly, allelic richness (Ar) was low overall, with a range from 1.020 to 1.154; like *H*_O_, it was higher in the hybrid populations than non‐admixed species (Table 1).

**Table 1:** Measures of genetic diversity by highbush cranberry (*Viburnum* spp.) population, grouped according to genetic identifications. Number of individuals (*n*) in each population, observed heterozygosity (*H*_O_), expected heterozygosity (*H*_E_), inbreeding coefficient (*F*_IS_), and allelic richness (Ar).

Population	*n*	*H*_O_	*H*_E_	*F*_IS_	Ar
*V. trilobum*	425	0.008	0.023	0.355	1.024
*V. opulus*	409	0.004	0.019	0.350	1.020
*V. sargentii*	91	0.020	0.081	0.650	1.084
Vt × Vo	8	0.080	0.113	0.345	1.125
Vt × Vs	1	0.154	0.077	–	1.154
Vo × Vs	45	0.039	0.085	0.500	1.087
*V. edule*	16	0.011	0.030	0.606	1.033

The population structure identified in PCA and structure analyses was strongly reiterated by pairwise measurements of genetic fixation (*G'*_ST_) and allelic differentiation (*D*_EST_; Table 2). *Viburnum trilobum* and *V. opulus* are extremely close to fixation (*G'*_ST_ = 0.949) and exhibit high allelic differentiation (*D*_EST_ = 0.442). *Viburnum sargentii* showed similar levels of fixation and differentiation when comparing both *V. trilobum* and *V. opulus*, though the latter comparison produced slightly lower values, indicating a closer relationship between *V. sargentii* and *V. opulus* (Table 2). As expected, the hybrid populations produced relatively lower *F*‐statistics for comparisons including one of their parent species (e.g., Vt × Vo hybrids had lower estimated fixation and differentiation estimates when compared to *V. trilobum* and *V. opulus*, as opposed to *V. sargentii*). The outgroup species, *V. edule*, had similar values of *G'*_ST_ and *D*_EST_ for all comparisons.

**Table 2:** Genetic fixation and allelic differentiation among highbush cranberry (*Viburnum* spp.) populations. The lower portion of the triangle contains unbiased *G′*_ST_ (Nei, 1987), while the upper portion contains *D*_EST_ (Jost, 2008).

*G′*_ST_/*D*_EST_	*V. trilobum*	*V. opulus*	*V. sargentii*	Vt × Vo	Vo × Vs	*V. edule*
*V. trilobum*		0.442	0.418	0.103	0.458	0.363
*V. opulus*	0.949		0.301	0.187	0.054	0.354
*V. sargentii*	0.875	0.838		0.347	0.165	0.333
Vt × Vo	0.543	0.690	0.742		0.222	0.320
Vo × Vs	0.885	0.478	0.610	0.631		0.334
*V. edule*	0.920	0.928	0.824	0.778	0.823	

The AMOVA showed that most of the genetic variation (88%) was found among populations with Phi_ST_ = 0.884 (*p* ≤ 0.0001), while within population variation accounted for only 12% of the total (Table 3).

**Table 3:** Analysis of molecular variance (AMOVA) for variance among and within highbush cranberry population partitions, degrees of freedom (df), sums of squares (SS), mean squares (MS), estimated variance (Est. var.), percent variation (%), population differentiation statistics (Phi), and *P* values.

**Partitioning**	**df**	**SS**	**MS**	**Est. var.**	**%**	**Phi**
Among populations	6	41191.72	6865.29	64.82	88%	Phi_ST_ = 0.884***
Within populations	988	8375.83	8.48	8.48	12%	
Total	994	49567.55	49.87	73.30	100%	

*** *P* ≤ 0.0001.

### Clonality

The genetic distance threshold required to group all biological replicates into identical MLLs was the same among all three algorithms (*t* = 0.0032). However, the total number of MLLs differed among algorithms from 546 (nearest neighbor) to 689 (farthest neighbor). For all populations except *V. opulus*, the number of MLLs was identical, or nearly so, among the three algorithms. In contrast, *V. opulus* exhibited a wide range of MLLs, from 5 (nearest neighbor) to 140 (farthest neighbor) (Table 4). This may partly be explained by an extremely right‐skewed distribution of pairwise genetic distances among samples, indicating a very close genetic relationship among many of the samples (Appendix S8).

**Table 4:** Number of multi‐locus lineages (MLLs) by highbush cranberry (*Viburnum* spp.) population for three clustering algorithms using a bitwise genetic distance threshold of *t* = 0.0032.

Population	Individuals (*n*)	MLLs (*n*) by clustering algorithm (*t* = 0.0032)		
		Farthest neighbor	Average neighbor	Nearest neighbor
*V. trilobum*	425	390	387	383
*V. opulus*	409	140	94	5
*V. sargentii*	91	89	89	88
Vt × Vo	8	8	8	8
Vt × Vs	1	1	1	1
Vo × Vs	45	45	45	45
*V. edule*	16	16	16	16
**Total**	995	689	640	546

## DISCUSSION

### Genetic structure and species relationships

This study is the most extensive analysis of highbush cranberry viburnum to date, and the only known genetic survey of *V. opulus* and *V. sargentii* naturalization in North America. Multiple lines of evidence indicate that these taxa are genetically distinct and that *V. opulus* and *V. sargentii* are sister taxa, with *V. trilobum* sister to the *opulus*–*sargentii* clade. In our primary structure analysis, the highest Δ*K* support was for two clusters (Appendix S3), which primarily differentiated *V. trilobum* from *V. opulus* (Figure 2). In this model, *V. sargentii* had a majority group membership in the *V. opulus* cluster, suggesting that the two populations share a greater proportion of genetic variation (Porras‐Hurtado et al., 2013). However, given the known limitations of structure in properly differentiating populations with small sample size (Porras‐Hurtado et al., 2013; Lawson et al., 2018), we also examined *K* values 3–5 for their ability to differentiate the smaller *V. sargentii* and *V. edule* groups. Ultimately, *K* = 4 clusters was the model deemed to be the most biologically meaningful, given that it effectively differentiated all four species, despite low Δ*K* support (Figure 2; Appendix S3). The pairwise structure analyses of *V. trilobum*, *V. opulus*, and *V. sargentii* provided added confidence to the delimitation of the genetic clusters and admixture zones (Appendix S4).

Unambiguous clustering of *V. trilobum*, *V. opulus*, *V. sargentii*, and *V. edule* was also observed in PCA. The majority of genetic variation (54.2%) was explained by PCA axis 1, along which the *V. opulus* and *V. sargentii* clusters are plotted closer to each other than to the *V. trilobum* cluster (Figure 4; Appendix S5). This proximity suggests a closer relationship between the Eurasian species (McVean, 2009). Pairwise comparisons among populations for genetic fixation (*G'*_ST_) and allelic differentiation (*D*_EST_) similarly indicate that all three species are strongly genetically differentiated, but that there is a closer relationship between *V. opulus* and *V. sargentii* compared with *V. trilobum* (Table 2). The consistently lower values of *D*_EST_ in relation to *G'*_ST_ may be explained by the low values of expected heterozygosity observed for these populations, which limits the maximum value of *D*_EST_ (Table 1; Meirmans and Hedrick, 2011). What is most important is that both *G'*_ST_ and *D*_EST_ describe similar relative patterns among the populations (Table 2). Finally, phylogenetic network analysis showed a primary separation of the highbush cranberry specimens into a *V. trilobum* clade and a *V. opulus*–*sargentii* clade, followed by a secondary division into *V. opulus* and *V. sargentii* clades (Figure 5). These observations are congruent with previously inferred phylogenies, which show a closer relationship between *V. opulus* and *V. sargentii* (Clement et al., 2014; Landis et al., 2021).

### Patterns of reproductive isolation and taxonomic implications

Our results reinforce the view that *V. trilobum*, *V. opulus*, and *V. sargentii* are, at least historically, separately evolving lineages that have become genetically differentiated because of their geographic isolation (Landis et al., 2021). However, given the recent human‐mediated introduction of *V. opulus* and *V. sargentii* into North America, future taxonomic revisions will need to consider whether these lineages are likely to continue evolving independently in the future (De Queiroz, 2007; Donoghue, 2022). This study provides a unique snapshot of the present reproductive isolation among these taxa within their newly sympatric ranges in North America. The finding that *V. trilobum* is largely reproductively isolated from *V. opulus* and *V. sargentii* in North America came as a surprise, especially given the success of intentional hybridization between *V. trilobum* and *V. opulus* (Figures 2 and 7B, C; Egolf, 1956). The detection of only six Vt × Vo hybrids and one Vt × Vs hybrid in North America strongly suggests the presence of an unknown reproductive barrier.

The apparent reproductive isolation of *V. trilobum* might be the result of a prezygotic barrier, such as a difference in flowering phenology. This mechanism has been proposed to maintain species boundaries in other sympatric *Viburnum* taxa, such as the *V. nudum* complex in eastern North America (Spriggs et al., 2019). Thus, future studies should examine whether *V. trilobum*, *V. opulus*, and *V. sargentii* exhibit differences in time of flowering, anthesis, or pollen receptivity that might enforce their genetic isolation. Differences in pollinator preference are unlikely given that all three taxa possess similar flower morphology and are visited by a similar range of pollinators (Waldbauer, 1984; Krannitz and Maun, 1991b; Englund, 1993; Wong Sato and Kato, 2021). The presence of a postzygotic barrier also seems unlikely. First, the production of viable seeds and offspring from crosses between *V. opulus* and *V. trilobum* suggests that seed abortion is not a strong postzygotic barrier, at least for F_1_ hybrids (Egolf, 1956; Kirkbride et al., 2015; Rebernig et al., 2015). A more likely possibility is that hybrid inviability, or a reduction in hybrid fitness due to incongruity, might be preventing the long‐term establishment and reproductive success of hybrid progeny (Mino et al., 2022). Finally, hybrid sterility or inability to backcross to a particular parent species could also be preventing F_1_ hybrids from reproducing (Yu et al., 2016; Wiens and Colella, 2025). However, this is likely not the case given that three of the Vt × Vo hybrids had unequal admixture proportions, identifying them as advanced generation hybrids (Vähä and Primmer, 2006; Bohling et al., 2013; Van Wyk et al., 2017). Thus, the apparent reproductive isolation of *V. trilobum* from sympatric populations of *V. opulus* and *V. sargentii* provides a strong argument for elevating the former taxon to species rank.

Assessing the reproductive isolation of *V. opulus* and *V. sargentii* is more difficult. Not only were more Vo × Vs hybrids observed (*n* = 45; Figure 3), but there was also a greater variety of advanced generation Vo × Vs hybrids (Figures 2, 4, and 5). This suggests that *V. opulus* and *V. sargentii* are less reproductively isolated from each other than either is from *V. trilobum*, consistent with their phylogenetic relationships (Clement et al., 2014; Landis et al., 2021). Thus, the genetic differentiation between *V. opulus* and *V. sargentii* may simply reflect local adaptation or genetic drift resulting from their historically allopatric ranges, rather than reproductive isolation (Bickford et al., 2007). However, it is important to note that only 12 of the Vo × Vs hybrid samples were from wild, naturalizing populations in North America (26.7%), while the remainder were from arboreta and botanical gardens (46.7%), nurseries (24.4%), or landscape settings (2.2%; Appendix S1). Of the 130 specimens originating from wild areas in Europe and Asia, only one showed evidence of admixture—a specimen collected in 1992 from the province Gangwon‐do, South Korea (accession: BERG: 12661; see Appendix S1). Based on these data, the native ranges of *V. opulus* and *V. sargentii* remain genetically and geographically isolated (Figure 7A), consistent with historical documentation (Hultén and Fries, 1986). However, this conclusion is based almost entirely on ex situ specimens, so increased field sampling across Europe and Asia may reveal a more complex genetic landscape. Crossing studies would also help determine whether these taxa are likely to remain on separate evolutionary trajectories in areas of sympatry.

### Conservation of *V. trilobum*

The low frequency of Vt × Vo hybrids observed in this study is promising for *V. trilobum* conservation and reduces concerns that the *V. trilobum* gene pool is being diluted through hybridization (Nellessen, 2006). However, given the widespread sympatric distributions of *V. trilobum* and *V. opulus*, it is almost certain that additional Vt × Vo hybrids exist in North America, particularly in areas that were underrepresented in this study, such as the eastern United States and Canada (Figure 7). At present, nothing is known about the fitness of these hybrids or their ability to backcross to parent populations and cause a gradual blending of the two species. Over a long period, the strength of these reproductive barriers will determine whether *V. trilobum* remains a separately evolving lineage despite the introduction of *V. opulus*, or if it will be slowly hybridized to extinction barring conservation intervention (De Queiroz, 2007; Donoghue, 2022). This phenomenon of genetic extinction through hybridization is a major risk facing plant species invaded by their exotic relatives and is well documented in plants (Rhymer and Simberloff, 1996; Wolf et al., 2001; Buggs, 2007; Prentis et al., 2007; Todesco et al., 2016). Furthermore, the limited hybridization between *V. trilobum* and *V. opulus* provides no insight into the comparative fitness of the parental species or their hybrids, and concerns about the displacement of *V. trilobum* by *V. opulus* remain a valid justification for conservation action (Nellessen, 2006). As a final complication, the invasive viburnum leaf beetle (*Pyrrhalta viburni* Paykull) from Eurasia has recently spread across North America (GBIF.org, 2025). This pest causes greater damage to North American *Viburnum* spp., including *V. trilobum*, than to Eurasian species such as *V. opulus* (Desurmont et al., 2011). This differential susceptibility may increase the risk of *V. trilobum* being displaced by *V. opulus*, compounding the conservation challenges already facing the native species.

Given the morphological similarity of these taxa (Figure 1) and the frequency of misidentifications (Figure 3), conservation efforts would benefit from the development of practical molecular identification tools. Our results show that 10% of *V. opulus* specimens were misidentified as *V. trilobum* (Figure 3). Misidentification of *V. sargentii* as *V. trilobum* was less common (3.3%; Figure 3) and may be of less concern, given that *V. sargentii* was not found to be naturalizing in North America (Figure 7). However, Vt × Vo hybrids were more frequently encountered in naturalized populations, and 15.6% of Vt × Vo hybrid specimens were misidentified as *V. trilobum*, further complicating conservation efforts (Figures 3 and 7). Methods of genetic identification would need to be accurate, affordable, and convenient enough to apply in a management context (Bruce et al., 2021). Polymerase chain reaction (PCR)‐based assays are currently popular for their accuracy, relative affordability, and ability to multiplex reactions (Patterson et al., 2017; Xia et al., 2018; Tripathi, 2023). Future research should therefore prioritize development of a PCR‐based diagnostic test for highbush cranberry to enable researchers, conservationists, and the nursery industry to overcome the limitations of morphological identification.

### Cryptic *V. sargentii* subtaxa

The genetic division of *V. sargentii* by geographic origin was not previously recognized on the basis of morphology (Figure 6; Hara, 1983; Qiner et al., 2011). These cryptic subtaxa likely arose due to the natural barrier to gene flow provided by the Sea of Japan (Hending, 2025). Careful examination of these *V. sargentii* subtaxa should be conducted to determine whether traits reported as inconsistent in *V. sargentii*, such as anther color (Dirr, 2007) and pubescence (Hara, 1983), are related to geographic population structure. Further investigation might also clarify whether any diagnostic features can be used to distinguish the *V. sargentii* subtaxa from each other, and from *V. opulus*. Recognition of the morphological and biochemical differences, ecological roles, and reproductive compatibility of the *V. sargentii* subtaxa could lead to their formal taxonomic recognition and integration in future biodiversity research (Fišer et al., 2018). In the meantime, native plant restoration efforts in Japan and mainland Asia should take care to utilize local ecotypes so that these taxa are conserved for future study. To facilitate further investigation, the structure *q*‐values for the “Japan” and “mainland” clusters, along with accession numbers, are provided in Appendix S9.

### Genetic diversity, demography, and reproductive biology

All highbush cranberry species (*V. trilobum*, *V. opulus*, *V. sargentii*) display a low level of intraspecific genetic diversity, as exhibited by their low heterozygosity (*H*_o_ and *H*_E_), low allelic richness (Ar), and high inbreeding coefficients (*F*_IS_; Table 1). Overall, AMOVA results showed that most of the genetic variation in our study was partitioned among populations (88%) rather than within populations (12%; Table 3). From a conservation standpoint, the low diversity present in *V. trilobum*, *V. opulus*, and *V. sargentii* may not necessarily imply the reduced fitness of these populations, although it is likely to negatively impact these species’ adaptive potential and resilience to changing environmental conditions in the future (Bouzat, 2010; Olazcuaga et al., 2023). These data also raise questions about the demographic and reproductive factors that caused these populations to become so genetically differentiated that they are nearing fixation, while at the same time losing nearly all their within‐population genetic variation (Tables 1, 2, 3). One possibility is that these populations experienced a historical bottleneck that reduced their genetic variation and amplified the effects of genetic drift among survivors (Chakraborty and Nei, 1977; Bouzat, 2010; Olazcuaga et al., 2023). Prior research suggests that the *V. trilobum*–*opulus*–*sargentii* clade experienced a ~ 3.5‐fold reduction in ancestral population size, which may have facilitated the fixation of sterile marginal flowers in these taxa (Figure 1D–F; Park and Donoghue, 2021). Our DArTseq data provide another opportunity to test for such bottlenecks and assess whether their timing coincides with major climatic shifts, such as the last glacial maximum (Hewitt, 1996; Ony et al., 2021; Jin et al., 2024). Similarly, the especially low heterozygosity observed in *V. opulus* may reflect a founder effect, as most samples were collected from naturalized populations in North America (Figure 7; Barton and Charlesworth, 1984).

The low genetic diversity observed within *V. trilobum*, *V. opulus*, and *V. sargentii* might also be reinforced by their reproductive biology. For example, a high rate of self‐pollination would be expected to lead to a reduction in genetic diversity over time (Ingvarsson, 2002; Wright et al., 2013). However, given that these shrubs are primarily insect pollinated, self‐pollination is expected to be rare (Waldbauer, 1984; Kollmann and Grubb, 2002; Wong Sato and Kato, 2021). Experiments involving self‐pollination and pollinator exclusion have resulted in very low fruit set, reinforcing the idea that these plants are predominantly outcrossing and self‐incompatible (Egolf, 1956; Krannitz and Maun, 1991a). A possible explanation is that these species might experience high levels of geitonogamy, or pollen transfer among clonal ramets leading to self‐fertilization (Vallejo‐Marín et al., 2010). Our data support low levels of clonal reproduction in highbush cranberry, which would provide opportunities for geitonogamy to occur (Table 4). Furthermore, fruit abortion rates of ~90% have been observed in *V. opulus* (Englund, 1994), a phenomenon commonly associated with geitonogamy (Bocanegra‐González et al., 2025). For self‐incompatible species, geitonogamy is reported to cause reductions in fruit set and population genetic diversity (Lozada‐Gobilard et al., 2021; Matallana‐Puerto et al., 2024; Bocanegra‐González et al., 2025). Thus, geitonogamy serves as a plausible reproductive mechanism driving reduced genetic diversity in highbush cranberry species despite being insect pollinated.

It has previously been noted that *V. opulus* is capable of widespread clonal propagation via layering (Kollmann and Grubb, 2002). We have also observed layering within *V. trilobum* colonies in Minnesota (D. Tork, personal observation). The clonality estimates presented herein support these observations and suggest that some level of clonal reproduction is present in all three species, but especially in *V. opulus*. However, it is difficult to precisely estimate the number of clonal lineages because of the large differences observed among the MLL clustering algorithms (Table 4). This discrepancy among algorithms may be explained by the strongly right‐skewed distribution of pairwise genetic distances among samples, which shows that many sample pairs in our analysis are separated by very small genetic distances (Appendix S8). Consequently, the total number of MLLs is expected to collapse rapidly across all algorithms as the distance threshold is increased and will vary based on how conservatively each algorithm groups MLLs (Appendix S8; Kamvar et al., 2015). This observation largely explains the wide range of estimated MLLs observed among algorithms for *V. opulus*, despite using a very small genetic distance threshold. Regardless of the precise number of MLLs present, the comparatively high estimates of clonality in *V. opulus* align with the observation that it has the lowest heterozygosity and allelic richness among all the populations studied (Table 1). High levels of clonality have been associated with low levels of genetic diversity in other plant species (Lozada‐Gobilard et al., 2021). The observed clonality in *V. trilobum*, *V. opulus*, and *V. sargentii* might therefore contribute to their low genetic diversity, possibly through the reinforcement of geitonogamy. Clonality is common in invasive plants and has been shown to confer fitness advantages, such as enhanced competitive ability and colonization success (Song et al., 2013; Wang et al., 2019). The greater incidence of clonality observed in *V. opulus* might help explain its tendency to outcompete *V. trilobum* (Nellessen, 2006). However, over evolutionary timescales, high levels of clonality may also increase the risk of accumulating deleterious mutations through Muller's ratchet, potentially reducing long‐term adaptive potential (Haigh, 1978).

### Evolutionary stasis and phenotypic differentiation

The morphological stability of these taxa (Figure 1) despite strong genetic divergence (Figures 2 and 4; Table 2) is consistent with the phenomenon of evolutionary stasis, wherein little to no morphological change occurs during millions of years of evolutionary history (Eldredge et al., 2005; Struck and Cerca, 2022). This stability may reflect these species’ lack of genetic diversity (Table 1), perhaps a result of historical bottlenecks or their tendency for asexual propagation (Table 4; Park and Donoghue, 2021; Struck and Cerca, 2022). Despite their apparent similarities, a careful reexamination of *V. trilobum*, *V. opulus*, and *V. sargentii* morphology is warranted, given that misidentifications may have confounded earlier characterizations (Figure 3). Such efforts could leverage the genetically verified accessions from living collections and herbaria (Appendix S1). Direct comparisons of the three taxa are conspicuously lacking in the literature, and those that exist originate from a single research group (see, e.g., Česonienė et al., 2012; Kraujalytė et al., 2013; Paulauskas et al., 2015). Moreover, most of the research on the horticultural, biochemical, and pharmacological potential of highbush cranberry has exclusively considered *V. opulus* (Kajszczak et al., 2020; Karatoprak and İlgün, 2022; Yaman, 2022; Gok, 2023). The finding that most of the genetic variation was partitioned among species (Table 3) suggests that there might be underexplored phenotypic variation that is worthy of further investigation.

## CONCLUSIONS

Taxonomic treatments lumping *V. trilobum*, *V. opulus*, and *V. sargentii* as varieties or subspecies of *V. opulus* were probably inevitable given their morphological similarity. However, our results demonstrate that these cryptic species are genetically distinct, and that *V. opulus* and *V. sargentii* are more closely related to each other than to *V. trilobum*. The continued genetic isolation of *V. trilobum*, despite widespread sympatry with its Eurasian congeners in North America, suggests that it should attain the rank of species. In contrast, the choice to lump or split *V. opulus* and *V. sargentii* remains less clear due to their more frequent hybridization. One limitation of our study is that many of the Eurasian accessions originated from ex situ collections, and it was in these common garden settings that many of the Vo × Vs hybrids were observed. Thus, an analogous study based on wild‐collected germplasm is needed to determine whether *V. opulus* and *V. sargentii* are likely to remain on separate evolutionary trajectories within their native ranges. Finally, our data indicate that all three taxa exhibit low genetic diversity and partial clonal reproduction, raising further questions about their reproductive biology and long‐term evolutionary potential.

These findings have direct implications for the conservation of American highbush cranberry (*V. trilobum*). The limited hybridization observed between *V. opulus* or *V. sargentii* and *V. trilobum* is promising in the short term, but it should not be ruled out as an evolutionary risk until more is known about the mechanisms regulating hybrid formation. Furthermore, the widespread naturalization of *V. opulus* and, to a lesser extent, Vo × Vs hybrids, continues to pose a threat to *V. trilobum* through competition for habitat and resources. Regions where *V. opulus* is still rare or absent (e.g., northern Minnesota) deserve greater attention for *V. trilobum* seed conservation efforts. These areas would also be ideal locations for active management and exclusion of *V. opulus*, in order to prevent further hybrid formation. At the same time, the limited genetic diversity observed in *V. trilobum* highlights the need for seed collection efforts across its broader range—particularly in eastern North America—to ensure that remaining genetic variation is adequately represented in ex situ collections.

Efforts to conserve *V. trilobum* will require improved tools for species identification. Morphological similarity between *V. trilobum* and *V. opulus* remains a major challenge, as illustrated by the frequent misidentifications reported in this study. To address this, it will be necessary to identify more reliable diagnostic traits or develop practical tools for molecular identification, such as a PCR‐based assay. Such tools could be applied in the nursery and landscape industry to verify stock materials. They could also be used by researchers to better understand the distribution of *V. opulus* in North America, particularly in areas that were not well represented in this study, such as the eastern United States and the Canadian provinces. The development and application of these conservation tools will be critical to the long‐term preservation of American highbush cranberry (*V. trilobum*), an ecologically and culturally important species in North America.

## AUTHOR CONTRIBUTIONS

N.O.A., A.G.S., A.B.: conceptualization. D.G.T., N.O.A., A.G.S.: data curation, funding acquisition. D.G.T.: formal analysis. D.G.T., A.G.S.: visualization, investigation, supervision, project administration. D.G.T.: writing—original draft. D.G.T., N.O.A., A.G.S., A.B.: writing—review, editing.

## CONFLICT OF INTEREST STATEMENT

The authors declare that they have no conflicts of interests.

## Supporting information


**Appendix S1.** List of highbush cranberry (*Viburnum* spp.) specimens included in this study by unique sample ID, species ID reported at the time of collection, species ID based on genetic analysis, plant setting (wild, landscape, arboretum or botanic garden, herbarium), whether the provenance of the material is known or unknown, institution code (see footer) and accession number (if applicable), and cultivar, variety, or form name (if applicable). Biological replicates are indicated by “_1” at the end of the sample ID. The presence of “NA” in the genetic ID column indicates that the sample failed DNA extraction or downstream QC checks and was thus excluded from the genetic analysis.

**Appendix S2.** Principal component analysis (PCA) with reported highbush cranberry (*Viburnum* spp.) identifications based on visual field identification and/or database records.

**Appendix S3.** ΔK support for number of clusters (K) for STRUCTURE analysis of all highbush cranberry (*Viburnum* spp.) specimens.

**Appendix S4.** Separate structure analyses evaluating pairwise groupings of three main genetically identified species groups *Viburnum trilobum*, *V. opulus*, *V. sargentii*. Statistical support (Δ*K*) for the number of clusters is shown in A–C, while the membership proportions of all individuals for *K* = 2 clusters is shown in D–F. Admixed zones are defined by dashed vertical lines. The analysis of *V. trilobum*, *V. opulus* and their hybrids (Vt × Vo) is shown in panels A, D; *V. opulus*, *V. sargentii* and their hybrids (Vo × Vs) are shown in panels B, E; *V. trilobum*, *V. sargentii*, and their hybrids (Vt × Vs) are shown in panels C, F.

**Appendix S5.** Principal component analysis (PCA) axes 1 and 3 for 995 highbush cranberry specimens, with populations assigned based on genetic identifications from structure using *K* = 4 clusters. Hybrid groups include *V. trilobum × V. opulus* (Vt × Vo), *V. trilobum* × *V. sargentii* (Vt × Vs), and *V. opulus* × *V. sargentii* (Vo × Vs).

**Appendix S6.** ΔK support for number of clusters (K) for STRUCTURE analysis of *Viburnum* sargentii.

**Appendix S7.** Analysis of substructure in *Viburnum trilobum* and *V. opulus*; (A) Δ*K* support for number of clusters (*K*) in *V. trilobum*; (B) average structure cluster assignments for each *V. trilobum* population plotted by location; (C) Δ*K* support for number of clusters (*K*) in *V. opulus*; (D) average structure cluster assignments for each *V. opulus* population plotted by location.

**Appendix S8.** Histogram showing bitwise distance distribution for pairwise comparisons among all highbush cranberry samples. The number of estimated multilocus lineages for nearest neighbor (green), average neighbor (UPGMA or Unweighted Pair Group Method with Arithmetic Mean; blue), and farthest neighbor (red) are shown for various genetic distance thresholds.

**Appendix S9.** List of *Viburnum sargentii* specimens including unique sample ID, whether the provenance is known or unknown, institution code (see footer) and accession number, and structure q‐values indicating each specimen's estimated proportion of membership in the mainland (Cluster 1) and Japan (Cluster 2) genetic clusters.

## Data Availability

The genomic data sets and R code from this study are archived and available through Data Repository for the University of Minnesota (DRUM): https://doi.org/10.13020/r0mc-cq97.
