# Supplementary material for: American highbush cranberry maintains strong population structure despite naturalization of Eurasian relatives in North America
Source: Am J Bot. 2025 Nov 14;112(11):e70124. doi: 10.1002/ajb2.70124 (PMC12640478; doi:10.1002/ajb2.70124)
Supplement: Supplementary file 1 — Appendix S1. List of highbush cranberry (Viburnum spp.) specimens included in this study by unique sample ID, species ID reported at the time of collection, species ID based on genetic analysis, plant setting (wild, landscape, arboretum or botanic garden, herbarium), whether the provenance of the material is known or unknown, institution code (see footer) and accession number (if applicable), and cultivar, variety, or form name (if applicable). Biological replicates are indicated by “_1” at the end of the sample ID. The presence of “NA” in the genetic ID column indicates that the sample failed DNA extraction or downstream QC checks and was thus excluded from the genetic analysis. [file AJB2-112-e70124-s008.docx]

**Appendix S1.** List of highbush cranberry (*Viburnum* spp.) specimens included in this study by unique sample ID, species ID reported at the time of collection, species ID based on genetic analysis, plant setting (wild, landscape, arboretum or botanic garden, herbarium), whether the provenance of the material is known or unknown, institution code (see footer) and accession number (if applicable), and cultivar, variety, or form name (if applicable). The presence of “_1” at the end of a sample ID indicates a biological replicate. The presence of “NA” in the genetic ID column indicates that the sample failed DNA extraction or downstream QC checks and was thus not included in the genetic analysis.

| **Sample ID** | **Reported ID** | **Genetic ID** | **Setting** | **Prov.** | **Inst. code: accession no.** | **Cultivar, variety, or form name** |
| --- | --- | --- | --- | --- | --- | --- |
| AH002 | *V. opulus* | *V. trilobum* | Wild | Known |  |  |
| JF039 | *V. opulus* | *V. opulus* | Wild | Known |  |  |
| JF027 | *V. opulus* | Vt × Vo | Landscape | Unknown |  |  |
| JF006 | *V. trilobum* | *V. trilobum* | Wild | Known |  |  |
| JF008 | Unknown | *V. trilobum* | Wild | Known |  |  |
| JF001 | *V. opulus* | *V. trilobum* | Wild | Known |  |  |
| JF001_1 | *V. opulus* | *V. trilobum* | Wild | Known |  |  |
| JF002 | Unknown | *V. trilobum* | Wild | Known |  |  |
| JF003 | Unknown | *V. opulus* | Wild | Known |  |  |
| JF004 | *V. opulus* | *V. opulus* | Landscape | Unknown |  |  |
| JF005 | *V. opulus* | *V. opulus* | Wild | Unknown |  |  |
| JF007 | Unknown | *V. trilobum* | Wild | Known |  |  |
| JF009 | Unknown | *V. trilobum* | Landscape | Unknown |  |  |
| JF010 | *V. opulus* | *V. opulus* | Landscape | Unknown |  |  |
| JF011 | Unknown | *V. trilobum* | Landscape | Unknown |  |  |
| JF012 | Unknown | *V. trilobum* | Wild | Known |  |  |
| JF013 | Unknown | *V. trilobum* | Wild | Known |  |  |
| JF014 | Unknown | *V. trilobum* | Wild | Known |  |  |
| JF015 | *V. opulus* | *V. opulus* | Wild | Known |  |  |
| JF016 | Unknown | *V. opulus* | Wild | Known |  |  |
| JF017 | *V. opulus* | *V. opulus* | Landscape | Unknown |  |  |
| JF018 | *V. opulus* | *V. opulus* | Wild | Known |  |  |
| JF019 | *V. opulus* | *V. opulus* | Wild | Known |  |  |
| JF020 | *V. opulus* | *V. opulus* | Landscape | Unknown |  |  |
| JF021 | *V. opulus* | *V. opulus* | Wild | Known |  |  |
| JF022 | Unknown | *V. trilobum* | Wild | Known |  |  |
| JF023 | *V. opulus* | *V. opulus* | Wild | Known |  |  |
| JF024 | Unknown | *V. trilobum* | Wild | Known |  |  |
| JF025 | Unknown | *V. trilobum* | Wild | Known |  |  |
| JF026 | *V. trilobum* | *V. trilobum* | Wild | Known |  |  |
| JF028 | Unknown | *V. trilobum* | Landscape | Unknown |  |  |
| JF029 | Unknown | *V. trilobum* | Wild | Known |  |  |
| JF030 | Unknown | *V. opulus* | Landscape | Unknown |  |  |
| JF031 | Unknown | *V. opulus* | Landscape | Unknown |  |  |
| JF032 | Unknown | *V. trilobum* | Wild | Known |  |  |
| JF033 | Unknown | *V. trilobum* | Wild | Known |  |  |
| JF034 | Unknown | *V. trilobum* | Wild | Known |  |  |
| JF035 | Unknown | *V. trilobum* | Wild | Known |  |  |
| JF036 | Unknown | *V. trilobum* | Wild | Known |  |  |
| JF037 | Unknown | *V. trilobum* | Wild | Known |  |  |
| JF038 | Unknown | *V. trilobum* | Wild | Known |  |  |
| JF040 | Unknown | *V. trilobum* | Wild | Known |  |  |
| JF041 | Unknown | *V. trilobum* | Wild | Known |  |  |
| JF042 | Unknown | *V. trilobum* | Wild | Known |  |  |
| JF043 | Unknown | *V. trilobum* | Wild | Known |  |  |
| JF044 | Unknown | *V. trilobum* | Landscape | Unknown |  |  |
| TK001 | Unknown | *V. trilobum* | Wild | Known |  |  |
| TK002 | Unknown | *V. trilobum* | Wild | Known |  |  |
| TK003 | Unknown | *V. trilobum* | Wild | Known |  |  |
| TK004 | Unknown | *V. trilobum* | Wild | Known |  |  |
| TK005 | Unknown | *V. trilobum* | Wild | Known |  |  |
| TK006 | Unknown | *V. trilobum* | Wild | Known |  |  |
| TK007 | Unknown | *V. trilobum* | Wild | Known |  |  |
| MSa001 | Unknown | *V. trilobum* | Wild | Known |  |  |
| MSa002 | *V. trilobum* | *V. trilobum* | Wild | Known |  |  |
| AH001 | Unknown | *V. trilobum* | Wild | Known |  |  |
| AH003 | *V. opulus* | *V. opulus* | Wild | Known |  |  |
| MSa003 | Unknown | NA | Wild | Known |  |  |
| MS001 | *V. opulus* | *V. opulus* | Arb./Bot. Gar. | Unknown | AMU: G_G018_003_0000_8XXX_4212 |  |
| MS002 | *V. opulus* | *V. opulus* | Arb./Bot. Gar. | Unknown | AMU: G_G018_001_0000_8XXX_4367 |  |
| MS003 | *V. trilobum* | *V. trilobum* | Arb./Bot. Gar. | Unknown | AMU: G_G015_001_0000_8XXX_2227 |  |
| MS004 | *V. sargentii* | Vo × Vs | Arb./Bot. Gar. | Unknown | AMU: D_D025_002_0000_8992_4992 |  |
| MS005 | *V. sargentii* | Vo × Vs | Arb./Bot. Gar. | Unknown | AMU: D_D025_002_0000_8992_4992 |  |
| JO001 | *V. opulus* | *V. opulus* | Arb./Bot. Gar. | Unknown | WESPE: 05635 | Aureum |
| JO002 | *V. opulus* | *V. opulus* | Arb./Bot. Gar. | Unknown | WESPE: 92651 | Notcutt's Variety |
| JO003 | *V. opulus* | *V. opulus* | Arb./Bot. Gar. | Unknown | WESPE: 82221 | Xanthocarpum |
| JO004 | *V. sargentii* | Vo × Vs | Arb./Bot. Gar. | Unknown | WESPE: 89264 | Onondaga |
| JO005 | *V. trilobum* | *V. sargentii* | Arb./Bot. Gar. | Unknown | WESPE: 88503 |  |
| TD001 | *V. opulus* | *V. opulus* | Arb./Bot. Gar. | Known | WOJ: No.1 |  |
| TD002 | *V. trilobum* | *V. opulus* | Arb./Bot. Gar. | Unknown | WOJ: No.2 |  |
| TD003 | *V. trilobum* | *V. opulus* | Arb./Bot. Gar. | Unknown | WOJ: No.3 |  |
| TD004 | *V. sargentii* | Vo × Vs | Arb./Bot. Gar. | Unknown | WOJ: No.4 |  |
| DT035 | *V. opulus* | *V. opulus* | Nursery | Unknown |  | Nanum |
| DT036 | *V. trilobum* | *V. opulus* | Nursery | Unknown |  | Wentworth |
| DT037 | *V. trilobum* | *V. trilobum* | Nursery | Unknown |  | Redwing |
| DT038 | *V. opulus* | *V. opulus* | Nursery | Unknown |  | Roseum |
| DT039 | *V. trilobum* | *V. trilobum* | Nursery | Unknown |  | Redwing |
| DT040 | *V. trilobum* | *V. opulus* | Nursery | Unknown |  | Wentworth |
| DT042 | *V. trilobum* | *V. opulus* | Nursery | Unknown |  |  |
| DT043 | *V. trilobum* | *V. opulus* | Nursery | Unknown |  |  |
| DT044 | *V. trilobum* | *V. opulus* | Nursery | Unknown |  |  |
| DT045 | *V. trilobum* | *V. opulus* | Nursery | Unknown |  |  |
| DT046 | *V. trilobum* | *V. trilobum* | Nursery | Unknown |  | Alfredo |
| DT047 | *V. trilobum* | *V. opulus* | Nursery | Unknown |  | Jewell Box |
| DT048 | *V. opulus* | *V. opulus* | Nursery | Unknown |  | Compactum |
| DT049 | *V. opulus* | *V. opulus* | Nursery | Unknown |  | Nanum |
| DT051 | *V. opulus* | *V. opulus* | Nursery | Unknown |  | Compactum |
| DT052 | *V. trilobum* | *V. trilobum* | Nursery | Unknown |  | Alfredo |
| DT053 | *V. trilobum* | *V. trilobum* | Nursery | Unknown |  | Bailey Compact |
| DT054 | *V. sargentii* | Vo × Vs | Nursery | Unknown |  | Onondaga |
| DT041 | *V. trilobum* | NA | Nursery | Unknown |  |  |
| DT050 | *V. opulus* | NA | Nursery | Unknown |  | Roseum |
| KA001 | *V. opulus* | *V. opulus* | Wild | Unknown |  |  |
| KA002 | *V. opulus* | *V. opulus* | Landscape | Unknown |  |  |
| KA004 | *V. opulus* | *V. opulus* | Arb./Bot. Gar. | Unknown | BBG: B917-029 | Compactum |
| KA005 | *V. opulus* | *V. opulus* | Arb./Bot. Gar. | Unknown | BBG: BD741-202 |  |
| DKh001 | *V. opulus* | *V. opulus* | Arb./Bot. Gar. | Known | BATU: DKh001 |  |
| TW001 | *V. trilobum* | *V. opulus* | Wild | Known |  |  |
| NW001 | *V. sargentii* | Vo × Vs | Arb./Bot. Gar. | Known | BERG: 12661 |  |
| DSS003 | *V. opulus* | *V. opulus* | Wild | Known |  |  |
| DSS001 | *V. opulus* | *V. opulus* | Wild | Known |  |  |
| DSS002 | *V. opulus* | *V. opulus* | Wild | Known |  |  |
| DSS004 | *V. trilobum* | *V. trilobum* | Arb./Bot. Gar. | Unknown | CBG: 1497-2014 | Spring Red Compact |
| JG001 | *V. opulus* | *V. opulus* | Arb./Bot. Gar. | Known | CBG: 371-1970 |  |
| JG002 | *V. sargentii* | Vo × Vs | Arb./Bot. Gar. | Unknown | CBG: 1987-2005 | Onondaga |
| JG003 | *V. trilobum* | *V. trilobum* | Arb./Bot. Gar. | Unknown | CBG: 1245-2009 |  |
| JG004 | *V. opulus* | *V. opulus* | Arb./Bot. Gar. | Unknown | CBG: 542-1989 | Nanum Bullatum |
| JG005 | *V. sargentii* | *V. opulus* | Arb./Bot. Gar. | Unknown | CBG: 533-2010 | Chiquita |
| JG006 | *V. trilobum* | *V. trilobum* | Arb./Bot. Gar. | Unknown | CBG: 316-2004 | Redwing |
| JG007 | *V. trilobum* | *V. trilobum* | Arb./Bot. Gar. | Unknown | CBG: 1703-2005 |  |
| JG008 | *V. trilobum* | *V. trilobum* | Arb./Bot. Gar. | Unknown | CBG: 539-1997 | Alfredo |
| MZ002 | *V. trilobum* | *V. trilobum* | Landscape | Unknown |  |  |
| PH009 | *V. trilobum* | *V. trilobum* | Wild | Known |  |  |
| PH001 | *V. trilobum* | *V. trilobum* | Wild | Known |  |  |
| PH002 | *V. trilobum* | *V. trilobum* | Wild | Known |  |  |
| PH003 | *V. trilobum* | *V. trilobum* | Wild | Known |  |  |
| PH004 | *V. trilobum* | *V. trilobum* | Wild | Known |  |  |
| PH005 | *V. trilobum* | *V. trilobum* | Wild | Known |  |  |
| PH006 | *V. trilobum* | *V. trilobum* | Wild | Known |  |  |
| PH007 | *V. trilobum* | *V. trilobum* | Wild | Known |  |  |
| PH008 | *V. trilobum* | *V. trilobum* | Wild | Known |  |  |
| PH010 | *V. trilobum* | *V. trilobum* | Wild | Known |  |  |
| MZ001 | *V. trilobum* | *V. trilobum* | Landscape | Unknown |  |  |
| MZ003 | *V. trilobum* | *V. trilobum* | Landscape | Unknown |  |  |
| MZ004 | *V. trilobum* | *V. trilobum* | Landscape | Unknown |  |  |
| PV002 | *V. trilobum* | *V. opulus* | Wild | Known |  |  |
| BM001 | *V. opulus* | *V. opulus* | Wild | Known |  |  |
| BM002 | *V. trilobum* | *V. trilobum* | Wild | Known |  |  |
| BM003 | *V. opulus* | *V. opulus* | Wild | Known |  |  |
| BM004 | *V. opulus* | *V. opulus* | Wild | Known |  |  |
| BM005 | *V. opulus* | *V. opulus* | Wild | Known |  |  |
| BM005_1 | *V. opulus* | *V. opulus* | Wild | Known |  |  |
| BM006 | *V. opulus* | *V. opulus* | Wild | Known |  |  |
| BM007 | *V. opulus* | *V. opulus* | Wild | Known |  |  |
| BM008 | *V. opulus* | *V. opulus* | Wild | Known |  |  |
| BM009 | *V. opulus* | *V. opulus* | Wild | Known |  |  |
| BM010 | *V. opulus* | *V. opulus* | Wild | Known |  |  |
| BM011 | *V. opulus* | *V. opulus* | Wild | Known |  |  |
| BM012 | *V. opulus* | *V. opulus* | Wild | Known |  |  |
| BM013 | *V. opulus* | *V. opulus* | Wild | Known |  |  |
| BM014 | *V. opulus* | *V. opulus* | Landscape | Unknown |  |  |
| BM015 | *V. opulus* | *V. opulus* | Wild | Known |  |  |
| BM016 | *V. opulus* | *V. opulus* | Wild | Known |  |  |
| BM017 | *V. opulus* | *V. opulus* | Wild | Known |  |  |
| BM018 | *V. opulus* | *V. opulus* | Wild | Known |  |  |
| BM019 | *V. opulus* | *V. opulus* | Wild | Known |  |  |
| BM020 | *V. opulus* | *V. opulus* | Wild | Known |  |  |
| BM021 | *V. opulus* | *V. opulus* | Wild | Known |  |  |
| JV001 | Unknown | NA | Wild | Known |  |  |
| PV001 | *V. trilobum* | NA | Wild | Known |  |  |
| MT001 | *V. opulus* | NA | Wild | Known |  |  |
| RD005 | *V. trilobum* | *V. trilobum* | Wild | Known |  |  |
| ME002 | Unknown | *V. trilobum* | Wild | Known |  |  |
| OB001 | Unknown | *V. trilobum* | Wild | Known |  |  |
| OB002 | Unknown | *V. trilobum* | Wild | Known |  |  |
| OB003 | Unknown | *V. trilobum* | Wild | Known |  |  |
| OB004 | Unknown | *V. trilobum* | Wild | Known |  |  |
| OB005 | Unknown | *V. trilobum* | Wild | Known |  |  |
| OB006 | Unknown | *V. trilobum* | Wild | Known |  |  |
| OB007 | Unknown | *V. trilobum* | Wild | Known |  |  |
| OB008 | Unknown | *V. trilobum* | Wild | Known |  |  |
| OB009 | Unknown | *V. trilobum* | Wild | Known |  |  |
| OB010 | Unknown | *V. trilobum* | Wild | Known |  |  |
| OB011 | Unknown | *V. trilobum* | Wild | Known |  |  |
| OB012 | Unknown | *V. trilobum* | Wild | Known |  |  |
| OB013 | Unknown | *V. trilobum* | Wild | Known |  |  |
| OB014 | Unknown | *V. trilobum* | Wild | Known |  |  |
| OB015 | Unknown | *V. trilobum* | Wild | Known |  |  |
| OB016 | Unknown | *V. trilobum* | Wild | Known |  |  |
| OB017 | Unknown | *V. trilobum* | Wild | Known |  |  |
| OB018 | Unknown | *V. trilobum* | Wild | Known |  |  |
| OB019 | Unknown | *V. trilobum* | Wild | Known |  |  |
| OB020 | Unknown | *V. trilobum* | Wild | Known |  |  |
| OB021 | Unknown | *V. trilobum* | Wild | Known |  |  |
| OB022 | Unknown | *V. trilobum* | Wild | Known |  |  |
| OB023 | Unknown | *V. trilobum* | Wild | Known |  |  |
| OB024 | Unknown | *V. trilobum* | Wild | Known |  |  |
| OB025 | Unknown | *V. trilobum* | Wild | Known |  |  |
| RD001 | *V. trilobum* | *V. trilobum* | Wild | Known |  |  |
| RD002 | *V. trilobum* | *V. trilobum* | Wild | Known |  |  |
| RD003 | *V. trilobum* | *V. trilobum* | Wild | Known |  |  |
| RD004 | *V. trilobum* | *V. trilobum* | Wild | Known |  |  |
| ME001 | Unknown | *V. trilobum* | Wild | Known |  |  |
| ME003 | Unknown | *V. trilobum* | Wild | Known |  |  |
| GB001 | *V. trilobum* | *V. trilobum* | Wild | Known |  |  |
| GB002 | *V. trilobum* | *V. trilobum* | Wild | Known |  |  |
| GB003 | *V. trilobum* | *V. trilobum* | Wild | Known |  |  |
| GB004 | *V. trilobum* | *V. trilobum* | Wild | Known |  |  |
| GB005 | *V. trilobum* | *V. trilobum* | Wild | Known |  |  |
| GB006 | *V. trilobum* | *V. trilobum* | Wild | Known |  |  |
| JMi001 | Unknown | *V. opulus* | Wild | Known |  |  |
| JMi002 | Unknown | *V. opulus* | Wild | Known |  |  |
| JMi003 | Unknown | *V. opulus* | Wild | Known |  |  |
| EB001 | Unknown | *V. trilobum* | Wild | Known |  |  |
| EB002 | Unknown | *V. trilobum* | Wild | Known |  |  |
| EB003 | Unknown | *V. trilobum* | Wild | Known |  |  |
| EB004 | Unknown | *V. trilobum* | Wild | Known |  |  |
| EB005 | Unknown | *V. trilobum* | Wild | Known |  |  |
| EB006 | Unknown | *V. trilobum* | Wild | Known |  |  |
| EB007 | Unknown | *V. trilobum* | Wild | Known |  |  |
| EB008 | Unknown | *V. trilobum* | Wild | Known |  |  |
| EB009 | Unknown | *V. trilobum* | Wild | Known |  |  |
| EB010 | Unknown | *V. trilobum* | Wild | Known |  |  |
| CV001 | *V. trilobum* | *V. trilobum* | Nursery | Unknown |  | Hahs |
| CV002 | *V. trilobum* | *V. trilobum* | Nursery | Unknown |  | Redwing |
| CV003 | *V. trilobum* | *V. trilobum* | Nursery | Unknown |  | Bailey Compact |
| CV004 | *V. trilobum* | *V. trilobum* | Nursery | Unknown |  | Spring Red Compact |
| CV005 | *V. trilobum* | *V. trilobum* | Nursery | Unknown |  | Spring Red Compact |
| CV006 | *V. trilobum* | *V. trilobum* | Nursery | Unknown |  | Bailey Compact |
| CV007 | *V. trilobum* | *V. opulus* | Nursery | Unknown |  | Jewell Box |
| CV008 | *V. trilobum* | *V. opulus* | Nursery | Unknown |  | Jewell Box |
| CV010 | *V. trilobum* | *V. trilobum* | Nursery | Unknown |  | Spring Green Compact |
| CV011 | *V. trilobum* | *V. opulus* | Nursery | Unknown |  | Wentworth |
| CV012 | *V. trilobum* | *V. opulus* | Nursery | Unknown |  | Wentworth |
| CV013 | *V. trilobum* | *V. trilobum* | Nursery | Unknown |  | Phillips |
| CV014 | *V. trilobum* | *V. opulus* | Nursery | Unknown |  |  |
| CV015 | *V. trilobum* | *V. trilobum* | Nursery | Unknown |  | Alfredo |
| CV016 | *V. trilobum* | *V. opulus* | Nursery | Unknown |  | Andrews |
| CV017 | *V. sargentii* | Vo × Vs | Nursery | Unknown |  |  |
| CV018 | *V. sargentii* | Vo × Vs | Nursery | Unknown |  |  |
| CV019 | *V. sargentii* | Vo × Vs | Nursery | Unknown |  |  |
| CV020 | *V. sargentii* | Vo × Vs | Nursery | Unknown |  |  |
| CV021 | *V. sargentii* | *V. opulus* | Nursery | Unknown |  | Chiquita |
| CV022 | *V. sargentii* | Vo × Vs | Nursery | Unknown |  | Flavum |
| CV023 | *V. sargentii* | Vo × Vs | Nursery | Unknown |  | Onondaga |
| CV024 | *V. sargentii* | Vo × Vs | Nursery | Unknown |  | Susquehanna |
| CV025 | *V. sargentii* | Vo × Vs | Nursery | Unknown |  | var. calvescens |
| CV026 | *V. opulus* | *V. opulus* | Nursery | Unknown |  | Aureum |
| CV027 | *V. opulus* | Vo × Vs | Nursery | Unknown |  | Losely's Compact |
| CV028 | *V. opulus* | *V. opulus* | Nursery | Unknown |  | Losely's Compact |
| CV029 | *V. opulus* | *V. opulus* | Nursery | Unknown |  | Notcutt's Variety |
| CV030 | *V. opulus* | *V. opulus* | Nursery | Unknown |  | Roseum |
| CV031 | *V. opulus* | *V. opulus* | Nursery | Unknown |  | Xanthocarpum |
| CV009 | *V. trilobum* | NA | Nursery | Unknown |  | Spring Green Compact |
| RO001 | Unknown | *V. opulus* | Arb./Bot. Gar. | Unknown | CFC: RO001 |  |
| RO002 | Unknown | *V. trilobum* | Wild | Known |  |  |
| RO003 | Unknown | *V. trilobum* | Wild | Known |  |  |
| RO004 | Unknown | *V. trilobum* | Wild | Known |  |  |
| RO005 | Unknown | *V. trilobum* | Wild | Known |  |  |
| KB001 | *V. trilobum* | *V. trilobum* | Arb./Bot. Gar. | Unknown | CMBG: 2010-0098 |  |
| KB002 | *V. trilobum* | *V. opulus* | Wild | Known |  |  |
| KB003 | *V. trilobum* | *V. opulus* | Wild | Known |  |  |
| KB004 | *V. trilobum* | *V. opulus* | Wild | Known |  |  |
| KB005 | *V. trilobum* | *V. opulus* | Wild | Known |  |  |
| TP005 | *V. opulus* | *V. trilobum* | Wild | Known |  |  |
| TP001 | *V. opulus* | *V. trilobum* | Wild | Known |  |  |
| TP002 | *V. opulus* | *V. trilobum* | Wild | Known |  |  |
| TP003 | Unknown | *V. trilobum* | Wild | Known |  |  |
| TP004 | *V. opulus* | *V. trilobum* | Wild | Known |  |  |
| PP005 | *V. trilobum* | *V. opulus* | Wild | Known |  |  |
| PP001 | *V. trilobum* | Vt × Vo | Wild | Known |  |  |
| PP002 | Unknown | Vt × Vo | Wild | Known |  |  |
| PP003 | Unknown | Vt × Vo | Wild | Known |  |  |
| PP004 | *V. trilobum* | Vt × Vo | Wild | Known |  |  |
| PP006 | Unknown | *V. opulus* | Wild | Known |  |  |
| NM001 | *V. opulus* | *V. opulus* | Arb./Bot. Gar. | Unknown | GEO: 1000220 | Xanthocarpum |
| NM002 | *V. opulus* | *V. opulus* | Arb./Bot. Gar. | Unknown | GEO: 1000250 | Xanthocarpum |
| NM003 | *V. opulus* | *V. opulus* | Arb./Bot. Gar. | Unknown | GEO: 1000336 | Xanthocarpum |
| NM004 | *V. opulus* | *V. opulus* | Arb./Bot. Gar. | Unknown | GEO: 10001606 | Fructuluteo |
| NM005 | *V. trilobum* | *V. trilobum* | Arb./Bot. Gar. | Unknown | GEO: 201617 |  |
| KK001 | *V. edule* | *V. edule* | Arb./Bot. Gar. | Known | GOTH: 2014-0739/1 |  |
| KK002 | *V. sargentii* | Vo × Vs | Arb./Bot. Gar. | Unknown | GOTH: 1921-0147/1 | var. calvescens |
| KK003 | *V. trilobum* | *V. trilobum* | Arb./Bot. Gar. | Unknown | GOTH: 2017-0587/2 |  |
| SG014 | *V. trilobum* | *V. trilobum* | Wild | Known |  |  |
| SG001 | *V. opulus* | *V. opulus* | Wild | Known |  |  |
| SG002 | *V. opulus* | *V. opulus* | Wild | Known |  |  |
| SG003 | *V. opulus* | *V. opulus* | Wild | Known |  |  |
| SG005 | *V. opulus* | *V. opulus* | Wild | Known |  |  |
| SG006 | *V. opulus* | *V. opulus* | Wild | Known |  |  |
| SG007 | *V. opulus* | *V. opulus* | Wild | Known |  |  |
| SG008 | *V. opulus* | *V. opulus* | Wild | Known |  |  |
| SG009 | *V. trilobum* | *V. trilobum* | Wild | Known |  |  |
| SG010 | *V. opulus* | *V. opulus* | Wild | Known |  |  |
| SG011 | *V. opulus* | *V. opulus* | Wild | Known |  |  |
| SG012 | *V. opulus* | *V. opulus* | Wild | Known |  |  |
| SG013 | *V. opulus* | Vt × Vo | Wild | Known |  |  |
| SG015 | *V. opulus* | *V. opulus* | Wild | Known |  |  |
| SG016 | *V. opulus* | *V. opulus* | Wild | Known |  |  |
| SG016_1 | *V. opulus* | *V. opulus* | Wild | Known |  |  |
| SG017 | *V. opulus* | *V. opulus* | Wild | Known |  |  |
| SG018 | *V. trilobum* | NA | Wild | Known |  |  |
| SG004 | *V. opulus* | NA | Wild | Known |  |  |
| MIN005 | *V. opulus* | *V. opulus* | Herbarium | Known | MIN: 943905 |  |
| MIN005_1 | *V. opulus* | *V. opulus* | Herbarium | Known | MIN: 943905 |  |
| MIN006 | *V. trilobum* | *V. trilobum* | Herbarium | Known | MIN: 291305 |  |
| MIN008 | *V. trilobum* | *V. trilobum* | Herbarium | Known | MIN: 575490 |  |
| MIN009 | *V. opulus* | *V. opulus* | Herbarium | Unknown | MIN: 949946 |  |
| MIN010 | *V. opulus* | *V. opulus* | Herbarium | Known | MIN: 949354 |  |
| MIN012 | *V. opulus* | *V. opulus* | Herbarium | Known | MIN: 620890 |  |
| MIN013 | *V. opulus* | *V. opulus* | Herbarium | Known | MIN: 640665 |  |
| MIN014 | *V. opulus* | *V. opulus* | Herbarium | Known | MIN: 920365 |  |
| MIN015 | *V. trilobum* | *V. trilobum* | Herbarium | Known | MIN: 28946 |  |
| MIN016 | *V. trilobum* | *V. trilobum* | Herbarium | Known | MIN: 739317 |  |
| MIN017 | *V. trilobum* | *V. trilobum* | Herbarium | Known | MIN: 520232 |  |
| MIN018 | *V. trilobum* | *V. trilobum* | Herbarium | Known | MIN: 921057 |  |
| MIN019 | *V. trilobum* | *V. trilobum* | Herbarium | Known | MIN: 812726 |  |
| MIN021 | *V. trilobum* | *V. trilobum* | Herbarium | Known | MIN: 773842 |  |
| MIN022 | *V. trilobum* | *V. trilobum* | Herbarium | Known | MIN: 459169 |  |
| MIN023 | *V. trilobum* | *V. trilobum* | Herbarium | Known | MIN: 774237 |  |
| MIN024 | *V. trilobum* | *V. trilobum* | Herbarium | Known | MIN: 686924 |  |
| MIN025 | *V. opulus* | *V. opulus* | Herbarium | Known | MIN: 493778 |  |
| MIN026 | *V. opulus* | *V. opulus* | Herbarium | Known | MIN: 919311 |  |
| MIN027 | *V. opulus* | *V. opulus* | Herbarium | Known | MIN: 905211 |  |
| MIN028 | *V. opulus* | *V. opulus* | Herbarium | Known | MIN: 904516 |  |
| MIN029 | *V. opulus* | *V. opulus* | Herbarium | Known | MIN: 948408 |  |
| MIN030 | *V. opulus* | *V. opulus* | Herbarium | Known | MIN: 950611 |  |
| MIN031 | *V. opulus* | *V. opulus* | Herbarium | Known | MIN: 479195 |  |
| MIN032 | *V. opulus* | *V. opulus* | Herbarium | Known | MIN: 744935 |  |
| MIN033 | *V. opulus* | *V. opulus* | Herbarium | Known | MIN: 428911 |  |
| MIN001 | *V. trilobum* | NA | Herbarium | Known | MIN: 451780 |  |
| MIN002 | *V. trilobum* | NA | Herbarium | Known | MIN: 546831 |  |
| MIN003 | Unknown | NA | Herbarium | Known | MIN: 351936 |  |
| MIN004 | *V. trilobum* | NA | Herbarium | Known | MIN: 295329 |  |
| MIN007 | *V. trilobum* | NA | Herbarium | Known | MIN: 530821 |  |
| MIN011 | *V. opulus* | NA | Herbarium | Known | MIN: 714528 |  |
| MOR001 | *V. opulus* | *V. opulus* | Herbarium | Known | MOR: 172850 |  |
| MOR002 | *V. opulus* | *V. opulus* | Herbarium | Known | MOR: 183336 |  |
| MOR003 | *V. opulus* | *V. opulus* | Herbarium | Unknown | MOR: 120568 |  |
| MOR004 | *V. opulus* | *V. opulus* | Herbarium | Known | MOR: 97815 |  |
| MOR005 | *V. opulus* | *V. opulus* | Herbarium | Unknown | MOR: 137375 |  |
| MOR006 | *V. opulus* | *V. opulus* | Herbarium | Known | MOR: 97814 |  |
| MOR007 | *V. opulus* | *V. opulus* | Herbarium | Unknown | MOR: 120570 |  |
| MOR008 | *V. opulus* | *V. opulus* | Herbarium | Known | MOR: 159715 |  |
| MOR009 | *V. opulus* | *V. opulus* | Herbarium | Known | MOR: 176853 |  |
| MOR010 | *V. opulus* | *V. opulus* | Herbarium | Known | MOR: 176854 |  |
| MOR011 | *V. trilobum* | *V. trilobum* | Herbarium | Unknown | MOR: 98114 |  |
| MOR012 | *V. trilobum* | *V. trilobum* | Herbarium | Known | MOR: 132050 |  |
| MOR013 | *V. trilobum* | *V. trilobum* | Herbarium | Known | MOR: 183324 |  |
| MOR014 | *V. opulus* | *V. opulus* | Herbarium | Known | MOR: 162992 |  |
| MOR015 | *V. opulus* | *V. opulus* | Herbarium | Known | MOR: 171777 |  |
| MOR017 | *V. trilobum* | *V. sargentii* | Herbarium | Unknown | MOR: 91193 |  |
| MOR018 | *V. trilobum* | *V. trilobum* | Herbarium | Unknown | MOR: 151811 |  |
| MOR019 | *V. opulus* | *V. trilobum* | Herbarium | Known | MOR: 146441 |  |
| MOR021 | *V. opulus* | *V. opulus* | Herbarium | Known | MOR: 89395 |  |
| MOR022 | *V. opulus* | *V. opulus* | Herbarium | Known | MOR: 137044 |  |
| MOR023 | *V. opulus* | *V. opulus* | Herbarium | Known | MOR: 89725 |  |
| MOR025 | *V. opulus* | *V. opulus* | Herbarium | Known | MOR: 148548 |  |
| MOR026 | *V. opulus* | *V. opulus* | Herbarium | Known | MOR: 47749 |  |
| MOR027 | *V. opulus* | *V. opulus* | Herbarium | Known | MOR: 3295 |  |
| MOR028 | *V. opulus* | *V. opulus* | Herbarium | Known | MOR: 23612 |  |
| MOR029 | *V. opulus* | *V. opulus* | Herbarium | Known | MOR: 104046 |  |
| MOR030 | *V. opulus* | *V. opulus* | Herbarium | Known | MOR: 3294 |  |
| MOR032 | *V. opulus* | *V. opulus* | Herbarium | Known | MOR: 137044 |  |
| MOR033 | *V. opulus* | *V. opulus* | Herbarium | Known | MOR: 123821 |  |
| MOR034 | *V. opulus* | *V. opulus* | Herbarium | Known | MOR: 112297 |  |
| MOR035 | *V. opulus* | *V. opulus* | Herbarium | Known | MOR: 57839 |  |
| MOR036 | *V. opulus* | *V. opulus* | Herbarium | Known | MOR: 26855 |  |
| MOR037 | *V. opulus* | NA | Herbarium | Known | MOR: 62490 |  |
| MOR038 | *V. opulus* | *V. opulus* | Herbarium | Known | MOR: 52911 |  |
| MOR039 | *V. opulus* | *V. opulus* | Herbarium | Known | MOR: 162533 |  |
| MOR040 | *V. opulus* | *V. opulus* | Herbarium | Known | MOR: 3296 |  |
| MOR041 | *V. opulus* | *V. opulus* | Herbarium | Known | MOR: 57461 |  |
| MOR046 | *V. trilobum* | *V. trilobum* | Herbarium | Known | MOR: 172967 |  |
| MOR047 | *V. trilobum* | *V. trilobum* | Herbarium | Known | MOR: 93978 |  |
| MOR048 | *V. trilobum* | *V. trilobum* | Herbarium | Known | MOR: 168315 |  |
| MOR049 | *V. trilobum* | *V. trilobum* | Herbarium | Known | MOR: 67297 |  |
| MOR016 | *V. trilobum* | NA | Herbarium | Unknown | MOR: 54247 | var. compactum |
| MOR020 | *V. opulus* | NA | Herbarium | Known | MOR: 83598 |  |
| MOR024 | *V. opulus* | NA | Herbarium | Known | MOR: 161704 |  |
| MOR031 | *V. opulus* | NA | Herbarium | Known | MOR: 03307 |  |
| MOR042 | *V. opulus* | NA | Herbarium | Known | MOR: 55318 |  |
| MOR043 | *V. opulus* | NA | Herbarium | Known | MOR: 179789 |  |
| MOR044 | *V. trilobum* | NA | Herbarium | Known | MOR: 103719 |  |
| MOR045 | *V. trilobum* | NA | Herbarium | Known | MOR: 61133 |  |
| KBG001 | *V. opulus* | *V. opulus* | Herbarium | Known | K: K001309408 |  |
| KBG002 | *V. opulus* | *V. opulus* | Herbarium | Known | K: K001309208 |  |
| KBG003 | *V. opulus* | *V. opulus* | Herbarium | Known | K: K001309209 |  |
| KBG004 | *V. opulus* | *V. opulus* | Herbarium | Known | K: K001309215 |  |
| KBG005 | *V. opulus* | *V. opulus* | Herbarium | Known | K: K001309214 |  |
| RJ004 | *V. opulus* | *V. opulus* | Arb./Bot. Gar. | Known | HOW: AK 7 |  |
| RJ009 | *V. opulus* | *V. opulus* | Arb./Bot. Gar. | Known | HOW: CJLU 189 |  |
| RJ003 | *V. opulus* | *V. opulus* | Arb./Bot. Gar. | Known | HOW: ABG 93/ 14 |  |
| RJ014 | *V. opulus* | *V. opulus* | Arb./Bot. Gar. | Known | HOW: UTBG 00/ 65 |  |
| RJ002 | *V. edule* | *V. edule* | Arb./Bot. Gar. | Known | HOW: EHBC 159 |  |
| RJ005 | *V. opulus* | *V. opulus* | Arb./Bot. Gar. | Known | HOW: BBG 92/ 160 |  |
| RJ006 | *V. opulus* | *V. opulus* | Arb./Bot. Gar. | Known | HOW: BBG 93/ 186 |  |
| RJ007 | *V. opulus* | *V. opulus* | Arb./Bot. Gar. | Known | HOW: BBG 95/ 223 |  |
| RJ008 | *V. opulus* | *V. opulus* | Arb./Bot. Gar. | Known | HOW: BBG 97/ 311 |  |
| RJ010 | *V. opulus* | *V. opulus* | Arb./Bot. Gar. | Known | HOW: D&V 99/ 13 |  |
| RJ011 | *V. opulus* | *V. opulus* | Arb./Bot. Gar. | Known | HOW: EHS 89 |  |
| RJ012 | *V. opulus* | NA | Arb./Bot. Gar. | Known | HOW: ENET 65 |  |
| RJ013 | *V. opulus* | *V. sargentii* | Arb./Bot. Gar. | Known | HOW: SICH 1856 |  |
| RJ015 | *V. trilobum* | *V. trilobum* | Arb./Bot. Gar. | Known | HOW: NEUSA 101 |  |
| RJ016 | *V. sargentii* | *V. sargentii* | Arb./Bot. Gar. | Known | HOW: SICH 2038 |  |
| RJ017 | *V. sargentii* | NA | Arb./Bot. Gar. | Known | HOW: QPH 96/ 222 |  |
| RJ001 | *V. edule* | NA | Arb./Bot. Gar. | Known | HOW: EHBC 5 |  |
| BP001 | *V. trilobum* | *V. trilobum* | Wild | Known |  |  |
| MY001 | *V. trilobum* | *V. trilobum* | Nursery | Unknown |  | Redwing |
| MY002 | *V. opulus* | *V. opulus* | Wild | Known |  |  |
| HV004 | *V. trilobum* | *V. trilobum* | Arb./Bot. Gar. | Known | KAIS: 1994-0379 |  |
| HV005 | *V. opulus* | *V. opulus* | Arb./Bot. Gar. | Unknown | KAIS: ZZ-0544 |  |
| HV007 | *V. opulus* | Vt × Vo | Arb./Bot. Gar. | Unknown | KAIS: ZZ-0550 |  |
| HV008 | *V. opulus* | *V. edule* | Arb./Bot. Gar. | Known | KUMP: 1994-0870 |  |
| HV002 | *V. sargentii* | *V. sargentii* | Arb./Bot. Gar. | Known | KUMP: 2008-0119 |  |
| HV001 | *V. trilobum* | *V. trilobum* | Arb./Bot. Gar. | Known | KUMP: 1984-0098 |  |
| HV003 | *V. sargentii* | Vt × Vo | Arb./Bot. Gar. | Known | KUMP: 1995-0589 |  |
| HV006 | *V. sargentii* | *V. trilobum* | Arb./Bot. Gar. | Known | KUMP: 1995-0589 |  |
| HV009 | *V. sargentii* | *V. sargentii* | Arb./Bot. Gar. | Known | KUMP: 1994-0930 |  |
| JCz001 | *V. trilobum* | *V. trilobum* | Arb./Bot. Gar. | Unknown | WIS: 02077 | Redwing |
| JCz002 | *V. opulus* | *V. opulus* | Arb./Bot. Gar. | Unknown | WIS: 2005-085*A | Park Harvest |
| JCz003 | *V. sargentii* | *V. sargentii* | Arb./Bot. Gar. | Unknown | WIS: 1980-214*A | Susquehanna |
| JCz004 | *V. trilobum* | *V. trilobum* | Arb./Bot. Gar. | Unknown | WIS: 90201 | Andrews |
| JCz005 | *V. sargentii* | *V. sargentii* | Arb./Bot. Gar. | Unknown | WIS: JCz005 | Flavum |
| JCz006 | *V. sargentii* | *V. sargentii* | Arb./Bot. Gar. | Unknown | WIS: 1995-171 | f. puberulum |
| JCz007 | *V. sargentii* | Vo × Vs | Arb./Bot. Gar. | Unknown | WIS: 2003-043 | Onondaga |
| DS001 | *V. trilobum* | *V. opulus* | Arb./Bot. Gar. | Unknown | WIS: 87226 | Wentworth |
| DS002 | *V. opulus* | *V. opulus* | Arb./Bot. Gar. | Unknown | WIS: DS002 | Xanthocarpum |
| KBa001 | *V. opulus* | Vo × Vs | Arb./Bot. Gar. | Unknown | MEISE: 19535330 |  |
| KBa002 | *V. opulus* | *V. opulus* | Arb./Bot. Gar. | Unknown | MEISE: 19812112 |  |
| KBa003 | *V. trilobum* | *V. opulus* | Arb./Bot. Gar. | Unknown | MEISE: 19562627 |  |
| KBa004 | *V. trilobum* | *V. opulus* | Arb./Bot. Gar. | Known | MEISE: 20111085-75 |  |
| KBa005 | *V. sargentii* | Vo × Vs | Arb./Bot. Gar. | Unknown | MEISE: 19554860 |  |
| MC001 | *V. opulus* | *V. opulus* | Wild | Known |  |  |
| SH001 | Unknown | *V. opulus* | Landscape | Unknown |  |  |
| SH002 | Unknown | *V. opulus* | Landscape | Unknown |  |  |
| SH003 | Unknown | *V. opulus* | Landscape | Unknown |  |  |
| SH004 | Unknown | *V. opulus* | Landscape | Unknown |  |  |
| SH005 | Unknown | *V. opulus* | Landscape | Unknown |  |  |
| SH006 | Unknown | *V. opulus* | Landscape | Unknown |  |  |
| SH007 | Unknown | *V. opulus* | Landscape | Unknown |  |  |
| SH008 | Unknown | *V. opulus* | Landscape | Unknown |  |  |
| SH009 | Unknown | *V. opulus* | Landscape | Unknown |  |  |
| DT001 | *V. trilobum* | *V. opulus* | Arb./Bot. Gar. | Unknown | MNLA: 19850121 | Wentworth |
| DT002 | *V. trilobum* | *V. trilobum* | Arb./Bot. Gar. | Unknown | MNLA: 20030313 | Hahs |
| GT001 | *V. trilobum* | Vo × Vs | Arb./Bot. Gar. | Unknown | MBG: 2000-2263-5 |  |
| GT002 | *V. trilobum* | Vo × Vs | Arb./Bot. Gar. | Unknown | MBG: 2000-2263-7 |  |
| GT003 | *V. trilobum* | *V. opulus* | Arb./Bot. Gar. | Known | MBG: 2014-1233-5 |  |
| GT004 | *V. trilobum* | *V. opulus* | Arb./Bot. Gar. | Known | MBG: 2014-1233-4 |  |
| GT005 | *V. opulus* | *V. opulus* | Arb./Bot. Gar. | Unknown | MBG: 1977-2419-2 |  |
| GT006 | *V. opulus* | *V. opulus* | Arb./Bot. Gar. | Unknown | MBG: 1987-2550-1 |  |
| GT007 | *V. trilobum* | Vo × Vs | Arb./Bot. Gar. | Unknown | MBG: 2009-0492-1 |  |
| GT008 | *V. trilobum* | Vo × Vs | Arb./Bot. Gar. | Unknown | MBG: 2010-1301-1 |  |
| GT009 | *V. trilobum* | Vo × Vs | Arb./Bot. Gar. | Unknown | MBG: 2010-1297-1 |  |
| GT010 | *V. opulus* | *V. opulus* | Arb./Bot. Gar. | Unknown | MBG: 2003-1400-1 |  |
| JZ015 | *V. trilobum* | *V. trilobum* | Wild | Known |  |  |
| JZ036 | *V. trilobum* | *V. trilobum* | Wild | Known |  |  |
| JZ033 | *V. trilobum* | *V. trilobum* | Wild | Known |  |  |
| JZ001 | *V. trilobum* | *V. trilobum* | Wild | Known |  |  |
| JZ002 | *V. trilobum* | *V. trilobum* | Wild | Known |  |  |
| JZ003 | *V. trilobum* | *V. trilobum* | Wild | Known |  |  |
| JZ004 | *V. trilobum* | *V. trilobum* | Wild | Known |  |  |
| JZ005 | *V. trilobum* | *V. trilobum* | Wild | Known |  |  |
| JZ006 | *V. trilobum* | *V. trilobum* | Wild | Known |  |  |
| JZ007 | *V. trilobum* | *V. trilobum* | Wild | Known |  |  |
| JZ008 | *V. trilobum* | *V. trilobum* | Wild | Known |  |  |
| JZ009 | *V. trilobum* | *V. trilobum* | Wild | Known |  |  |
| JZ010 | *V. trilobum* | *V. trilobum* | Wild | Known |  |  |
| JZ011 | *V. trilobum* | *V. trilobum* | Wild | Known |  |  |
| JZ012 | *V. trilobum* | *V. trilobum* | Wild | Known |  |  |
| JZ013 | *V. trilobum* | *V. trilobum* | Wild | Known |  |  |
| JZ014 | *V. trilobum* | *V. trilobum* | Wild | Known |  |  |
| JZ016 | *V. trilobum* | *V. trilobum* | Wild | Known |  |  |
| JZ017 | *V. trilobum* | *V. trilobum* | Wild | Known |  |  |
| JZ018 | *V. trilobum* | *V. trilobum* | Wild | Known |  |  |
| JZ019 | *V. trilobum* | *V. trilobum* | Wild | Known |  |  |
| JZ020 | *V. trilobum* | *V. trilobum* | Wild | Known |  |  |
| JZ021 | *V. trilobum* | *V. trilobum* | Wild | Known |  |  |
| JZ022 | *V. trilobum* | *V. trilobum* | Wild | Known |  |  |
| JZ023 | *V. trilobum* | *V. trilobum* | Wild | Known |  |  |
| JZ024 | *V. trilobum* | *V. trilobum* | Wild | Known |  |  |
| JZ025 | *V. trilobum* | *V. trilobum* | Wild | Known |  |  |
| JZ026 | *V. trilobum* | *V. trilobum* | Wild | Known |  |  |
| JZ027 | *V. trilobum* | *V. trilobum* | Wild | Known |  |  |
| JZ028 | *V. trilobum* | *V. trilobum* | Wild | Known |  |  |
| JZ029 | *V. trilobum* | *V. trilobum* | Wild | Known |  |  |
| JZ030 | *V. trilobum* | *V. trilobum* | Wild | Known |  |  |
| JZ031 | *V. trilobum* | *V. trilobum* | Wild | Known |  |  |
| JZ031_1 | *V. trilobum* | *V. trilobum* | Wild | Known |  |  |
| JZ032 | *V. trilobum* | *V. trilobum* | Wild | Known |  |  |
| JZ034 | *V. trilobum* | *V. trilobum* | Wild | Known |  |  |
| JZ035 | *V. trilobum* | *V. trilobum* | Wild | Known |  |  |
| JZ037 | *V. trilobum* | *V. trilobum* | Wild | Known |  |  |
| JZ038 | *V. trilobum* | *V. trilobum* | Wild | Known |  |  |
| JZ039 | *V. trilobum* | *V. trilobum* | Wild | Known |  |  |
| JZ040 | *V. trilobum* | *V. trilobum* | Wild | Known |  |  |
| JZ041 | *V. trilobum* | *V. trilobum* | Wild | Known |  |  |
| JZ042 | *V. trilobum* | *V. trilobum* | Wild | Known |  |  |
| DT090 | *V. opulus* | Vo × Vs | Wild | Known |  |  |
| DT078 | *V. trilobum* | *V. trilobum* | Nursery | Unknown |  |  |
| DT079 | *V. trilobum* | *V. trilobum* | Nursery | Unknown |  |  |
| DT080 | *V. trilobum* | *V. trilobum* | Nursery | Unknown |  |  |
| DT081 | *V. trilobum* | *V. trilobum* | Nursery | Unknown |  |  |
| DT082 | *V. trilobum* | *V. trilobum* | Nursery | Unknown |  |  |
| DT083 | *V. trilobum* | *V. trilobum* | Nursery | Unknown |  |  |
| DT084 | *V. trilobum* | *V. trilobum* | Nursery | Unknown |  |  |
| DT085 | *V. trilobum* | Vo × Vs | Nursery | Unknown |  |  |
| DT086 | *V. opulus* | Vo × Vs | Wild | Known |  |  |
| DT088 | Unknown | Vo × Vs | Wild | Known |  |  |
| DT089 | Unknown | *V. opulus* | Wild | Known |  |  |
| DT077 | *V. trilobum* | NA | Nursery | Unknown |  |  |
| DT087 | Unknown | NA | Nursery | Known |  |  |
| AR001 | *V. opulus* | *V. opulus* | Arb./Bot. Gar. | Unknown | PAS: AR001 |  |
| AR002 | *V. opulus* | *V. opulus* | Arb./Bot. Gar. | Unknown | PAS: AR002 |  |
| AR003 | *V. trilobum* | *V. opulus* | Arb./Bot. Gar. | Unknown | PAS: AR003 |  |
| AR004 | *V. sargentii* | Vo × Vs | Arb./Bot. Gar. | Unknown | PAS: AR004 |  |
| PPN006 | Unknown | Vt × Vs | Nursery | Unknown |  |  |
| PPN001 | Unknown | *V. opulus* | Wild | Known |  |  |
| PPN002 | Unknown | *V. opulus* | Wild | Known |  |  |
| PPN003 | Unknown | *V. opulus* | Nursery | Unknown |  |  |
| PPN004 | Unknown | *V. opulus* | Nursery | Unknown |  |  |
| PPN005 | Unknown | *V. opulus* | Nursery | Unknown |  |  |
| RK001 | Unknown | *V. trilobum* | Nursery | Unknown |  |  |
| RK002 | Unknown | *V. trilobum* | Nursery | Unknown |  |  |
| RK003 | Unknown | *V. trilobum* | Nursery | Unknown |  |  |
| RK006 | Unknown | *V. trilobum* | Nursery | Unknown |  |  |
| RK007 | Unknown | *V. trilobum* | Nursery | Unknown |  |  |
| RK004 | Unknown | NA | Nursery | Unknown |  |  |
| RK005 | Unknown | NA | Nursery | Unknown |  |  |
| RK008 | Unknown | NA | Nursery | Unknown |  |  |
| RS001 | *V. trilobum* | *V. trilobum* | Wild | Known |  |  |
| RS002 | *V. trilobum* | *V. trilobum* | Wild | Known |  |  |
| RS003 | *V. trilobum* | *V. opulus* | Nursery | Unknown |  |  |
| RS004 | *V. trilobum* | *V. opulus* | Nursery | Unknown |  |  |
| RS005 | *V. trilobum* | *V. trilobum* | Nursery | Unknown |  |  |
| RS006 | *V. trilobum* | *V. trilobum* | Landscape | Unknown |  |  |
| DK009 | *V. opulus* | *V. opulus* | Wild | Known |  |  |
| DK007 | *V. trilobum* | *V. trilobum* | Wild | Known |  |  |
| DK002 | *V. trilobum* | *V. trilobum* | Wild | Known |  |  |
| DK001 | *V. trilobum* | *V. trilobum* | Wild | Known |  |  |
| DK003 | *V. trilobum* | *V. trilobum* | Wild | Known |  |  |
| DK004 | *V. trilobum* | *V. trilobum* | Wild | Known |  |  |
| DK005 | *V. trilobum* | *V. trilobum* | Wild | Known |  |  |
| DK006 | *V. trilobum* | *V. trilobum* | Landscape | Unknown |  |  |
| DK008 | *V. opulus* | *V. opulus* | Wild | Known |  |  |
| DK010 | *V. opulus* | *V. opulus* | Wild | Known |  |  |
| PB039 | *V. opulus* | *V. opulus* | Arb./Bot. Gar. | Known | RBGE: 20081669*B |  |
| PB013 | *V. opulus* | *V. opulus* | Arb./Bot. Gar. | Known | RBGE: 19832636*C |  |
| PB003 | *V. opulus* | *V. opulus* | Arb./Bot. Gar. | Known | RBGE: 19081036*C |  |
| PB035 | *V. opulus* | *V. sargentii* | Arb./Bot. Gar. | Known | RBGE: 20052099*Y |  |
| PB041 | *V. trilobum* | *V. trilobum* | Arb./Bot. Gar. | Known | RBGE: 20081958*A |  |
| PB045 | *V. sargentii* | *V. sargentii* | Arb./Bot. Gar. | Known | RBGE: 20151211*D |  |
| PB015 | *V. sargentii* | *V. sargentii* | Arb./Bot. Gar. | Known | RBGE: 20031031*A |  |
| PB025 | *V. sargentii* | *V. sargentii* | Arb./Bot. Gar. | Known | RBGE: 20051944*L |  |
| PB017 | *V. sargentii* | NA | Arb./Bot. Gar. | Known | RBGE: 20031032*B |  |
| PB065 | *V. edule* | *V. edule* | Arb./Bot. Gar. | Known | RBGE: 20160637*C |  |
| PB066 | *V. edule* | NA | Arb./Bot. Gar. | Known | RBGE: 20160637*D |  |
| PB067 | *V. edule* | *V. edule* | Arb./Bot. Gar. | Known | RBGE: 20160637*E |  |
| PB054 | *V. edule* | *V. edule* | Arb./Bot. Gar. | Known | RBGE: 20160638*A |  |
| PB055 | *V. edule* | *V. edule* | Arb./Bot. Gar. | Known | RBGE: 20160638*B |  |
| PB056 | *V. edule* | *V. edule* | Arb./Bot. Gar. | Known | RBGE: 20160638*C |  |
| PB057 | *V. edule* | *V. edule* | Arb./Bot. Gar. | Known | RBGE: 20160638*H |  |
| PB058 | *V. edule* | *V. edule* | Arb./Bot. Gar. | Known | RBGE: 20160638*K |  |
| PB059 | *V. edule* | *V. edule* | Arb./Bot. Gar. | Known | RBGE: 20160636*D |  |
| PB060 | *V. edule* | *V. edule* | Arb./Bot. Gar. | Known | RBGE: 20160636*G |  |
| PB061 | *V. edule* | *V. edule* | Arb./Bot. Gar. | Known | RBGE: 20160636*I |  |
| PB062 | *V. edule* | *V. edule* | Arb./Bot. Gar. | Known | RBGE: 20160636*J |  |
| PB063 | *V. edule* | *V. edule* | Arb./Bot. Gar. | Known | RBGE: 20160636*K |  |
| PB064 | *V. edule* | *V. edule* | Arb./Bot. Gar. | Known | RBGE: 20160636*L |  |
| PB001 | *V. opulus* | *V. opulus* | Arb./Bot. Gar. | Known | RBGE: 19081036*A |  |
| PB002 | *V. opulus* | *V. opulus* | Arb./Bot. Gar. | Known | RBGE: 19081036*B |  |
| PB004 | *V. opulus* | *V. opulus* | Arb./Bot. Gar. | Known | RBGE: 19081036*D |  |
| PB005 | *V. sargentii* | *V. sargentii* | Arb./Bot. Gar. | Unknown | RBGE: 19608006*A |  |
| PB006 | *V. sargentii* | *V. sargentii* | Arb./Bot. Gar. | Unknown | RBGE: 19608006*B |  |
| PB007 | *V. opulus* | *V. opulus* | Arb./Bot. Gar. | Unknown | RBGE: 19610948*A | Notcutt's Variety |
| PB008 | *V. opulus* | *V. opulus* | Arb./Bot. Gar. | Unknown | RBGE: 19699401*A |  |
| PB009 | *V. opulus* | NA | Arb./Bot. Gar. | Known | RBGE: 19800051*A |  |
| PB010 | *V. opulus* | *V. opulus* | Arb./Bot. Gar. | Known | RBGE: 19800701*B |  |
| PB011 | *V. opulus* | *V. opulus* | Arb./Bot. Gar. | Known | RBGE: 19800701*C |  |
| PB012 | *V. opulus* | *V. opulus* | Arb./Bot. Gar. | Known | RBGE: 19832636*B |  |
| PB014 | *V. sargentii* | *V. sargentii* | Arb./Bot. Gar. | Known | RBGE: 20031031*A |  |
| PB016 | *V. sargentii* | *V. sargentii* | Arb./Bot. Gar. | Known | RBGE: 20031031*A |  |
| PB018 | *V. trilobum* | *V. sargentii* | Arb./Bot. Gar. | Unknown | RBGE: 20031296*B |  |
| PB019 | *V. sargentii* | *V. sargentii* | Arb./Bot. Gar. | Known | RBGE: 20051944*A |  |
| PB020 | *V. sargentii* | *V. sargentii* | Arb./Bot. Gar. | Known | RBGE: 20051944*B |  |
| PB021 | *V. sargentii* | *V. sargentii* | Arb./Bot. Gar. | Known | RBGE: 20051944*C |  |
| PB022 | *V. sargentii* | *V. sargentii* | Arb./Bot. Gar. | Known | RBGE: 20051944*D |  |
| PB023 | *V. sargentii* | *V. sargentii* | Arb./Bot. Gar. | Known | RBGE: 20051944*E |  |
| PB024 | *V. sargentii* | *V. sargentii* | Arb./Bot. Gar. | Known | RBGE: 20051944*K |  |
| PB026 | *V. sargentii* | *V. sargentii* | Arb./Bot. Gar. | Known | RBGE: 20051944*M |  |
| PB027 | *V. opulus* | *V. sargentii* | Arb./Bot. Gar. | Known | RBGE: 20052099*A |  |
| PB028 | *V. opulus* | *V. sargentii* | Arb./Bot. Gar. | Known | RBGE: 20052099*B |  |
| PB029 | *V. opulus* | *V. sargentii* | Arb./Bot. Gar. | Known | RBGE: 20052099*L |  |
| PB030 | *V. opulus* | *V. sargentii* | Arb./Bot. Gar. | Known | RBGE: 20052099*M |  |
| PB031 | *V. opulus* | *V. sargentii* | Arb./Bot. Gar. | Known | RBGE: 20052099*P |  |
| PB032 | *V. opulus* | *V. sargentii* | Arb./Bot. Gar. | Known | RBGE: 20052099*V |  |
| PB033 | *V. opulus* | *V. sargentii* | Arb./Bot. Gar. | Known | RBGE: 20052099*W |  |
| PB034 | *V. opulus* | *V. sargentii* | Arb./Bot. Gar. | Known | RBGE: 20052099*X |  |
| PB036 | *V. opulus* | *V. sargentii* | Arb./Bot. Gar. | Known | RBGE: 20052099*Z |  |
| PB037 | *V. opulus* | *V. opulus* | Arb./Bot. Gar. | Known | RBGE: 20081669*A |  |
| PB038 | *V. opulus* | *V. opulus* | Arb./Bot. Gar. | Known | RBGE: 20081669*A |  |
| PB040 | *V. trilobum* | *V. trilobum* | Arb./Bot. Gar. | Known | RBGE: 20081958*A |  |
| PB042 | *V. trilobum* | *V. trilobum* | Arb./Bot. Gar. | Known | RBGE: 20081958*A |  |
| PB043 | *V. trilobum* | *V. trilobum* | Arb./Bot. Gar. | Known | RBGE: 20081958*A |  |
| PB044 | *V. sargentii* | *V. sargentii* | Arb./Bot. Gar. | Known | RBGE: 20151211*C |  |
| PB046 | *V. sargentii* | *V. sargentii* | Arb./Bot. Gar. | Known | RBGE: 20151211*E |  |
| PB047 | *V. opulus* | *V. opulus* | Arb./Bot. Gar. | Known | RBGE: 20181204*I |  |
| PB048 | *V. opulus* | *V. opulus* | Arb./Bot. Gar. | Known | RBGE: 20181204*M |  |
| PB049 | *V. opulus* | *V. opulus* | Arb./Bot. Gar. | Known | RBGE: 20181204*N |  |
| PB050 | *V. opulus* | *V. opulus* | Arb./Bot. Gar. | Known | RBGE: 20181204*AB |  |
| PB051 | *V. opulus* | *V. opulus* | Arb./Bot. Gar. | Known | RBGE: 20181204*AD |  |
| PB052 | *V. opulus* | *V. opulus* | Arb./Bot. Gar. | Known | RBGE: 20181204*Z |  |
| PB053 | *V. opulus* | *V. opulus* | Arb./Bot. Gar. | Unknown | RBGE: 20210154*A |  |
| ZTS001 | *V. trilobum* | *V. trilobum* | Nursery | Unknown |  |  |
| ZTS002 | *V. opulus* | *V. opulus* | Nursery | Unknown |  |  |
| ZTS003 | *V. trilobum* | *V. trilobum* | Nursery | Unknown |  |  |
| ZTS004 | *V. trilobum* | *V. trilobum* | Nursery | Unknown |  |  |
| ZTS005 | *V. trilobum* | *V. trilobum* | Nursery | Unknown |  |  |
| ZTS006 | *V. opulus* | *V. trilobum* | Nursery | Unknown |  |  |
| ZTS007 | *V. trilobum* | *V. trilobum* | Nursery | Unknown |  |  |
| ZTS008 | *V. trilobum* | *V. opulus* | Nursery | Unknown |  | Wentworth |
| ZTS009 | *V. trilobum* | *V. opulus* | Nursery | Unknown |  | Wentworth |
| MW001 | *V. sargentii* | *V. sargentii* | Arb./Bot. Gar. | Known | SBG: 2001.178A |  |
| MW002 | *V. sargentii* | *V. sargentii* | Arb./Bot. Gar. | Known | SBG: 2011.250A |  |
| AM001 | *V. opulus* | *V. opulus* | Arb./Bot. Gar. | Unknown |  |  |
| AM002 | *V. opulus* | *V. opulus* | Arb./Bot. Gar. | Unknown |  |  |
| AM002_1 | *V. opulus* | *V. opulus* | Arb./Bot. Gar. | Unknown |  |  |
| AM003 | *V. opulus* | Vo × Vs | Wild | Known |  |  |
| AM004 | *V. opulus* | *V. opulus* | Wild | Known |  |  |
| AM005 | *V. trilobum* | *V. opulus* | Arb./Bot. Gar. | Unknown |  |  |
| AM006 | Unknown | *V. opulus* | Wild | Known |  |  |
| DM001 | *V. trilobum* | *V. trilobum* | Wild | Known |  |  |
| TS001 | *V. opulus* | *V. opulus* | Arb./Bot. Gar. | Unknown | SZAR: TS001 |  |
| TS002 | *V. opulus* | *V. opulus* | Arb./Bot. Gar. | Unknown | SZAR: TS002 |  |
| TS003 | *V. opulus* | *V. opulus* | Arb./Bot. Gar. | Unknown | SZAR: TS003 |  |
| TS004 | *V. opulus* | *V. opulus* | Arb./Bot. Gar. | Unknown | SZAR: TS004 |  |
| TS005 | *V. opulus* | *V. opulus* | Arb./Bot. Gar. | Unknown | SZAR: TS005 |  |
| TS006 | *V. opulus* | *V. opulus* | Arb./Bot. Gar. | Unknown | SZAR: TS006 |  |
| TS007 | *V. opulus* | *V. opulus* | Arb./Bot. Gar. | Unknown | SZAR: TS007 |  |
| TS008 | *V. opulus* | *V. opulus* | Arb./Bot. Gar. | Unknown | SZAR: TS008 |  |
| TS009 | *V. opulus* | *V. opulus* | Arb./Bot. Gar. | Unknown | SZAR: TS009 |  |
| TS010 | *V. opulus* | *V. opulus* | Arb./Bot. Gar. | Unknown | SZAR: TS010 |  |
| DJ001 | *V. sargentii* | *V. sargentii* | Arb./Bot. Gar. | Known | AA: 287-2002*A |  |
| DJ002 | *V. sargentii* | *V. sargentii* | Arb./Bot. Gar. | Known | AA: 287-2002*B |  |
| DJ003 | *V. sargentii* | *V. sargentii* | Arb./Bot. Gar. | Known | AA: 87-2015*A |  |
| DJ004 | *V. sargentii* | *V. sargentii* | Arb./Bot. Gar. | Known | AA: 87-2015*B |  |
| DJ005 | *V. sargentii* | *V. sargentii* | Arb./Bot. Gar. | Known | AA: 87-2015*C |  |
| DJ006 | *V. sargentii* | *V. sargentii* | Arb./Bot. Gar. | Known | AA: 87-2015*D |  |
| DJ007 | *V. trilobum* | *V. trilobum* | Arb./Bot. Gar. | Known | AA: 192-2017*A |  |
| DJ008 | *V. trilobum* | *V. trilobum* | Arb./Bot. Gar. | Known | AA: 192-2017*B |  |
| DJ009 | *V. trilobum* | *V. trilobum* | Arb./Bot. Gar. | Known | AA: 192-2017*C |  |
| DJ010 | *V. opulus* | *V. opulus* | Arb./Bot. Gar. | Known | AA: 352-78*A |  |
| DJ011 | *V. opulus* | *V. opulus* | Arb./Bot. Gar. | Known | AA: 352-78*C |  |
| DJ012 | *V. sargentii* | *V. sargentii* | Arb./Bot. Gar. | Known | AA: 719-88*A |  |
| DJ013 | *V. sargentii* | *V. sargentii* | Arb./Bot. Gar. | Known | AA: 719-88*B |  |
| DJ014 | *V. sargentii* | *V. sargentii* | Arb./Bot. Gar. | Known | AA: 719-88*C |  |
| DJ015 | *V. trilobum* | *V. trilobum* | Arb./Bot. Gar. | Known | AA: 361-2006*A |  |
| DJ016 | *V. trilobum* | *V. trilobum* | Arb./Bot. Gar. | Known | AA: 361-2006*B |  |
| DJ017 | *V. trilobum* | Vo × Vs | Arb./Bot. Gar. | Unknown | AA: 1097-60*A |  |
| DJ018 | *V. opulus* | *V. opulus* | Arb./Bot. Gar. | Known | AA: 46-2006*A |  |
| DJ019 | *V. sargentii* | *V. sargentii* | Arb./Bot. Gar. | Known | AA: 1922-80*A |  |
| DJ020 | *V. sargentii* | *V. sargentii* | Arb./Bot. Gar. | Known | AA: 1922-80*C |  |
| DJ021 | *V. sargentii* | *V. sargentii* | Arb./Bot. Gar. | Known | AA: 379-97*A |  |
| DJ022 | *V. sargentii* | *V. sargentii* | Arb./Bot. Gar. | Known | AA: 379-97*B |  |
| DJ023 | *V. sargentii* | *V. sargentii* | Arb./Bot. Gar. | Known | AA: 1897-80*B |  |
| DJ024 | *V. sargentii* | *V. sargentii* | Arb./Bot. Gar. | Known | AA: 1897-80*C |  |
| DJ025 | *V. sargentii* | *V. sargentii* | Arb./Bot. Gar. | Known | AA: 79-90*A |  |
| DJ026 | *V. sargentii* | *V. sargentii* | Arb./Bot. Gar. | Known | AA: 79-90*B |  |
| DJ027 | *V. sargentii* | Vo × Vs | Arb./Bot. Gar. | Unknown | AA: 140-2013*A | Onondaga |
| DJ028 | *V. sargentii* | Vo × Vs | Arb./Bot. Gar. | Unknown | AA: 158-2004*A | Onondaga |
| DJ029 | *V. sargentii* | *V. sargentii* | Arb./Bot. Gar. | Unknown | AA: 398-68*B | f. flavum |
| BA001 | *V. trilobum* | *V. trilobum* | Arb./Bot. Gar. | Known | BREN: ND 18-180 |  |
| BA002 | *V. trilobum* | *V. trilobum* | Arb./Bot. Gar. | Known | BREN: ND 18-178 |  |
| BA003 | *V. trilobum* | *V. trilobum* | Arb./Bot. Gar. | Known | BREN: IA 20-104 |  |
| BA004 | *V. trilobum* | *V. trilobum* | Arb./Bot. Gar. | Known | BREN: ND 18-182 |  |
| BA005 | *V. trilobum* | *V. trilobum* | Arb./Bot. Gar. | Known | BREN: ND 18-177 |  |
| BA006 | *V. trilobum* | *V. trilobum* | Arb./Bot. Gar. | Known | BREN: IA 20-106 |  |
| BA007 | *V. trilobum* | *V. trilobum* | Arb./Bot. Gar. | Known | BREN: ND 18-181 |  |
| BA008 | *V. trilobum* | *V. trilobum* | Arb./Bot. Gar. | Known | BREN: ND 18-179 |  |
| BA009 | *V. trilobum* | *V. trilobum* | Arb./Bot. Gar. | Known | BREN: IA 20-105 |  |
| BA010 | *V. trilobum* | *V. trilobum* | Arb./Bot. Gar. | Known | BREN: IA 20-107 |  |
| GP020 | *V. opulus* | *V. opulus* | Arb./Bot. Gar. | Known | DAWE: D2002-0454.003 |  |
| GP001 | *V. trilobum* | *V. trilobum* | Arb./Bot. Gar. | Known | DAWE: D1988-0440.001 |  |
| GP002 | *V. opulus* | *V. opulus* | Arb./Bot. Gar. | Unknown | DAWE: D1985-0209.001 |  |
| GP003 | *V. sargentii* | *V. sargentii* | Arb./Bot. Gar. | Unknown | DAWE: D1999-1608.001 | Onondaga |
| GP004 | *V. sargentii* | *V. sargentii* | Arb./Bot. Gar. | Unknown | DAWE: D2000-0965.001 | Susquehanna |
| GP005 | *V. sargentii* | *V. sargentii* | Arb./Bot. Gar. | Unknown | DAWE: D1995-0668.002 | f. calvescens |
| GP006 | *V. sargentii* | *V. sargentii* | Arb./Bot. Gar. | Unknown | DAWE: D1995-0668.003 | f. calvescens |
| GP007 | *V. sargentii* | *V. sargentii* | Arb./Bot. Gar. | Known | DAWE: D1994-0680.003 | f. puberulum |
| GP008 | *V. trilobum* | *V. trilobum* | Arb./Bot. Gar. | Unknown | DAWE: D1992-0318.002 | Manitou |
| GP009 | *V. sargentii* | *V. sargentii* | Arb./Bot. Gar. | Known | DAWE: D1994-0680.001 | f. puberulum |
| GP010 | *V. sargentii* | *V. sargentii* | Arb./Bot. Gar. | Known | DAWE: D1994-0680.002 | f. puberulum |
| GP011 | *V. sargentii* | Vo × Vs | Arb./Bot. Gar. | Unknown | DAWE: D1995-0668.001 | f. calvescens |
| GP012 | *V. opulus* | *V. opulus* | Arb./Bot. Gar. | Unknown | DAWE: D2003-0674.001 | Losely's Compact |
| GP013 | *V. opulus* | *V. trilobum* | Arb./Bot. Gar. | Unknown | DAWE: D2003-0391.002 | Leonard's Dwarf |
| GP014 | *V. opulus* | *V. opulus* | Arb./Bot. Gar. | Unknown | DAWE: D2002-1093.001 | Bullatum |
| GP015 | *V. opulus* | *V. opulus* | Arb./Bot. Gar. | Unknown | DAWE: D1991-0262.001 |  |
| GP016 | *V. opulus* | *V. opulus* | Arb./Bot. Gar. | Unknown | DAWE: D2005-0498.001 | Park Harvest |
| GP017 | *V. sargentii* | *V. sargentii* | Arb./Bot. Gar. | Unknown | DAWE: D1988-0740.001 | f. flavum |
| GP018 | *V. opulus* | *V. opulus* | Arb./Bot. Gar. | Unknown | DAWE: D2007-0096.002 | Xanthocarpum |
| GP018_1 | *V. opulus* | *V. opulus* | Arb./Bot. Gar. | Unknown | DAWE: D2007-0096.002 | Xanthocarpum |
| GP019 | *V. opulus* | *V. opulus* | Arb./Bot. Gar. | Known | DAWE: D2002-0454.002 |  |
| GP021 | *V. opulus* | *V. opulus* | Arb./Bot. Gar. | Unknown | DAWE: D1995-0834.002 | Aureum |
| DT030 | *V. opulus* | *V. opulus* | Arb./Bot. Gar. | Known | MORT: 229-93*1 |  |
| DT020 | *V. sargentii* | *V. sargentii* | Arb./Bot. Gar. | Known | MORT: 174-2003*1 |  |
| DT027 | *V. sargentii* | *V. sargentii* | Arb./Bot. Gar. | Known | MORT: 360-93*2 |  |
| DT024 | *V. sargentii* | *V. sargentii* | Arb./Bot. Gar. | Known | MORT: 351-81*4 |  |
| DT019 | *V. sargentii* | *V. sargentii* | Arb./Bot. Gar. | Known | MORT: 363-81*7 |  |
| DT026 | *V. sargentii* | *V. sargentii* | Arb./Bot. Gar. | Known | MORT: 361-93*2 | f. puberulum |
| DT015 | *V. trilobum* | *V. trilobum* | Arb./Bot. Gar. | Known | MORT: 441-81*10 |  |
| DT016 | *V. sargentii* | *V. sargentii* | Arb./Bot. Gar. | Known | MORT: 351-81*2 |  |
| DT017 | *V. sargentii* | *V. sargentii* | Arb./Bot. Gar. | Unknown | MORT: 372-76*1 | Flavum |
| DT018 | *V. sargentii* | *V. sargentii* | Arb./Bot. Gar. | Known | MORT: 360-93*1 | f. puberulum |
| DT021 | *V. trilobum* | *V. trilobum* | Arb./Bot. Gar. | Unknown | MORT: 579-70*1 | Red Tip |
| DT022 | *V. trilobum* | *V. trilobum* | Arb./Bot. Gar. | Unknown | MORT: 374-76*1 | Compactum |
| DT023 | *V. trilobum* | *V. trilobum* | Arb./Bot. Gar. | Known | MORT: 171-93*5 |  |
| DT025 | *V. sargentii* | *V. sargentii* | Arb./Bot. Gar. | Known | MORT: 361-93*1 |  |
| DT028 | *V. sargentii* | *V. sargentii* | Arb./Bot. Gar. | Known | MORT: 360-93*4 | f. puberulum |
| DT029 | *V. sargentii* | *V. sargentii* | Arb./Bot. Gar. | Known | MORT: 360-93*3 | f. puberulum |
| DT031 | *V. sargentii* | *V. sargentii* | Arb./Bot. Gar. | Known | MORT: 363-81*3 |  |
| DT032 | *V. sargentii* | *V. sargentii* | Arb./Bot. Gar. | Known | MORT: 363-81*1 |  |
| DT033 | *V. opulus* | *V. opulus* | Arb./Bot. Gar. | Known | MORT: 163-99*1 |  |
| DT034 | *V. sargentii* | *V. sargentii* | Arb./Bot. Gar. | Known | MORT: 351-81*1 |  |
| EE001 | *V. sargentii* | *V. sargentii* | Arb./Bot. Gar. | Unknown | POLLY: 2017-186*CX |  |
| EE002 | *V. sargentii* | Vo × Vs | Arb./Bot. Gar. | Unknown | POLLY: 1985-066*A |  |
| DT181 | *V. opulus* | *V. opulus* | Wild | Known |  |  |
| AG003 | *V. opulus* | *V. opulus* | Wild | Known |  |  |
| DT245 | *V. opulus* | *V. opulus* | Wild | Known |  |  |
| DT197 | *V. opulus* | *V. opulus* | Wild | Known |  |  |
| DT220 | *V. opulus* | *V. opulus* | Wild | Known |  |  |
| DT201 | *V. trilobum* | *V. trilobum* | Wild | Known |  |  |
| AG006 | *V. trilobum* | *V. opulus* | Wild | Known |  |  |
| DT103 | *V. trilobum* | *V. trilobum* | Wild | Known |  |  |
| DT112 | *V. trilobum* | *V. trilobum* | Wild | Known |  |  |
| DT116 | *V. trilobum* | *V. trilobum* | Wild | Known |  |  |
| DT118 | *V. trilobum* | *V. trilobum* | Wild | Known |  |  |
| DT174 | *V. trilobum* | *V. trilobum* | Wild | Known |  |  |
| DT152 | *V. trilobum* | *V. trilobum* | Wild | Known |  |  |
| DT152_1 | *V. trilobum* | *V. trilobum* | Wild | Known |  |  |
| DT125 | *V. trilobum* | *V. trilobum* | Wild | Known |  |  |
| MHM001 | Unknown | *V. opulus* | Landscape | Known |  |  |
| DT003 | Unknown | *V. opulus* | Wild | Known |  |  |
| DT004 | Unknown | *V. opulus* | Wild | Known |  |  |
| DT005 | Unknown | *V. opulus* | Wild | Known |  |  |
| DT006 | Unknown | *V. opulus* | Wild | Known |  |  |
| DT008 | *V. opulus* | *V. opulus* | Landscape | Unknown |  |  |
| DT009 | *V. opulus* | *V. opulus* | Landscape | Unknown |  |  |
| DT010 | *V. opulus* | *V. opulus* | Landscape | Unknown |  |  |
| DT011 | *V. opulus* | *V. opulus* | Landscape | Unknown |  |  |
| DT012 | *V. opulus* | *V. opulus* | Landscape | Unknown |  |  |
| DT013 | *V. opulus* | *V. opulus* | Landscape | Unknown |  |  |
| DT014 | *V. opulus* | *V. opulus* | Wild | Known |  |  |
| DT055 | Unknown | *V. trilobum* | Wild | Known |  |  |
| DT056 | *V. opulus* | *V. opulus* | Wild | Known |  |  |
| DT057 | *V. opulus* | *V. opulus* | Landscape | Unknown |  |  |
| DT058 | *V. trilobum* | *V. trilobum* | Wild | Known |  |  |
| DT059 | *V. trilobum* | *V. trilobum* | Wild | Known |  |  |
| DT060 | *V. opulus* | *V. opulus* | Wild | Known |  |  |
| DT061 | *V. opulus* | *V. opulus* | Wild | Known |  |  |
| DT062 | *V. opulus* | *V. opulus* | Wild | Known |  |  |
| DT063 | *V. trilobum* | *V. trilobum* | Wild | Known |  |  |
| DT064 | *V. trilobum* | *V. trilobum* | Wild | Known |  |  |
| DT065 | *V. trilobum* | *V. trilobum* | Wild | Known |  |  |
| DT066 | *V. opulus* | *V. opulus* | Wild | Known |  |  |
| DT066_1 | *V. opulus* | *V. opulus* | Wild | Known |  |  |
| DT067 | *V. opulus* | *V. opulus* | Wild | Known |  |  |
| DT068 | *V. opulus* | *V. opulus* | Wild | Known |  |  |
| DT069 | *V. trilobum* | *V. trilobum* | Wild | Known |  |  |
| DT070 | *V. trilobum* | *V. trilobum* | Wild | Known |  |  |
| DT071 | *V. trilobum* | *V. trilobum* | Wild | Known |  |  |
| DT072 | Unknown | *V. trilobum* | Wild | Known |  |  |
| DT073 | *V. trilobum* | *V. trilobum* | Wild | Known |  |  |
| DT074 | *V. trilobum* | *V. trilobum* | Wild | Known |  |  |
| DT075 | *V. trilobum* | *V. trilobum* | Wild | Known |  |  |
| DT076 | *V. opulus* | *V. opulus* | Wild | Known |  |  |
| DT091 | *V. opulus* | Vo × Vs | Wild | Known |  |  |
| DT092 | *V. opulus* | *V. opulus* | Wild | Known |  |  |
| DT093 | *V. trilobum* | *V. trilobum* | Landscape | Unknown |  |  |
| DT094 | *V. opulus* | Vo × Vs | Wild | Known |  |  |
| DT095 | Unknown | Vo × Vs | Wild | Known |  |  |
| DT096 | *V. trilobum* | *V. opulus* | Wild | Known |  |  |
| DT097 | Unknown | Vo × Vs | Wild | Known |  |  |
| DT098 | *V. opulus* | Vo × Vs | Wild | Known |  |  |
| DT099 | *V. opulus* | Vo × Vs | Wild | Known |  |  |
| DT100 | *V. opulus* | *V. opulus* | Wild | Known |  |  |
| DT102 | *V. opulus* | *V. opulus* | Wild | Known |  |  |
| DT104 | *V. opulus* | *V. opulus* | Wild | Known |  |  |
| DT105 | *V. opulus* | *V. opulus* | Landscape | Known |  |  |
| DT106 | *V. trilobum* | *V. trilobum* | Wild | Known |  |  |
| DT107 | *V. opulus* | Vo × Vs | Wild | Known |  |  |
| DT108 | *V. trilobum* | *V. trilobum* | Wild | Known |  |  |
| DT109 | *V. opulus* | *V. opulus* | Wild | Known |  |  |
| DT110 | *V. trilobum* | *V. trilobum* | Wild | Known |  |  |
| DT111 | *V. trilobum* | *V. trilobum* | Wild | Known |  |  |
| DT113 | *V. opulus* | *V. opulus* | Wild | Known |  |  |
| DT114 | *V. trilobum* | *V. trilobum* | Wild | Known |  |  |
| DT115 | *V. trilobum* | *V. trilobum* | Wild | Known |  |  |
| DT117 | *V. trilobum* | *V. trilobum* | Wild | Known |  |  |
| DT119 | *V. trilobum* | *V. trilobum* | Wild | Known |  |  |
| DT120 | *V. trilobum* | *V. trilobum* | Wild | Known |  |  |
| DT121 | *V. trilobum* | *V. trilobum* | Wild | Known |  |  |
| DT122 | *V. trilobum* | *V. trilobum* | Wild | Known |  |  |
| DT123 | Unknown | *V. trilobum* | Wild | Known |  |  |
| DT124 | *V. trilobum* | *V. trilobum* | Wild | Known |  |  |
| DT126 | *V. trilobum* | *V. trilobum* | Wild | Known |  |  |
| DT127 | *V. trilobum* | *V. trilobum* | Wild | Known |  |  |
| DT128 | *V. trilobum* | *V. trilobum* | Wild | Known |  |  |
| DT129 | Unknown | *V. opulus* | Wild | Known |  |  |
| DT130 | Unknown | *V. opulus* | Wild | Known |  |  |
| DT131 | Unknown | *V. trilobum* | Wild | Known |  |  |
| DT132 | *V. opulus* | *V. opulus* | Wild | Known |  |  |
| DT133 | *V. trilobum* | *V. trilobum* | Wild | Known |  |  |
| DT134 | *V. opulus* | *V. opulus* | Wild | Known |  |  |
| DT135 | *V. opulus* | *V. opulus* | Wild | Known |  |  |
| DT136 | Unknown | *V. trilobum* | Wild | Known |  |  |
| DT137 | *V. trilobum* | *V. trilobum* | Wild | Known |  |  |
| DT138 | Unknown | *V. trilobum* | Wild | Known |  |  |
| DT139 | *V. trilobum* | *V. trilobum* | Wild | Known |  |  |
| DT140 | *V. opulus* | *V. opulus* | Wild | Known |  |  |
| DT141 | *V. trilobum* | *V. trilobum* | Wild | Known |  |  |
| DT142 | *V. trilobum* | *V. trilobum* | Wild | Known |  |  |
| DT143 | *V. trilobum* | *V. trilobum* | Wild | Known |  |  |
| DT144 | *V. trilobum* | *V. trilobum* | Wild | Known |  |  |
| DT145 | *V. trilobum* | *V. trilobum* | Wild | Known |  |  |
| DT146 | Unknown | *V. trilobum* | Wild | Known |  |  |
| DT147 | *V. trilobum* | *V. trilobum* | Landscape | Unknown |  |  |
| DT148 | *V. trilobum* | *V. trilobum* | Wild | Known |  |  |
| DT149 | *V. trilobum* | *V. trilobum* | Wild | Known |  |  |
| DT150 | *V. trilobum* | *V. trilobum* | Wild | Known |  |  |
| DT151 | *V. trilobum* | *V. trilobum* | Wild | Known |  |  |
| DT101 | *V. opulus* | *V. opulus* | Wild | Known |  |  |
| DT153 | *V. trilobum* | *V. trilobum* | Wild | Known |  |  |
| DT154 | *V. trilobum* | *V. trilobum* | Wild | Known |  |  |
| DT155 | *V. trilobum* | *V. trilobum* | Wild | Known |  |  |
| DT156 | *V. trilobum* | *V. trilobum* | Wild | Known |  |  |
| DT157 | *V. trilobum* | *V. trilobum* | Wild | Known |  |  |
| DT158 | *V. trilobum* | *V. trilobum* | Wild | Known |  |  |
| DT159 | Unknown | *V. trilobum* | Wild | Known |  |  |
| DT159_1 | Unknown | *V. trilobum* | Wild | Known |  |  |
| DT160 | *V. trilobum* | *V. trilobum* | Wild | Known |  |  |
| DT161 | *V. trilobum* | *V. trilobum* | Wild | Known |  |  |
| DT162 | *V. trilobum* | *V. trilobum* | Wild | Known |  |  |
| DT163 | *V. opulus* | *V. opulus* | Landscape | Known |  |  |
| DT164 | *V. opulus* | *V. opulus* | Landscape | Known |  |  |
| DT165 | *V. opulus* | *V. opulus* | Landscape | Known |  |  |
| DT166 | *V. opulus* | *V. opulus* | Landscape | Known |  |  |
| DT167 | *V. trilobum* | *V. trilobum* | Wild | Known |  |  |
| DT168 | *V. trilobum* | *V. trilobum* | Wild | Known |  |  |
| DT169 | *V. trilobum* | *V. trilobum* | Wild | Known |  |  |
| DT170 | *V. trilobum* | *V. trilobum* | Wild | Known |  |  |
| DT171 | *V. trilobum* | *V. trilobum* | Wild | Known |  |  |
| DT172 | *V. trilobum* | *V. trilobum* | Wild | Known |  |  |
| DT173 | *V. trilobum* | *V. trilobum* | Wild | Known |  |  |
| DT175 | *V. trilobum* | *V. trilobum* | Wild | Known |  |  |
| DT176 | *V. trilobum* | *V. trilobum* | Wild | Known |  |  |
| DT177 | *V. trilobum* | *V. trilobum* | Wild | Known |  |  |
| DT178 | *V. trilobum* | *V. trilobum* | Wild | Known |  |  |
| DT179 | Unknown | *V. opulus* | Landscape | Unknown |  |  |
| DT180 | *V. opulus* | *V. opulus* | Wild | Known |  |  |
| DT182 | *V. opulus* | *V. opulus* | Wild | Known |  |  |
| DT183 | *V. opulus* | *V. opulus* | Wild | Known |  |  |
| DT185 | *V. opulus* | *V. opulus* | Wild | Known |  |  |
| DT186 | *V. opulus* | *V. opulus* | Wild | Known |  |  |
| DT187 | *V. opulus* | *V. opulus* | Wild | Known |  |  |
| DT188 | *V. opulus* | *V. opulus* | Wild | Known |  |  |
| DT189 | *V. opulus* | *V. opulus* | Wild | Known |  |  |
| DT190 | *V. opulus* | *V. opulus* | Wild | Known |  |  |
| DT191 | *V. trilobum* | *V. trilobum* | Wild | Known |  |  |
| DT192 | *V. opulus* | *V. opulus* | Wild | Known |  |  |
| DT193 | *V. opulus* | *V. opulus* | Wild | Known |  |  |
| DT194 | *V. trilobum* | *V. trilobum* | Wild | Known |  |  |
| DT195 | *V. opulus* | *V. opulus* | Wild | Known |  |  |
| DT196 | Unknown | *V. opulus* | Wild | Known |  |  |
| DT198 | *V. opulus* | *V. opulus* | Wild | Known |  |  |
| DT199 | Unknown | *V. opulus* | Wild | Known |  |  |
| DT200 | *V. trilobum* | *V. trilobum* | Wild | Known |  |  |
| DT202 | *V. opulus* | *V. opulus* | Wild | Known |  |  |
| DT203 | *V. opulus* | *V. opulus* | Wild | Known |  |  |
| DT204 | *V. opulus* | *V. opulus* | Wild | Known |  |  |
| DT205 | *V. opulus* | *V. opulus* | Wild | Known |  |  |
| DT206 | Unknown | *V. opulus* | Wild | Known |  |  |
| DT207 | *V. opulus* | *V. opulus* | Wild | Known |  |  |
| DT208 | *V. opulus* | *V. opulus* | Wild | Known |  |  |
| DT209 | Unknown | *V. opulus* | Wild | Known |  |  |
| DT210 | Unknown | *V. opulus* | Wild | Known |  |  |
| DT211 | *V. opulus* | *V. opulus* | Wild | Known |  |  |
| DT212 | *V. opulus* | *V. opulus* | Wild | Known |  |  |
| DT213 | *V. opulus* | *V. opulus* | Wild | Known |  |  |
| DT214 | Unknown | *V. opulus* | Wild | Known |  |  |
| DT215 | *V. opulus* | *V. opulus* | Wild | Known |  |  |
| DT216 | *V. opulus* | *V. opulus* | Wild | Known |  |  |
| DT217 | Unknown | *V. trilobum* | Wild | Known |  |  |
| DT218 | Unknown | *V. trilobum* | Wild | Known |  |  |
| DT219 | Unknown | *V. opulus* | Wild | Known |  |  |
| DT221 | *V. opulus* | *V. opulus* | Wild | Known |  |  |
| DT222 | *V. opulus* | *V. opulus* | Wild | Known |  |  |
| DT223 | Unknown | *V. opulus* | Wild | Known |  |  |
| DT224 | Unknown | *V. opulus* | Wild | Known |  |  |
| DT225 | *V. opulus* | *V. opulus* | Wild | Known |  |  |
| DT226 | *V. opulus* | *V. opulus* | Wild | Known |  |  |
| DT227 | *V. opulus* | *V. opulus* | Wild | Known |  |  |
| DT228 | *V. opulus* | *V. opulus* | Wild | Known |  |  |
| DT229 | *V. opulus* | *V. opulus* | Wild | Known |  |  |
| DT230 | *V. opulus* | *V. opulus* | Wild | Known |  |  |
| DT231 | Unknown | *V. trilobum* | Wild | Known |  |  |
| DT232 | Unknown | *V. opulus* | Wild | Known |  |  |
| DT233 | Unknown | *V. opulus* | Wild | Known |  |  |
| DT234 | *V. opulus* | *V. opulus* | Wild | Known |  |  |
| DT235 | *V. opulus* | *V. opulus* | Wild | Known |  |  |
| DT236 | *V. opulus* | *V. opulus* | Wild | Known |  |  |
| DT237 | *V. opulus* | *V. opulus* | Wild | Known |  |  |
| DT238 | *V. opulus* | *V. opulus* | Wild | Known |  |  |
| DT239 | *V. opulus* | *V. opulus* | Wild | Known |  |  |
| DT240 | *V. opulus* | *V. opulus* | Wild | Known |  |  |
| DT241 | *V. opulus* | *V. opulus* | Wild | Known |  |  |
| DT242 | *V. trilobum* | *V. trilobum* | Wild | Known |  |  |
| DT243 | *V. trilobum* | *V. trilobum* | Wild | Known |  |  |
| DT244 | *V. opulus* | *V. opulus* | Wild | Known |  |  |
| DT246 | *V. opulus* | *V. opulus* | Wild | Known |  |  |
| DT247 | *V. opulus* | *V. opulus* | Wild | Known |  |  |
| AGS001 | *V. opulus* | *V. opulus* | Wild | Known |  |  |
| AGS002 | *V. opulus* | Vo × Vs | Wild | Known |  |  |
| AGS003 | *V. opulus* | *V. opulus* | Wild | Known |  |  |
| AGS004 | *V. trilobum* | *V. trilobum* | Wild | Known |  |  |
| AGS005 | *V. trilobum* | *V. trilobum* | Landscape | Unknown |  |  |
| AGS006 | *V. trilobum* | *V. trilobum* | Landscape | Unknown |  |  |
| AG001 | *V. opulus* | *V. opulus* | Wild | Known |  |  |
| AG002 | *V. opulus* | *V. opulus* | Wild | Known |  |  |
| AG004 | Unknown | *V. opulus* | Wild | Known |  |  |
| AG005 | *V. trilobum* | *V. opulus* | Wild | Known |  |  |
| AG007 | *V. trilobum* | *V. opulus* | Wild | Known |  |  |
| AG008 | *V. trilobum* | *V. opulus* | Wild | Known |  |  |
| AG009 | *V. trilobum* | *V. opulus* | Wild | Known |  |  |
| AG010 | Unknown | *V. opulus* | Wild | Known |  |  |
| AG011 | *V. opulus* | *V. opulus* | Wild | Known |  |  |
| AG012 | *V. opulus* | *V. opulus* | Wild | Known |  |  |
| AG013 | *V. opulus* | *V. opulus* | Wild | Known |  |  |
| AG014 | *V. opulus* | *V. opulus* | Wild | Known |  |  |
| AG015 | *V. opulus* | *V. opulus* | Wild | Known |  |  |
| AG016 | Unknown | *V. opulus* | Wild | Known |  |  |
| AG017 | *V. opulus* | *V. opulus* | Wild | Known |  |  |
| AG018 | *V. opulus* | *V. opulus* | Wild | Known |  |  |
| AG019 | Unknown | *V. opulus* | Wild | Known |  |  |
| AG020 | *V. opulus* | *V. opulus* | Wild | Known |  |  |
| AG021 | Unknown | *V. opulus* | Wild | Known |  |  |
| AG022 | *V. opulus* | *V. opulus* | Wild | Known |  |  |
| AG023 | *V. opulus* | *V. opulus* | Wild | Known |  |  |
| MHM002 | Unknown | *V. opulus* | Landscape | Unknown |  |  |
| AGS008 | Unknown | *V. opulus* | Wild | Known |  |  |
| DT007 | Unknown | NA | Wild | Unknown |  |  |
| DT184 | *V. opulus* | NA | Wild | Known |  |  |
| NT001 | Unknown | *V. opulus* | Wild | Known |  |  |
| NT002 | Unknown | *V. opulus* | Wild | Known |  |  |
| NT003 | Unknown | *V. opulus* | Landscape | Unknown |  |  |
| NT004 | Unknown | Vo × Vs | Landscape | Unknown |  |  |
| NT005 | Unknown | *V. trilobum* | Landscape | Unknown |  |  |
| NT006 | Unknown | *V. trilobum* | Wild | Known |  |  |
| NT007 | Unknown | *V. trilobum* | Wild | Known |  |  |
| NT008 | Unknown | *V. trilobum* | Landscape | Unknown |  |  |
| JC002 | *V. opulus* | *V. opulus* | Wild | Known |  |  |
| AS002 | *V. trilobum* | *V. trilobum* | Arb./Bot. Gar. | Known | NCRPIS: Ames 32966 |  |
| AS021 | *V. trilobum* | *V. trilobum* | Arb./Bot. Gar. | Known | NCRPIS: 198-6 |  |
| JC001 | *V. opulus* | *V. opulus* | Wild | Known |  |  |
| JC003 | *V. opulus* | *V. opulus* | Wild | Known |  |  |
| AS001 | *V. trilobum* | *V. trilobum* | Arb./Bot. Gar. | Known | NCRPIS: Ames 32966 |  |
| AS003 | *V. trilobum* | *V. trilobum* | Arb./Bot. Gar. | Known | NCRPIS: Ames 32966 |  |
| AS005 | *V. trilobum* | *V. trilobum* | Arb./Bot. Gar. | Known | NCRPIS: Ames 32966 |  |
| AS007 | *V. trilobum* | *V. trilobum* | Arb./Bot. Gar. | Known | NCRPIS: Ames 32966 |  |
| AS008 | *V. trilobum* | *V. trilobum* | Arb./Bot. Gar. | Known | NCRPIS: Ames 32966 |  |
| AS009 | *V. trilobum* | *V. trilobum* | Arb./Bot. Gar. | Known | NCRPIS: Ames 32966 |  |
| AS010 | *V. trilobum* | *V. trilobum* | Arb./Bot. Gar. | Known | NCRPIS: Ames 32966 |  |
| AS011 | *V. trilobum* | *V. trilobum* | Arb./Bot. Gar. | Known | NCRPIS: Ames 32966 |  |
| AS012 | *V. trilobum* | *V. trilobum* | Arb./Bot. Gar. | Known | NCRPIS: Ames 32966 |  |
| AS013 | *V. trilobum* | *V. trilobum* | Arb./Bot. Gar. | Known | NCRPIS: Ames 32966 |  |
| AS014 | *V. trilobum* | *V. trilobum* | Arb./Bot. Gar. | Known | NCRPIS: Ames 32966 |  |
| AS015 | *V. trilobum* | *V. trilobum* | Arb./Bot. Gar. | Known | NCRPIS: Ames 32966 |  |
| AS016 | *V. trilobum* | *V. trilobum* | Arb./Bot. Gar. | Known | NCRPIS: 198-1 |  |
| AS017 | *V. trilobum* | *V. trilobum* | Arb./Bot. Gar. | Known | NCRPIS: 198-2 |  |
| AS018 | *V. trilobum* | *V. trilobum* | Arb./Bot. Gar. | Known | NCRPIS: 198-3 |  |
| AS019 | *V. trilobum* | *V. trilobum* | Arb./Bot. Gar. | Known | NCRPIS: 198-4 |  |
| AS020 | *V. trilobum* | *V. trilobum* | Arb./Bot. Gar. | Known | NCRPIS: 198-5 |  |
| AS022 | *V. trilobum* | *V. trilobum* | Arb./Bot. Gar. | Known | NCRPIS: 197-1 |  |
| AS023 | *V. trilobum* | *V. trilobum* | Arb./Bot. Gar. | Known | NCRPIS: 197-2 |  |
| AS024 | *V. trilobum* | *V. trilobum* | Arb./Bot. Gar. | Known | NCRPIS: 197-3 |  |
| AS025 | *V. trilobum* | *V. trilobum* | Arb./Bot. Gar. | Known | NCRPIS: 207-1 |  |
| AS026 | *V. opulus* | *V. opulus* | Wild | Known |  |  |
| AS027 | *V. opulus* | *V. opulus* | Wild | Known |  |  |
| AS004 | *V. trilobum* | NA | Arb./Bot. Gar. | Known | NCRPIS: Ames 32966 |  |
| AS006 | *V. trilobum* | NA | Arb./Bot. Gar. | Known | NCRPIS: Ames 32966 |  |
| TR011 | *V. opulus* | *V. opulus* | Arb./Bot. Gar. | Known | USNA: NA 66971-02 |  |
| TR016 | *V. sargentii* | *V. sargentii* | Arb./Bot. Gar. | Known | USNA: NA 67733-02 |  |
| TR013 | *V. sargentii* | *V. sargentii* | Arb./Bot. Gar. | Known | USNA: NA 64609-01 |  |
| TR008 | *V. sargentii* | *V. sargentii* | Arb./Bot. Gar. | Known | USNA: NA 55064-04 |  |
| TR001 | *V. sargentii* | *V. sargentii* | Arb./Bot. Gar. | Known | USNA: NA 69086-01 |  |
| TR005 | *V. sargentii* | *V. sargentii* | Arb./Bot. Gar. | Known | USNA: NA 63296-02 |  |
| TR002 | *V. sargentii* | *V. sargentii* | Arb./Bot. Gar. | Known | USNA: NA 68866-02 |  |
| TR003 | *V. sargentii* | *V. sargentii* | Arb./Bot. Gar. | Known | USNA: NA 64165-02 |  |
| TR004 | *V. sargentii* | *V. sargentii* | Arb./Bot. Gar. | Known | USNA: NA 64190-01 |  |
| TR006 | *V. sargentii* | *V. sargentii* | Arb./Bot. Gar. | Known | USNA: NA 61695-02 |  |
| TR007 | *V. sargentii* | *V. sargentii* | Arb./Bot. Gar. | Known | USNA: NA 61728-03 | var. calvescens |
| TR009 | *V. trilobum* | *V. opulus* | Arb./Bot. Gar. | Known | USNA: NA 73649-02 |  |
| TR010 | *V. opulus* | *V. opulus* | Arb./Bot. Gar. | Known | USNA: NA 66971-01 |  |
| TR012 | *V. opulus* | *V. opulus* | Arb./Bot. Gar. | Known | USNA: NA 70015-01 |  |
| TR014 | *V. opulus* | *V. opulus* | Arb./Bot. Gar. | Known | USNA: NA 66970-08 |  |
| TR015 | *V. opulus* | *V. sargentii* | Arb./Bot. Gar. | Known | USNA: NA 66971-07 |  |
| TR017 | *V. sargentii* | *V. sargentii* | Arb./Bot. Gar. | Known | USNA: NA 64609 J PL |  |
| TR018 | *V. opulus* | *V. opulus* | Arb./Bot. Gar. | Known | USNA: NA 66970-06 PL |  |
| JM010 | *V. opulus* | *V. opulus* | Wild | Known |  |  |
| JM006 | *V. trilobum* | *V. trilobum* | Wild | Known |  |  |
| JM029 | *V. trilobum* | *V. trilobum* | Wild | Known |  |  |
| JGr001 | *V. opulus* | *V. opulus* | Landscape | Unknown |  |  |
| JGr002 | *V. opulus* | *V. trilobum* | Wild | Known |  |  |
| JGr003 | Unknown | *V. trilobum* | Wild | Known |  |  |
| NQ001 | *V. trilobum* | *V. trilobum* | Wild | Known |  |  |
| NQ002 | *V. trilobum* | *V. trilobum* | Wild | Known |  |  |
| NQ003 | *V. trilobum* | *V. trilobum* | Wild | Known |  |  |
| NQ004 | *V. trilobum* | *V. trilobum* | Wild | Known |  |  |
| NQ005 | Unknown | *V. trilobum* | Wild | Known |  |  |
| JM001 | *V. trilobum* | *V. trilobum* | Landscape | Known |  |  |
| JM002 | *V. trilobum* | *V. trilobum* | Wild | Known |  |  |
| JM003 | *V. trilobum* | *V. trilobum* | Wild | Known |  |  |
| JM004 | *V. trilobum* | *V. trilobum* | Wild | Known |  |  |
| JM005 | *V. trilobum* | *V. trilobum* | Wild | Known |  |  |
| JM007 | *V. trilobum* | *V. trilobum* | Wild | Known |  |  |
| JM008 | *V. trilobum* | *V. trilobum* | Wild | Known |  |  |
| JM009 | *V. opulus* | *V. trilobum* | Wild | Known |  |  |
| JM011 | *V. trilobum* | *V. trilobum* | Wild | Known |  |  |
| JM012 | *V. trilobum* | *V. trilobum* | Wild | Known |  |  |
| JM013 | *V. trilobum* | *V. trilobum* | Wild | Known |  |  |
| JM014 | *V. trilobum* | *V. trilobum* | Wild | Known |  |  |
| JM015 | *V. trilobum* | *V. trilobum* | Wild | Known |  |  |
| JM016 | *V. trilobum* | *V. trilobum* | Wild | Known |  |  |
| JM017 | *V. trilobum* | *V. trilobum* | Wild | Known |  |  |
| JM018 | *V. trilobum* | *V. trilobum* | Wild | Known |  |  |
| JM018_1 | *V. trilobum* | *V. trilobum* | Wild | Known |  |  |
| JM019 | *V. trilobum* | *V. trilobum* | Wild | Known |  |  |
| JM020 | *V. trilobum* | *V. trilobum* | Wild | Known |  |  |
| JM021 | *V. trilobum* | *V. trilobum* | Wild | Known |  |  |
| JM022 | *V. trilobum* | *V. trilobum* | Wild | Known |  |  |
| JM023 | *V. trilobum* | *V. trilobum* | Wild | Known |  |  |
| JM024 | *V. trilobum* | *V. trilobum* | Wild | Known |  |  |
| JM025 | *V. trilobum* | *V. trilobum* | Wild | Known |  |  |
| JM026 | *V. trilobum* | *V. trilobum* | Wild | Known |  |  |
| JM027 | *V. trilobum* | *V. trilobum* | Wild | Known |  |  |
| JM028 | *V. trilobum* | *V. trilobum* | Wild | Known |  |  |
| JM030 | *V. trilobum* | *V. trilobum* | Wild | Known |  |  |
| JM031 | *V. trilobum* | *V. trilobum* | Wild | Known |  |  |
| JM032 | *V. trilobum* | *V. trilobum* | Landscape | Unknown |  |  |
| BS005 | Unknown | *V. trilobum* | Wild | Known |  |  |
| BS001 | *V. trilobum* | *V. trilobum* | Wild | Known |  |  |
| BS002 | *V. trilobum* | *V. trilobum* | Wild | Known |  |  |
| BS003 | Unknown | *V. opulus* | Wild | Known |  |  |
| BS004 | Unknown | *V. trilobum* | Wild | Known |  |  |
| BS006 | Unknown | *V. opulus* | Wild | Known |  |  |
| BS007 | Unknown | *V. opulus* | Wild | Known |  |  |

**AA:** The Arnold Arboretum of Harvard University

**AMU**: Adam Mickiewicz University Botanical Garden

**BATU:** Batumi Botanic Garden

**BBG:** Bangsbo Botanic Garden

**BERG:** Bergius Botanic Garden

**BREN:** The Brenton Arboretum

**CBG:** Chicago Botanic Garden

**CFC:** University of Minnesota Cloquet Forestry Center Arboretum

**CMBG:** Coastal Maine Botanic Gardens

**DAWE:** The Dawes Arboretum

**GEO:** Geographic Garden (Geografisk Have)

**GOTH:** Gothenburg Botanical Garden

**HOW:** Howick Hall Gardens and Arboretum

**K:** Kew Herbarium

**KAIS:** Kaisaniemi Botanic Garden

**KUMP:** Kumpula Botanic Garden

**MBG:** Missouri Botanical Garden

**MEISE:** Meise Botanic Garden

**MIN:** Bell Museum Herbarium

**MNLA:** Minnesota Landscape Arboretum

**MOR:** Morton Arboretum Herbarium

**MORT:** Morton Arboretum Living Collections

**NCRPIS:** USDA-ARS, North Central Regional Plant Introduction Station

**PAS:** Polish Academy of Sciences Botanical Garden

**POLLY:** Polly Hill Arboretum

**RBGE:** Royal Botanic Garden Edinburgh

**SBG:** Sonoma Botanical Garden

**SFA:** Starhill Forest Arboretum

**SZAR:** Szarvasi Arboretum

**USNA:** USDA-ARS, U.S. National Arboretum

**WESPE:** Arboretum Wespelaar,

**WIS:** Longenecker Horticultural Gardens, University of Wisconsin-Madison Arboretum

**WOJ:** Arboretum Wojsławice
