# Supplementary material for: American highbush cranberry maintains strong population structure despite naturalization of Eurasian relatives in North America
Source: Am J Bot. 2025 Nov 14;112(11):e70124. doi: 10.1002/ajb2.70124 (PMC12640478; doi:10.1002/ajb2.70124)
Supplement: Supplementary file 2 — Appendix S2. Principal component analysis (PCA) with reported highbush cranberry (Viburnum spp.) identifications based on visual field identification and/or database records. [file AJB2-112-e70124-s003.docx]

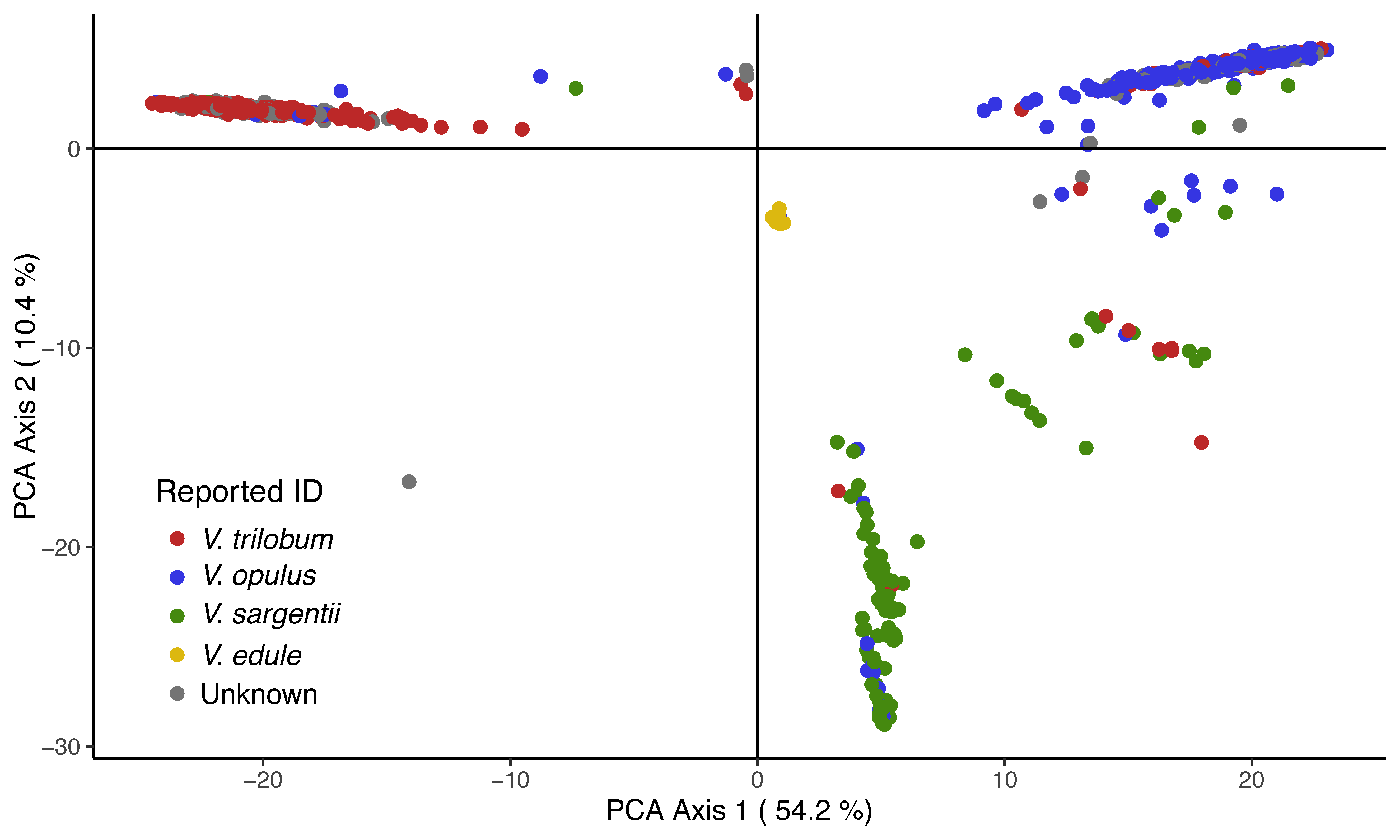


**Appendix S2.** Principal component analysis (PCA) with reported highbush cranberry (*Viburnum* spp.) identifications based on visual field identification and/or database records.
