# Supplementary material for: American highbush cranberry maintains strong population structure despite naturalization of Eurasian relatives in North America
Source: Am J Bot. 2025 Nov 14;112(11):e70124. doi: 10.1002/ajb2.70124 (PMC12640478; doi:10.1002/ajb2.70124)
Supplement: Supplementary file 3 — Appendix S3. ΔK support for number of clusters (K) for STRUCTURE analysis of all highbush cranberry (Viburnum spp.) specimens. [file AJB2-112-e70124-s007.docx]

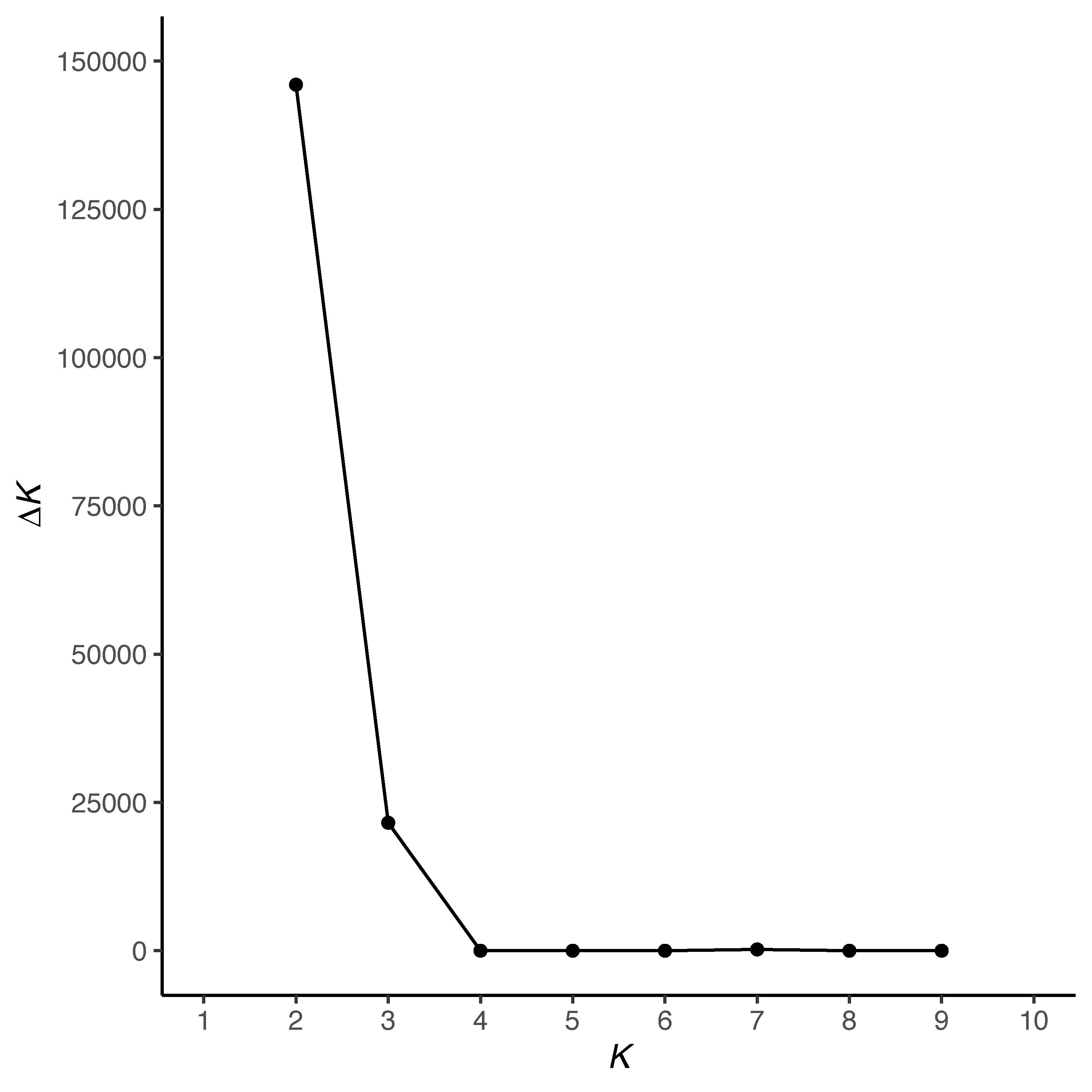


**Appendix S3.**  Δ*K* support for number of clusters (*K*) for STRUCTURE analysis of all highbush cranberry (*Viburnum* spp.) specimens.
