# Supplementary material for: American highbush cranberry maintains strong population structure despite naturalization of Eurasian relatives in North America
Source: Am J Bot. 2025 Nov 14;112(11):e70124. doi: 10.1002/ajb2.70124 (PMC12640478; doi:10.1002/ajb2.70124)
Supplement: Supplementary file 4 — Appendix S4. Separate structure analyses evaluating pairwise groupings of three main genetically identified species groups Viburnum trilobum, V. opulus, V. sargentii. Statistical support (ΔK) for the number of clusters is shown in A–C, while the membership proportions of all individuals for K = 2 clusters is shown in D–F. Admixed zones are defined by dashed vertical lines. The analysis of V. trilobum, V. opulus and their hybrids (Vt × Vo) is shown in panels A, D; V. opulus, V. sargentii and their hybrids (Vo × Vs) are shown in panels B, E; V. trilobum, V. sargentii, and their hybrids (Vt × Vs) are shown in panels C, F. [file AJB2-112-e70124-s006.docx]

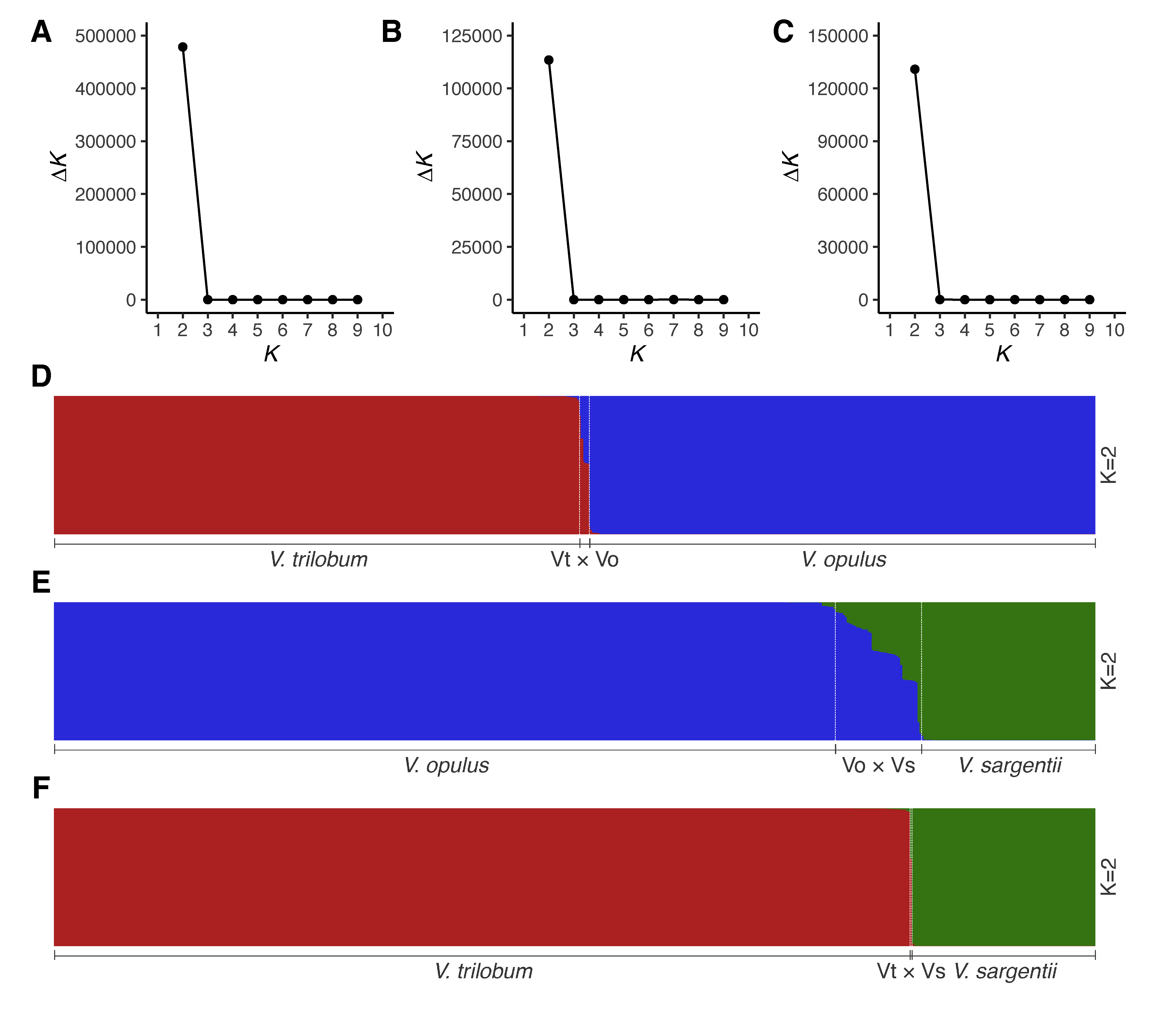


**Appendix S4.** Separate STRUCTURE analyses evaluating pairwise groupings of three main genetically identified species groups *Viburnum trilobum*, *V. opulus*, *V. sargentii*. Statistical support (Δ*K*) for the number of clusters is shown in A–C, while D–F display the membership proportions for all individuals for *K* = 2 clusters. Admixed zones are defined by dashed vertical lines. The analysis of *V. trilobum*, *V. opulus* and their hybrids (Vt × Vo) is shown in panels A, D; *V. opulus*, *V. sargentii* and their hybrids (Vo × Vs) are shown in panels B, E; *V. trilobum*, *V. sargentii*, and their hybrids (Vt × Vs) are shown in panels C, F.
