# Supplementary material for: American highbush cranberry maintains strong population structure despite naturalization of Eurasian relatives in North America
Source: Am J Bot. 2025 Nov 14;112(11):e70124. doi: 10.1002/ajb2.70124 (PMC12640478; doi:10.1002/ajb2.70124)
Supplement: Supplementary file 5 — Appendix S5. Principal component analysis (PCA) axes 1 and 3 for 995 highbush cranberry specimens, with populations assigned based on genetic identifications from structure using K = 4 clusters. Hybrid groups include V. trilobum × V. opulus (Vt × Vo), V. trilobum × V. sargentii (Vt × Vs), and V. opulus × V. sargentii (Vo × Vs). [file AJB2-112-e70124-s005.docx]

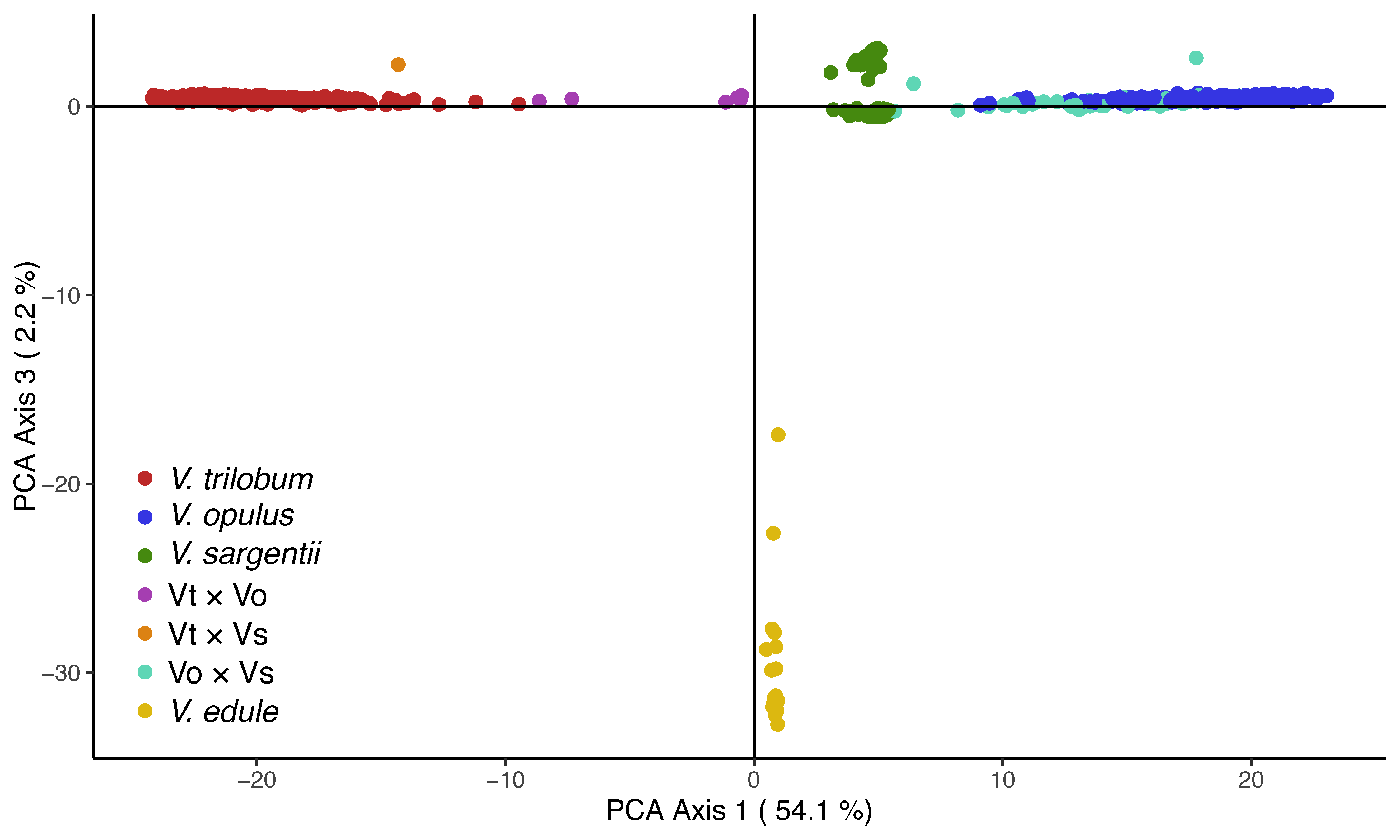


**Appendix S5.** Principal component analysis (PCA) axes 1 and 3 for 995 highbush cranberry specimens, with populations assigned based on genetic identifications from STRUCTURE using *K* = 4 clusters. Hybrid groups include *Viburnum trilobum × V. opulus* (Vt × Vo), *V. trilobum* × *V. sargentii* (Vt × Vs), and *V. opulus* × *V. sargentii* (Vo × Vs).
