# Supplementary figures and images for: American highbush cranberry maintains strong population structure despite naturalization of Eurasian relatives in North America
Source: Am J Bot. 2025 Nov 14;112(11):e70124. doi: 10.1002/ajb2.70124 (PMC12640478; doi:10.1002/ajb2.70124)

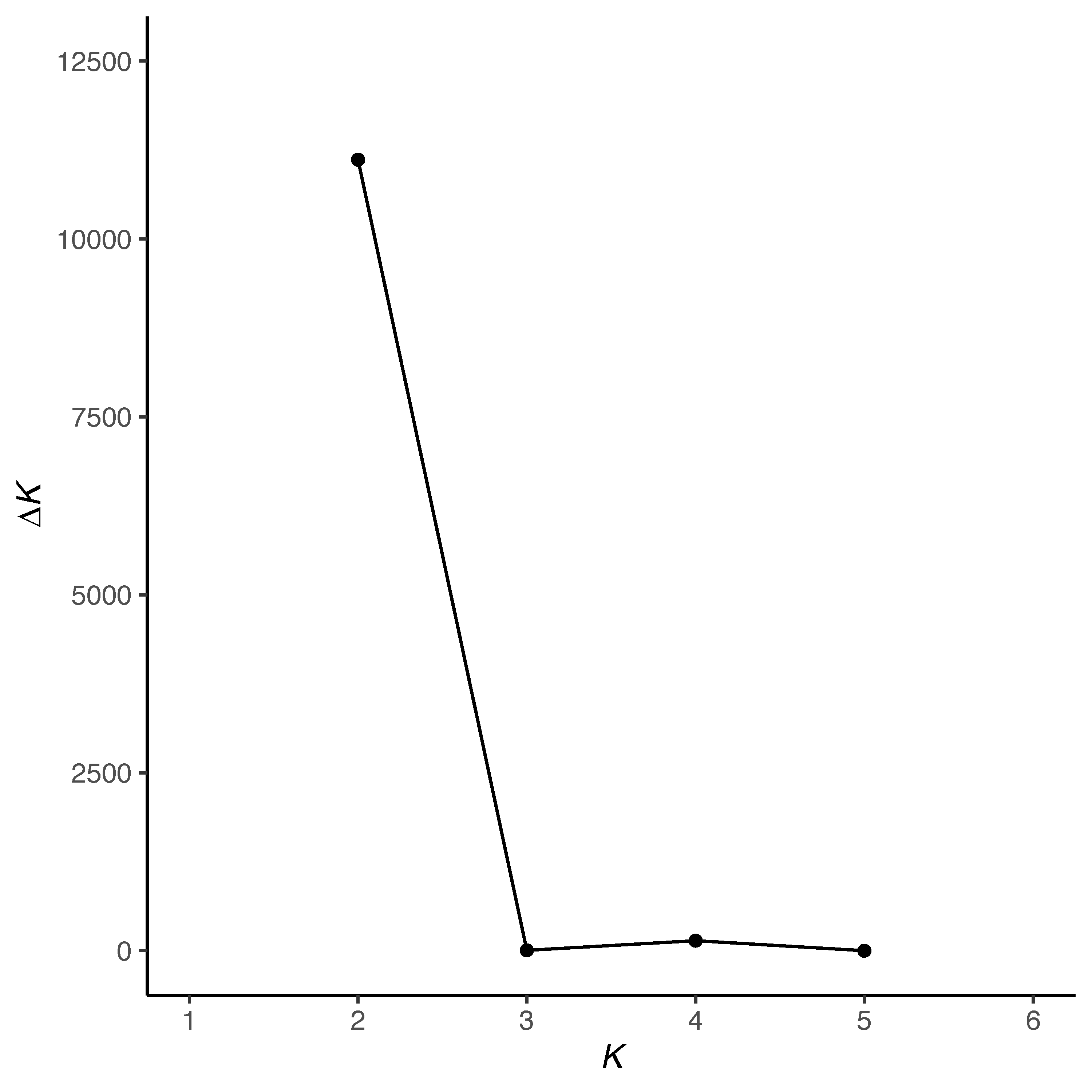


**Appendix S6.**  Δ*K* support for number of clusters (*K*) for STRUCTURE analysis of *Viburnum sargentii*.

Supplement: Supplementary file 6 — Appendix S6. ΔK support for number of clusters (K) for STRUCTURE analysis of Viburnum sargentii. [file AJB2-112-e70124-s004.docx]
