# Supplementary material for: American highbush cranberry maintains strong population structure despite naturalization of Eurasian relatives in North America
Source: Am J Bot. 2025 Nov 14;112(11):e70124. doi: 10.1002/ajb2.70124 (PMC12640478; doi:10.1002/ajb2.70124)
Supplement: Supplementary file 7 — Appendix S7. Analysis of substructure in Viburnum trilobum and V. opulus; (A) ΔK support for number of clusters (K) in V. trilobum; (B) average structure cluster assignments for each V. trilobum population plotted by location; (C) ΔK support for number of clusters (K) in V. opulus; (D) average structure cluster assignments for each V. opulus population plotted by location. [file AJB2-112-e70124-s001.docx]

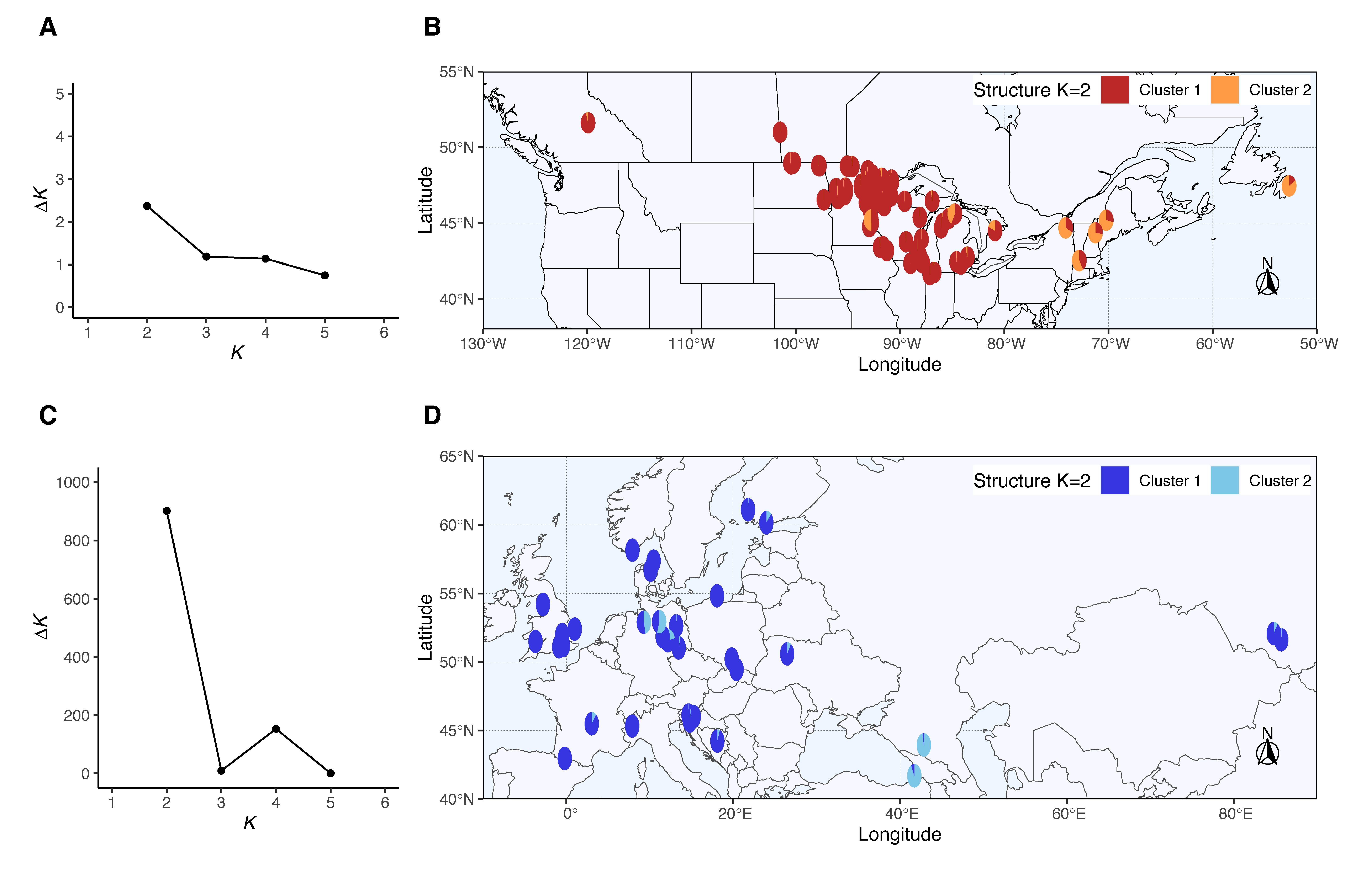


**Appendix S7.** Analysis of substructure in *Viburnum trilobum* and *V. opulus*; A) Δ*K* support for number of clusters (*K*) in *V. trilobum*; B) average STRUCTURE cluster assignments for each *V. trilobum* population plotted by location; C) Δ*K* support for number of clusters (*K*) in *V. opulus*; D) average STRUCTURE cluster assignments for each *V. opulus* population plotted by location.
