# Supplementary material for: American highbush cranberry maintains strong population structure despite naturalization of Eurasian relatives in North America
Source: Am J Bot. 2025 Nov 14;112(11):e70124. doi: 10.1002/ajb2.70124 (PMC12640478; doi:10.1002/ajb2.70124)
Supplement: Supplementary file 8 — Appendix S8. Histogram showing bitwise distance distribution for pairwise comparisons among all highbush cranberry samples. The number of estimated multilocus lineages for nearest neighbor (green), average neighbor (UPGMA or Unweighted Pair Group Method with Arithmetic Mean; blue), and farthest neighbor (red) are shown for various genetic distance thresholds. [file AJB2-112-e70124-s002.docx]

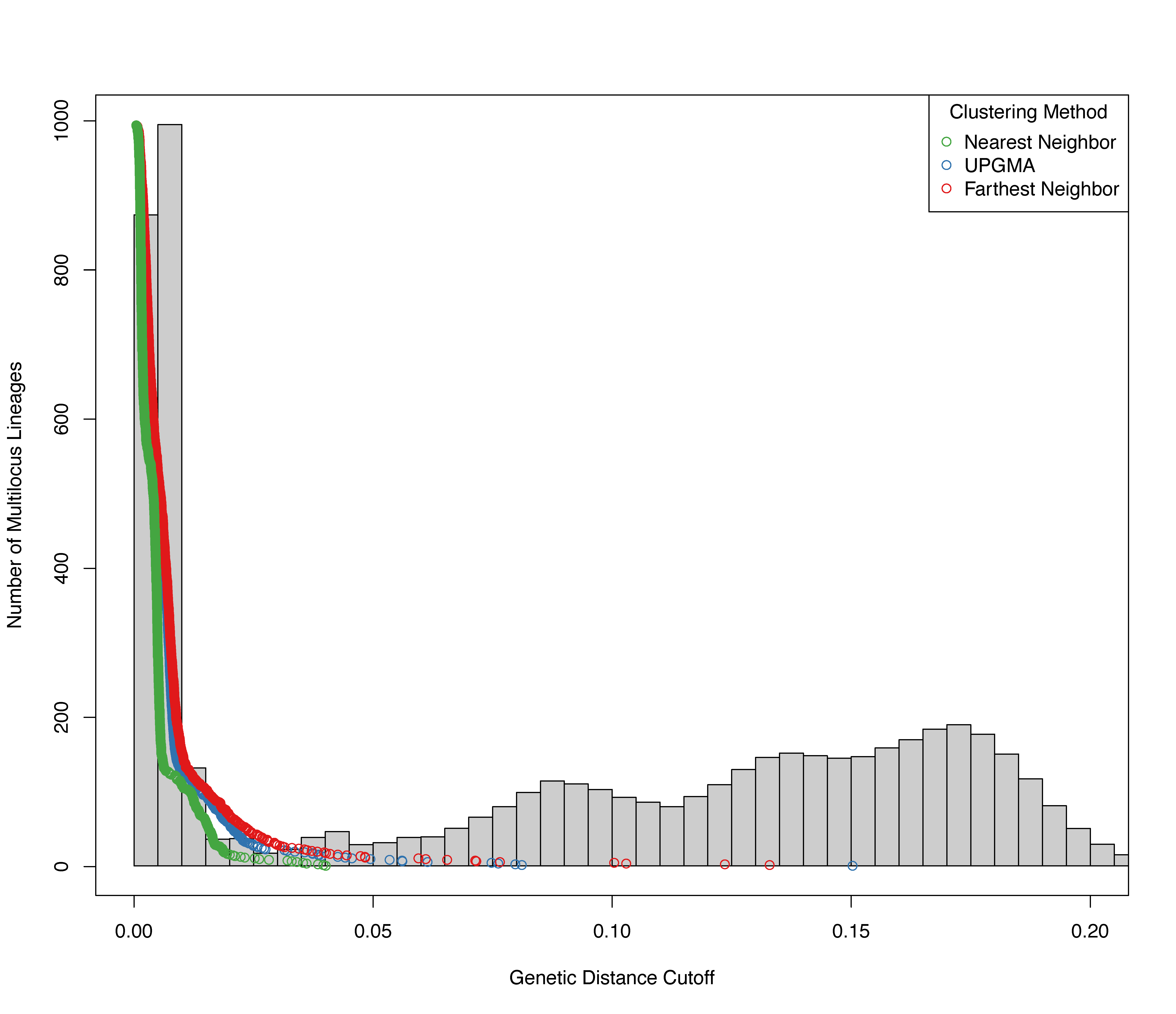


**Appendix S8.** Histogram showing bitwise distance distribution for pairwise comparisons among all highbush cranberry samples. The number of estimated multilocus lineages for nearest neighbor (green), average neighbor (UPGMA or Unweighted Pair Group Method with Arithmetic Mean; blue), and farthest neighbor (red) are shown for various genetic distance thresholds.
