# Supplementary material for: American highbush cranberry maintains strong population structure despite naturalization of Eurasian relatives in North America
Source: Am J Bot. 2025 Nov 14;112(11):e70124. doi: 10.1002/ajb2.70124 (PMC12640478; doi:10.1002/ajb2.70124)
Supplement: Supplementary file 9 — Appendix S9. List of Viburnum sargentii specimens including unique sample ID, whether the provenance is known or unknown, institution code (see footer) and accession number, and structure q‐values indicating each specimen's estimated proportion of membership in the mainland (Cluster 1) and Japan (Cluster 2) genetic clusters. [file AJB2-112-e70124-s009.docx]

**Appendix S9.** List of *Viburnum sargentii* specimens including unique sample ID, whether the provenance is known or unknown, institution code (see footer) and accession number, and STRUCTURE q-values indicating each specimen’s estimated proportion of membership in the mainland (Cluster 1) and Japan (Cluster 2) genetic clusters.

| Sample ID | Provenance | Inst. Code: Accession No. | STRUCTURE q-values | |
| --- | --- | --- | --- | --- |
|  |  |  | Cluster 1 (mainland) | Cluster 2 (Japan) |
| DJ001 | Known | AA: 287-2002*A | 0 | 1 |
| DJ002 | Known | AA: 287-2002*B | 0 | 1 |
| DJ003 | Known | AA: 87-2015*A | 1 | 0 |
| DJ004 | Known | AA: 87-2015*B | 1 | 0 |
| DJ005 | Known | AA: 87-2015*C | 1 | 0 |
| DJ006 | Known | AA: 87-2015*D | 1 | 0 |
| DJ012 | Known | AA: 719-88*A | 0 | 1 |
| DJ013 | Known | AA: 719-88*B | 0 | 1 |
| DJ014 | Known | AA: 719-88*C | 0 | 1 |
| DJ019 | Known | AA: 1922-80*A | 0.999 | 0.001 |
| DJ020 | Known | AA: 1922-80*C | 0.999 | 0.001 |
| DJ021 | Known | AA: 379-97*A | 1 | 0 |
| DJ022 | Known | AA: 379-97*B | 1 | 0 |
| DJ023 | Known | AA: 1897-80*B | 0.996 | 0.004 |
| DJ024 | Known | AA: 1897-80*C | 0.994 | 0.006 |
| DJ025 | Known | AA: 79-90*A | 1 | 0 |
| DJ026 | Known | AA: 79-90*B | 1 | 0 |
| DJ029 | Unknown | AA: 398-68*B | 0 | 1 |
| DT016 | Known | MORT: 351-81*2 | 1 | 0 |
| DT017 | Unknown | MORT: 372-76*1 | 1 | 0 |
| DT018 | Known | MORT: 360-93*1 | 1 | 0 |
| DT019 | Known | MORT: 363-81*7 | 0 | 1 |
| DT020 | Known | MORT: 174-2003*1 | 1 | 0 |
| DT024 | Known | MORT: 351-81*4 | 1 | 0 |
| DT025 | Known | MORT: 361-93*1 | 1 | 0 |
| DT026 | Known | MORT: 361-93*2 | 1 | 0 |
| DT027 | Known | MORT: 360-93*2 | 1 | 0 |
| DT028 | Known | MORT: 360-93*4 | 1 | 0 |
| DT029 | Known | MORT: 360-93*3 | 1 | 0 |
| DT031 | Known | MORT: 363-81*3 | 0.001 | 0.999 |
| DT032 | Known | MORT: 363-81*1 | 0.001 | 0.999 |
| DT034 | Known | MORT: 351-81*1 | 1 | 0 |
| EE001 | Unknown | POLLY: 2017-186*CX | 0.98 | 0.02 |
| GP003 | Unknown | DAWE: D1999-1608.001 | 0 | 1 |
| GP004 | Unknown | DAWE: D2000-0965.001 | 0 | 1 |
| GP005 | Unknown | DAWE: D1995-0668.002 | 1 | 0 |
| GP006 | Unknown | DAWE: D1995-0668.003 | 1 | 0 |
| GP007 | Known | DAWE: D1994-0680.003 | 1 | 0 |
| GP009 | Known | DAWE: D1994-0680.001 | 1 | 0 |
| GP010 | Known | DAWE: D1994-0680.002 | 1 | 0 |
| GP017 | Unknown | DAWE: D1988-0740.001 | 1 | 0 |
| HV002 | Known | KUMP: 2008-0119 | 0.994 | 0.006 |
| HV009 | Known | KUMP: 1994-0930 | 1 | 0 |
| JCz003 | Unknown | WIS: 1980-214*A | 0 | 1 |
| JCz005 | Unknown | WIS: JCz005 | 0 | 1 |
| JCz006 | Unknown | WIS: 1995-171 | 1 | 0 |
| JO005 | Unknown | WESPE: 88503 | 0 | 1 |
| MOR017 | Unknown | MOR: 91193 | 0 | 1 |
| MW001 | Known | SBG: 2001.178A | 1 | 0 |
| MW002 | Known | SBG: 2011.250A | 0 | 1 |
| PB005 | Unknown | RBGE: 19608006*A | 0.016 | 0.984 |
| PB006 | Unknown | RBGE: 19608006*B | 0.001 | 0.999 |
| PB014 | Known | RBGE: 20031031*A | 0.192 | 0.808 |
| PB015 | Known | RBGE: 20031031*A | 0.21 | 0.79 |
| PB016 | Known | RBGE: 20031031*A | 0.148 | 0.852 |
| PB018 | Unknown | RBGE: 20031296*B | 0.999 | 0.001 |
| PB019 | Known | RBGE: 20051944*A | 0 | 1 |
| PB020 | Known | RBGE: 20051944*B | 0 | 1 |
| PB021 | Known | RBGE: 20051944*C | 0 | 1 |
| PB022 | Known | RBGE: 20051944*D | 0 | 1 |
| PB023 | Known | RBGE: 20051944*E | 0 | 1 |
| PB024 | Known | RBGE: 20051944*K | 0 | 1 |
| PB025 | Known | RBGE: 20051944*L | 0 | 1 |
| PB026 | Known | RBGE: 20051944*M | 0 | 1 |
| PB027 | Known | RBGE: 20052099*A | 0.022 | 0.978 |
| PB028 | Known | RBGE: 20052099*B | 0.03 | 0.97 |
| PB029 | Known | RBGE: 20052099*L | 0.007 | 0.993 |
| PB030 | Known | RBGE: 20052099*M | 0.049 | 0.951 |
| PB031 | Known | RBGE: 20052099*P | 0.019 | 0.981 |
| PB032 | Known | RBGE: 20052099*V | 0 | 1 |
| PB033 | Known | RBGE: 20052099*W | 0 | 1 |
| PB034 | Known | RBGE: 20052099*X | 0.03 | 0.97 |
| PB035 | Known | RBGE: 20052099*Y | 0.039 | 0.961 |
| PB036 | Known | RBGE: 20052099*Z | 0.049 | 0.951 |
| PB044 | Known | RBGE: 20151211*C | 0 | 1 |
| PB045 | Known | RBGE: 20151211*D | 0 | 1 |
| PB046 | Known | RBGE: 20151211*E | 0 | 1 |
| RJ013 | Known | HOW: SICH 1856 | 1 | 0 |
| RJ016 | Known | HOW: SICH 2038 | 0.999 | 0.001 |
| TR001 | Known | USNA: NA 69086-01 | 0.339 | 0.661 |
| TR002 | Known | USNA: NA 68866-02 | 1 | 0 |
| TR003 | Known | USNA: NA 64165-02 | 1 | 0 |
| TR004 | Known | USNA: NA 64190-01 | 1 | 0 |
| TR005 | Known | USNA: NA 63296-02 | 0.01 | 0.99 |
| TR006 | Known | USNA: NA 61695-02 | 1 | 0 |
| TR007 | Known | USNA: NA 61728-03 | 1 | 0 |
| TR008 | Known | USNA: NA 55064-04 | 1 | 0 |
| TR013 | Known | USNA: NA 64609-01 | 1 | 0 |
| TR015 | Known | USNA: NA 66971-07 | 0.971 | 0.029 |
| TR016 | Known | USNA: NA 67733-02 | 0.999 | 0.001 |
| TR017 | Known | USNA: NA 64609 J PL | 1 | 0 |
| **AA:** The Arnold Arboretum of Harvard University  **DAWE:** The Dawes Arboretum  **HOW:** Howick Hall Gardens and Arboretum  **KUMP:** Kumpula Botanic Garden  **MOR:** Morton Arboretum Herbarium  **MORT:** Morton Arboretum Living Collections  **POLLY:** Polly Hill Arboretum  **RBGE:** Royal Botanic Garden Edinburgh  **SBG:** Sonoma Botanical Garden  **USNA:** USDA-ARS, U.S. National Arboretum  **WESPE:** Arboretum Wespelaar,  **WIS:** Longenecker Horticultural Gardens, University of Wisconsin-Madison Arboretum | | | | |
